# The efficacy of cognitive behavioral therapy for mental health and quality of life among individuals diagnosed with cancer: A systematic review and meta‐analysis

**DOI:** 10.1002/cam4.70063

**Published:** 2024-08-21

**Authors:** Alexander T. Dils, Kathryn O'Keefe, Nada Dakka, Michelle Azar, Meiyan Chen, Anao Zhang

**Affiliations:** ^1^ Central Michigan University College of Medicine Saginaw Michigan USA; ^2^ The University of Texas at Austin Steve Hicks School of Social Work Austin Texas USA; ^3^ University of Michigan Health, Adolescent and Young Adult Oncology Program Ann Arbor Michigan USA; ^4^ University of Michigan School of Social Work Ann Arbor Michigan USA

**Keywords:** cancer survivor, cognitive behavioral therapy, meta‐analysis, patient‐reported outcome, supportive care

## Abstract

**Objective:**

It has long been documented that cognitive behavioral therapy (CBT) has positive impacts on improving mental health (MH) and quality of life (QoL) in the general population, but investigations on its effect on cancer survivors remain limited, especially for QoL outcomes. The purpose of this meta‐analysis is to investigate the effects of CBT as compared to control on cancer patients' MH and QoL outcomes. Control is defined in this study as standard therapy, waitlist control, and active/alternative therapy.

**Methods:**

In total, 154 clinical trials creating a sample size of 1627 individuals were collected. Analysis focusing on MH and QoL excluded 29 clinical trials resulting in a final analysis of 132 clinical trials (and 1030 effect sizes). R Statistical Software (version 4.2.2) and the robumeta package were utilized to complete analysis, which entailed robust variance estimation (RVE) in intercept‐only meta‐regression, and univariate meta‐regression (for moderator analysis).

**Results:**

Across 132 clinical trials and 1030 effect size estimates, we identified that CBT moderately improves MH and QoL in cancer patients *d* = 0.388, 95% CI 0.294–0.483, *p* < 0.001. Additionally, age and delivery format can influence the efficacy of CBT in this patient population.

**Conclusions:**

CBT statistically improves the MH and QoL psychosocial parameters in cancer patients with greater efficacy in younger patients. Important clinical and intervention‐related factors, that is, age and delivery, should be considered when oncologists consider CBT as a psychotherapeutic intervention for individuals with cancer.

## 1 INTRODUCTION

Cancer is a debilitating disease that affects millions of individuals each year around the globe. While treatments have advanced in recent decades improving longevity and survival rates, treatment regimens have serious impacts on the physical, psychological, and social well‐being of oncology patients.^1^, ^2^ These patients are prone to many psychological and mental health (MH) disturbances, including depression, anxiety, and decreased general wellness, due to these psychosocial burdens associated with cancer treatment.^3^ This effect can persist after treatment has ended, and cohort studies report higher incidences of anxiety and depression in long‐term cancer survivors, with symptoms lasting for roughly 2 years or more after diagnosis.^4^ Addressing the issues that pertain to MH and general wellness is imperative for the long‐term wellbeing of these patients. One common and effective intervention utilized in the treatment of MH and general wellness is cognitive behavioral therapy (CBT).^5^

CBT has classically been utilized for the treatment of many MH disorders. Literature has shown that CBT in the general population is effective in the treatment of MH disorders, among them depression and anxiety, as well as improvements in quality of life (QoL).^6^ In a most recent meta‐review of systematic reviews and panoramic meta‐analysis by Fordham et al.^7^ a total of 494 systematic reviews and meta‐analyses were identified, reporting an overall statistically significant and moderate treatment effect of CBT for QoL outcomes as well as for MH outcomes such as depression and/or anxiety. In addition, CBT has been shown to not only adequately treat MH disorders, but to reduce relapse and recurrence as well, a feature that is particularly noteworthy when treating chronic conditions such as those oncology patients face.^8^ In fact, studies have shown that CBT use is effective in cancer patients when treating distress and pain.^9^ Besides, CBT has also been shown to be effective in the context of insomnia,^10^ PTSD,^11^ fatigue,^12^ fear,^13^ anxiety and depression^14^ in cancer patients. However, whether CBT is more effective than standard care in the treatment of combined MH and general wellness in cancer patients remains unanswered and is an investigation point of this project. While these studies have shown CBT is effective in treating MH and general wellness in cancer patients, they do not address which outcome is better benefitted in response to CBT and little is known about other factors that may influence the efficacy of CBT.

Recent work has illustrated the efficacy of CBT on MH in breast cancer patients.^15^, ^16^ While these studies have shed light on the importance of CBT in treating cancer patients with MH disorders, they leave gaps in our understanding of CBT as an adjunct to treatment. Furthermore, there are a limited number of trials included in these recent works, and most were limited to nonmetastatic Stage 3 breast cancer patients, excluding cases with more widespread aggressive disease.^17^ Given all that is left to explore, we believe an updated analysis is warranted.

Herein, we discuss the effect of CBT on MH and QoL and analyze two potential moderators of the efficacy of CBT in patients in these two aspects of care: age of patients and the treatment delivery of CBT. We focused on age and treatment delivery of CBT as potentially significant factors impacting CBT's treatment effect for a couple of reasons. First, studies have reported a differential treatment effect of CBT across the age spectrum. For example, Zhang et al.^18^ systematically reviewed existing psychosocial, behavioral, and supportive interventions for pediatric, adolescent and young adult cancer survivors. Findings of the study suggested that supportive interventions, including CBT, were only effective for pediatric cancer patients but not for adolescents and young adults with cancer. Similarly, Mirosevic et al.^19^ found a significant treatment effect of CBT on cancer patients' survival only among middle‐ to older‐adult patients but not among younger (< 40 years old) patients. Second, with recent advancement in technology, the delivery of CBT has become increasingly diverse with CBT delivery formats ranging from in‐person to interpersonal CBT via technology (e.g., Zoom), or even a mixture of multiple methods. The efficacy of CBT in oncology patients may vary depending on delivery method and setting.^20^, ^21^ Specifically, a systematic review and meta‐analysis of internet‐delivered CBT for depression and anxiety among patients with chronic health conditions revealed overall significant and moderate treatment effect sizes for depression and anxiety, *d* = 0.31 and *d* = 0.45, respectively.^22^ On one hand, these point estimates seemed smaller than in‐person CBT's treatment effect for cancer patients, for example, *d* = 0.57 for QoL or *d* = 1.10 for anxiety.^23^ On the other hand, in a recent meta‐analysis, Carlbring et al.^24^ found that internet‐based CBT for psychiatric and somatic disorders is equally effective as in‐person CBT, indicating the need to further clarify the possible differential treatment effect of CBT for cancer patients' MH and QoL outcomes across different delivery formats. Performing this analysis is critical for understanding how to further provide all‐encompassing, comprehensive treatment of cancer patients by not only addressing the disease, but also the psychosocial complications associated with treatments.

## 2 METHODS

This study followed the Methodological Expectations of Cochrane Intervention Reviews (MECIR) and reported findings in accordance with the Preferred Reporting Items for Systematic Review and Meta‐Analysis (PRISMA). Our team included an interdisciplinary team of medical students, behavioral health therapist, psycho‐oncologist, and research synthesis expert. We followed the protocol pre‐registered at PROSPERO: CRD42020200987 but adopted an updated search date from inception to July 2023.

**FIGURE 1:**
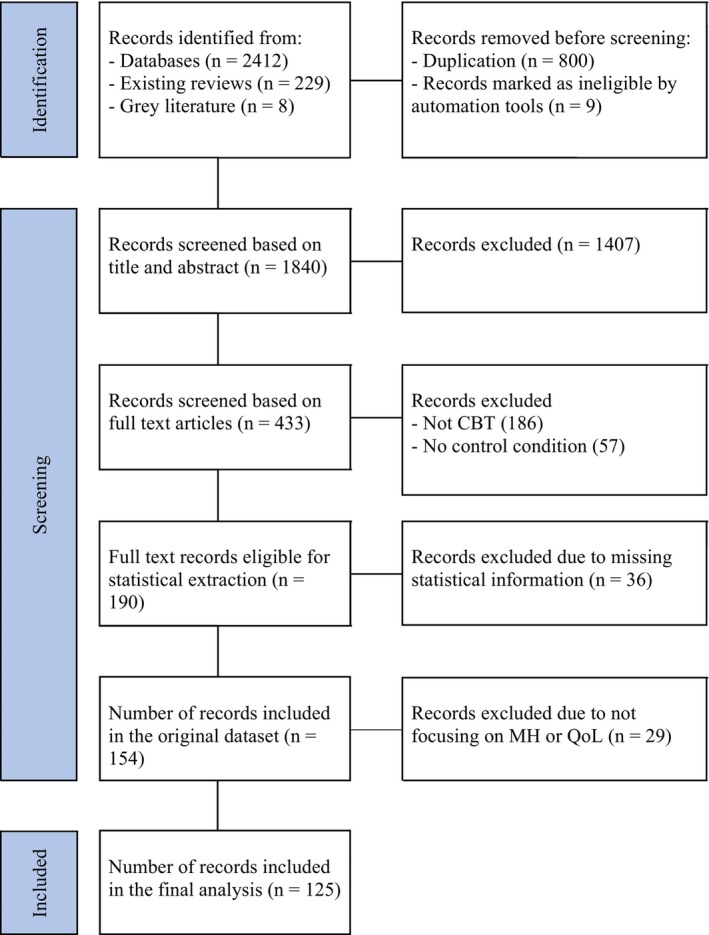
PRISMA 2020 Flow diagram for literature search.

### 2.1 Search procedures and inclusion criteria

We searched all literature from inception to July 2023. We searched for controlled trials, including both randomized and non‐randomized controlled trial studies. We searched across 11 electronic databases, 4 professional websites, plus a manual search of reference lists from relevant published studies. A detailed description of the key search words, specific databases, and others are outline in Data S1. Two research assistants independent screened all articles based on title/abstract, and then full text articles, using the Cochrane recommended systematic review platform, Covidence. Any decision inconsistency between the two independent screeners was first discussed by the two screeners for consensus building, and, if unsuccessful, resolved by a senior scholar on the team to make a final vote. A full record of the literature search is outlined in Figure 1.

### 2.2 Population, intervention, and outcome measures

We focused on the cancer survivor population, which, according to the National Cancer Institute definition, refers to an individual from the time of diagnosis through the balance of life. For intervention consideration, any intervention that was medical or pharmaceutical only were *not* eligible for inclusion. With the focus on CBT, this project considered traditionally‐defined CBT and several important CBT variations. This paper adheres to Beck's CBT definition that CBT is a structured, present‐focused psychotherapeutic approach that is informed by the cognitive behavioral model, and often include the following core components: cognitive restructuring, behavioral activation, problem‐solving skills, and exposure. We considered including third waves of CBT, such as acceptance and commitment‐based CBT or dialectic behavioral therapy. Unfortunately, that would significantly expand the scope of this review and reduce its feasibility. Specifically, by including keywords representing acceptance commitment therapy or dialect behavioral therapy or meta‐cognitive therapy, our initial screening pool expanded from 1840 to 28,191, making the scope too broad to complete given our team's resources available. This decision should be viewed as a limitation of this paper. However, we decided to include mindfulness‐based CBT because mindfulness‐based interventions are very common among cancer survivors and should be included as part of the evidence synthesis. Given the nature and scope of this project, the primary outcomes are cancer survivors' MH and QoL.

### 2.3 Data extraction

The research team developed a data extraction sheet to obtain key information to facilitate data analysis. In addition to bibliographic information, we also extracted study design information (e.g., randomization, type of comparison, sample size), participant characteristics (e.g., average age, %female, %non‐Hispanic White) intervention characteristics (e.g., intervention theory, core CBT component, delivery format, etc.), and other factors (e.g., if supervision was provided or if training was provided). We also extracted necessary statistical information to calculate effect size for meta‐analysis.

### 2.4 Risk of bias assessment

Given the inclusion of both randomized controlled trials and non‐randomized controlled trials, we used the Cochrane Collaboration's Risk of Bias tool 2nd version (RoB 2) for randomized trials, and the Risk of Bias in Non‐randomized Studies – of Interventions (ROBINS‐I) tool for non‐randomized controlled trials. Both the RoB 2 and ROBINS‐I evaluate key aspect of studies using a controlled trial design, for example, bias due to randomization, or bias due to selected outcome reporting.

### 2.5 Data synthesis and statistical analysis

We performed all data analyses using the R Statistical Software. Specifically, we used the meta‐regression analytical framework with robust variance estimation (RVE) to facilitate meta‐analysis. All our outcome measures were standardized mean differences, and we calculated the small sample size corrected Hedges' gas measures of effect size.^25^ We chose the meta‐regression with RVE because it is a method that effectively addresses the issues of dependency among effect sizes from the same study. Additionally, the meta‐regression with RVE method is also an advantageous method because it produces robust estimation of the variance component regardless of the model selection, that is, fixed‐ versus random‐effects models. Therefore, the selection between a fixed‐ versus a random‐effects model is no longer necessary.

In addition to an intercept‐only meta‐regression model to evaluate the overall treatment effect of CBT for cancer survivors' MH and QoL outcomes, we conducted important subgroup analyses and univariate meta‐regression moderator analysis. We considered three important moderators: (1) outcome type, that is, MH versus QoL; (2) age, categorized to <40 years old which represents pediatric, adolescent and young adult cancer survivors, 40–64 years old which represents the middle aged cancer survivors and ≥65 years old which represents older cancer survivors; and (3) delivery format, defined as in‐person, tech‐only interpersonal where CBT delivered person to person virtually, mixed in‐person and tech, pre‐programmed only where CBT was delivered over a programmed software without a person, and tech‐only interpersonal and pre‐programmed. While we also collected other important demographic, clinical, and intervention related variables, we were unable to include many of them due to significant amount of missing to allow meaningful analysis.

**TABLE 1:** Overall treatment effect and subgroup analysis.

	Estimate	N/K	df	95% CI	*p*‐value
Overall	0.388	132/1030	128	0.294–0.483	<0.001
Subgroup analysis with outcome categories					
Mental health outcomes	0.406	119/796	115	0.299–0.512	<0.001
Quality of Life	0.254	63/234	60.9	0.140–0.368	<0.001
Subgroup analysis with age categories					
<40 years old	0.773	8/50	6.92	0.204–1.340	=0.015
40–64 years old	0.384	115/929	112	0.281–0.486	<0.001
≥65 years old	0.092	6/43	4.24	−0.094 – 0.278	=0.245
Subgroup analysis with the delivery format					
In‐person therapy	0.391	77/658	74.5	0.255–0.526	<0.001
Mixed in‐person and tech	0.307	28/203	26.4	0.133–0.481	=0.001
Tech‐only interpersonal	0.323	12/87	10.8	−0.323–0.684	=0.074
Pre‐programmed only	0.483	13/75	11.9	0.225–0.741	=0.002
Tech‐only interpersonal and pre‐programmed	0.991	3/7	1.98	−1.140–3.120	=0.182

**FIGURE 2:**
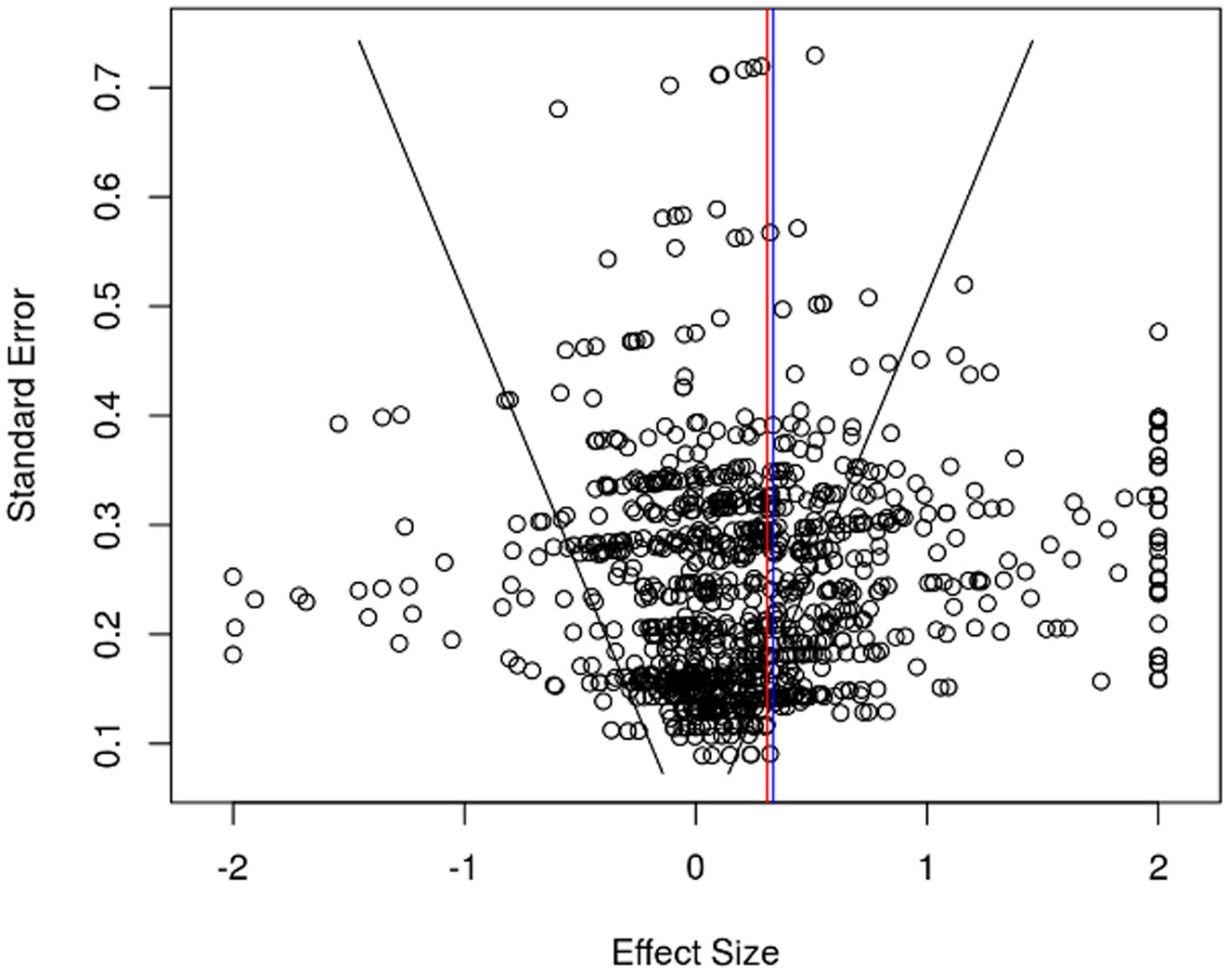
Funnel plot for publication bias.

To evaluate publication bias, plotting of individual effect size estimates against corresponding standard errors was performed using the funnel plot for visual inspection (Figure 2).^26^ Symmetry within the funnel plot is indicative of absent publication bias and in contrast asymmetry is suggestive of publication bias.^27^ In addition, the publication bias was further evaluated with sensitivity analysis using a priori weight functions. This method represents an observed overall treatment effect and a theoretical treatment effect (assuming the funnel plot is symmetrical) and evaluates the difference between the two lines/observations.

## 3 RESULTS

### 3.1 Study characteristics

The present meta‐analysis included 132 trials comprising 1030 effect size estimates and a total sample of 13,226 participants undergoing CBT intervention published between 1986 and 2023. Among the 129 trials reporting participants' age, the average age ranged from 4.2 to 76 years, with a mean of 53.38 years. Regarding participant sex, 78.85% of patients were female (*n* = 10,303) among the 131 trials reporting this demographic. With the exception of 10 trials, all study utilized a randomized controlled trial (RCT) design, while the remaining 10 studies implemented a non‐randomized controlled methodology. In terms of treatment delivery format, 76 studies (57.58%) utilized in‐person intervention, 28 studies (21.21%) employed a combined in‐person and technology‐assisted approach, 13 studies (9.85%) used pre‐programmed technology‐only intervention, 12 studies (9.09%) utilized interpersonal technology‐only intervention, and three studies (2.27%) conducted pre‐programmed and interpersonal technology.

Concerning primary intervention modality, 65 trials (51.59%) utilized individual‐based methods, while 53 studies (42.06%) employed small‐group‐based techniques; additionally, four trials used a family‐based approach, one used couple‐based intervention, and two combined individual and small‐group modalities. Training protocols were implemented in 56% (*n* = 70) of the 125 trails, compared to 56% (*n* = 70) that did not. Among the clinical trials, 53.97% (*n* = 68) incorporated supervision during the intervention, whereas 46.06% (*n* = 58) did not. With respect to comparison groups, 30.30% (*n* = 40) of studies compared the treatment group to treatment‐as‐usual, 21.21% (*n* = 28) to an active control group, and 37.88% (*n* = 50) to a waitlist or attention control group. Looking at patient treatment phase, of the 116 trials documenting this factor, 41.67% (*n* = 55) had patients undergoing current curative treatment, 32.76% (*n* = 38) were in post‐treatment survivorship, 12.93% (*n* = 15) were in a mixed phase, 4.31% (*n* = 5) were newly diagnosed without initiated treatment, one study was in end‐of‐life care, and one focused on palliative treatment (including transition from curative to palliative). Details of individuals studies are presented in Data S2.

### 3.2 Risk of bias assessment

The Revised Cochrane Risk‐of‐Bias tool second version and the Non‐Randomized Studies of the Effects of Interventions (ROBINS‐I) was used in this review to assess the risk of bias for both randomized controlled trials (122/132) and controlled trials without randomization (10/132), respectively. In general, studies included in this review identified a low risk of bias in selection of the reported results (129/132), appropriate reporting in the measurement of the outcome (126/132), deviations from the intended interventions (122/132), and reporting of missing outcome data (103/132). Besides, 10 randomized controlled trials reported moderate risk of bias arising from the randomization process and two non‐randomized controlled trials indicated moderate risk of bias in the selection of participants into the study. In controlled trials without random assignment, one study had a moderate risk of bias due to confounding. Collectively, the included studies revealed a low risk of bias (Data S3).

#### 3.2.1 Overall meta‐analytic and subgroup findings

Across 132 studies inclusive of 1030 effect sizes, CBT reported an overall small to moderate and statistically significant treatment effect size for cancer survivors' MH and QoL outcomes, *d* = 0.388, 95% CI 0.294–0.483, *p* < 0.001 (Table 1). Subgroup analysis across 119 studies and 796 effect sizes revealed an overall moderate and statistically significant treatment effect size of CBT for cancer survivors' MH outcomes, *d* = 0.406, 95% CI 0.299–0.512, *p* < 0.001. Similarly, subgroup analysis across 63 studies and 234 effect sizes reported an overall small and statistically significant treatment effect size of CBT for cancer survivors' QoL outcomes, *d* = 0.254, 95% CI 0.14–0.368, *p* < 0.001.

Subgroup analysis evaluating CBT's treatment effect on MH and QoL outcomes by age group revealed that CBT has an overall large and statistically significant treatment effect size for pediatric and adolescent and young adult cancer survivors (<40 years old), *d* = 0.773, 95% CI 0.204–1.340, *p* = 0.015. Interestingly, while CBT was overall statistically significant for middle‐aged adults (40–64 years old) on their MH and QoL outcomes, *d* = 0.384, 95% CI 0.281–0.486, *p* < 0.001, its treatment effect for older cancer survivors (≥65 years old) was statistically non‐significant, *d* = 0.092, 95% CI −0.094 to 0.278, *p* = 0.245. Subgroup analysis investigating CBT's treatment effect on MH and QoL outcomes by delivery format revealed that CBT has a statistically significant treatment effect size for patients receiving in‐person therapy, *d* = 0.391, 95% CI 0.225–0.526, *p* = <0.001, mixed in‐person and tech, *d* = 0.307, 95% CI 0.133–0.481 *p* = 0.001, and pre‐programmed only, *d* = 0.483, 95% CI 0.225–0.741, *p* = 0.002. Surprisingly, while in‐person and tech were statistically significant, tech‐only interpersonal, *d* = 0.323, 95% CI −0.323 to 0.684, *p* = 0.074 and tech‐only interpersonal and pre‐programmed *d* = 0.991, 95% CI −1.140 to 3.120, *p* = 0.182 were *not* statistically significant.

#### 3.2.2 Moderator analysis findings

Univariate moderator analyses evaluated possible moderators in relation to CBT's treatment effect for cancer survivors (Table 2). Specifically, the difference in treatment effect sizes was statistically *non*‐significant between CBT for cancer survivors' QoL outcomes and MH outcomes, *b* = −0.122, 95% CI −0.286 to 0.041, *p* = 0.140. In addition, cancer survivors' age significantly moderated CBT's treatment effect on MH and QoL outcomes, *b* = −0.011, 95% CI −0.022 to −0.001, *p* = 0.037. For each unit increase in a study's participant average age, that study is expected to report 0.011 smaller treatment effect sizes. The delivery format was not a significant moderator.

**TABLE 2:** Univariate moderator analyses.

	Estimate	N/K	df	95% CI	*p* value
Moderator analysis for mental health and quality of life outcomes combined					
Outcome (ref: mental health)	0.417	132/1030	109.2	0.307–0.526	<0.001
Quality of life	−0.122	132/1030	65.2	−0.286–0.041	=0.140
Age (intercept)	0.983	129/1022	10.9	0.395–1.570	=0.003
Age (slope)	−0.011	129/1022	12.0	−0.022–0.001	=0.037
Delivery format (ref: In‐person)	0.3891	132/1030	74.02	0.255–0.524	<0.001
Mixed in‐person and tech	−0.069	132/1030	48.62	−0.290–0.151	=0.530
Tech‐only interpersonal	−0.058	132/1030	14.81	−0.438–0.322	=0.750
Pre‐programmed only	0.094	132/1030	16.28	−0.195–0.383	=0.500
Tech‐only interpersonal and pre‐programmed	0.591	132/1030	2.06	−1.591–2.773	=0.371

## 4 DISCUSSION

CBT is a research‐supported treatment for individuals living with a cancer diagnosis across a broad spectrum of outcomes, with the strongest support for its effect for MH outcomes.^28^ Given the established relationship between MH and QoL,^29^ it is reasonable to expect that CBT would benefit cancer survivors' QoL life outcomes. To that end, the purpose of this study is to empirically evaluate the treatment effect of CBT for MH and QoL among individuals with cancer.

Consistent with the existing literature, when combining CBT's treatment effect for both MH and QoL outcomes, an overall statistically significant treatment effect was identified.^23^, ^30^ Importantly, however, subgroup analysis suggested a moderate versus small overall treatment effect of CBT for MH and QoL outcomes, respectively. While CBT's overall treatment effect was statistically significant for both outcome domains, findings of this study suggested a relatively greater overall treatment effect size of CBT for MH (moderate) versus QoL (small). It is important to note that moderator analysis revealed the difference in the effect size for MH versus QoL outcomes was statistically non‐significant. This particular finding renders several important clinical implications. First, oncological providers should continue to view CBT as a research‐supported intervention for both MH and QoL outcomes. Second, individuals with cancer may benefit more from CBT for their MH outcomes than QoL outcomes. Third, researchers and oncological providers are encouraged to further consider how to maximize the benefit of CBT for MH to have a stronger connection with cancer patients/survivors QoL outcomes. For example, existing literature have suggested that MH combined with improvement in social relationship and self‐efficacy will lead to improvement in cancer patients/survivors QoL.^30^, ^31^

In addition to our main meta‐analytical findings, our selection of age and delivery format for subgroup and moderator analysis revealed a couple of clinically significant findings. First, subgroup analysis of CBT's treatment effect across cancer patients/survivors age spectrum revealed CBT being overall statistically significant for pediatric, adolescent and young adult cancer patients/survivors (<40 years old), middle adults with cancer (40–64 years old), but *ineffective* for older cancer patients/survivors (>65 years old). This reveals a significant gap in the psychosocial oncology literature of CBT for MH and QoL outcomes. Given the majority of individuals diagnosed with cancer are those who are of older age,^32^ an overall statistically non‐significant treatment of CBT for this age group's MH and QoL outcome is concerning and warrant further investigation. Notably, this finding seemed consistent with the existing CBT literature that has found older adults benefiting less than their middle adult counterpart from receiving CBT.^33^, ^34^

Second, in addition to age, we have evaluated treatment delivery as a factor for subgroup and moderator analysis. Notably, subgroup analysis revealed that the overall treatment effects of CBT were statistically non‐significant when being delivered as an interpersonal modality only via technology, that is, having a human therapist/clinician delivering care via tech platforms, or technology‐only intervention combined with pre‐programmed content. This is an interesting finding as it seemed to suggest that when there is an interpersonal component involved in the treatment delivery, an in‐person modality seemed to be critical to ensure treatment effectiveness. One possible explanation is that, for patients choose to receive interpersonal therapy, they are expect some level of human‐to‐human in‐person connection, that can be difficult to be replaced any technological format, for example, having a live human therapist on the other end of Zoom. Future investigations should focus on how delivery format impacts interpersonal supportive care for cancer patients/survivors. It was, however, encouraging to reveal that other formats of delivering CBT have been equally beneficial for individuals living with a cancer diagnosis, suggesting CBT's dexterity for large‐scale uptake.

An important context to understanding the study's findings is the proportion of studies with a relatively small sample size. Due to the state of the science/published literature, many studies included in this meta‐analysis were considered “preliminary efficacy trials.” To a certain extent, this feature of the existing literature reflected a lack of high‐quality clinical trials that are well conceptualized and have a large sample size of CBT for individuals with cancer. Despite the relatively well‐established clinical efficacy of CBT for individuals with cancer, future studies of CBT in cancer should prioritize CBT intervention fidelity, maturity, and large sample size.

Overall, our findings indicate that CBT is associated with marked improvement in MH and QoL for all types of cancer patients including those actively in treatment and post treatment. A notable implication of this study's findings suggests that CBT seemed to have benefits for cancer patients/survivors beyond MH outcomes but also their QoL. The broad scope of CBT benefits suggests that CBT being made available to cancer patients/survivors, even when a formal MH diagnosis is not imminently present, could be warranted. Further, our findings support the need for further investigation into psychosocial treatment modalities that may better serve those in the older adult population. These findings are informative to clinical practice and how best to treat the psychosocial realm of cancer treatment.

## 5 STRENGTHS AND LIMITATIONS

The findings of this project should be contextualized in some limitations of the study. First, there is always a chance that we may have missed some articles despite a comprehensive search strategy we have followed. Such a concern, however, is relatively low given the large number of articles and very large number of effect sizes included in the analysis. Second, it is possible that we may have missed other important factors for subgroup and moderator analyses. However, multiple statistical tests would result in an inflated Type I error rate, and therefore, we selected two theoretically and clinically meaningful factors to consider in this paper. Finally, for this study's moderator analysis, we used univariate moderator analysis, that is to enter the moderators in meta‐regression once a time. Given meta‐regression uses case wise deletion for missing value, that is, if missing in one variable, the entire case is deleted, we chose to use univariate meta‐regression to reduce the impact of missingness, though multi‐variable meta‐regression may offer a more nuanced understanding of CBT's treatment effect. This should be the focus of future investigations when more data (with minimal missing) becomes available.

Notwithstanding the above‐mentioned limitations, this study represents one of the most comprehensive and largest systematic review and meta‐analysis study that evaluated CBT's treatment effect for cancer patients, and one of the first that included both MH and QoL outcomes. This study benefited from a large sample size with an advanced statistical analysis framework that facilitates rigorous and flexible modeling needs. Finally, findings of the study provides important clinical implications, especially the focus on CBT's differential treatment effect across age groups and delivery format among cancer patients/survivors.

## 6 CONCLUSION

Based on previous literature, cancer patients suffer from impaired MH and QoL, and integration of CBT into treatment regiments has been a useful tool in assisting with these psychosocial challenges. In this study, we noted a mild to moderate improvement in MH and QoL in cancer patients receiving CBT as a form of treatment. Additionally, we found that age and treatment delivery setting can influence the efficacy of CBT in cancer patients. These results have implications in effectively treating the psychosocial complications associated with cancer treatment. Further work will need to be performed investigating other factors involved in CBT treatment in cancer patients.

## AUTHOR CONTRIBUTIONS

**Alexander T. Dils:** Formal analysis (equal); investigation (equal); project administration (equal); supervision (equal); writing – original draft (equal); writing – review and editing (equal). **Kathryn O'Keefe:** Data curation (equal); investigation (equal); methodology (equal); writing – original draft (equal); writing – review and editing (equal). **Nada Dakka:** Data curation (equal); methodology (equal); validation (equal); writing – review and editing (equal). **Michelle Azar:** Data curation (equal); methodology (equal); validation (equal); writing – original draft (equal); writing – review and editing (equal). **Meiyan Chen:** Data curation (equal); formal analysis (equal); writing – review and editing (equal). **Anao Zhang:** Conceptualization (equal); data curation (equal); formal analysis (equal); investigation (equal); methodology (equal); supervision (equal); writing – original draft (equal); writing – review and editing (equal).

## FUNDING INFORMATION

No funding to report.

## CONFLICT OF INTEREST STATEMENT

All authors of this paper have no conflict of interest to disclose.

## ETHICS STATEMENT

Given the nature of this paper, ethics approval is not relevant.

## Supporting information


Data S1.

Data S2.

Data S3.

Data S4.

## Data Availability

Data can be made available upon reasonable request to the corresponding author.
