# Supplementary material for: The efficacy of cognitive behavioral therapy for mental health and quality of life among individuals diagnosed with cancer: A systematic review and meta‐analysis
Source: Cancer Med. 2024 Aug 21;13(16):e70063. doi: 10.1002/cam4.70063 (PMC11336377; doi:10.1002/cam4.70063)
Supplement: Supplementary file 1 — Data S1. [file CAM4-13-e70063-s002.docx]

| **Supplement 3. Risk of Bias Assessment** | | | | | | |
| --- | --- | --- | --- | --- | --- | --- |
| Risk of Bias | | | | | | |
|  | Randomization^1^ | Deviation^2^ | Missingness^3^ | Measurement^4^ | Reporting^5^ | Overall^6^ |
| Abrahams et al., 2017 | A | A | A | A | A | A |
| Agyemang et al., 2016 | A | A | A | A | A | A |
| Allen et al., 2002 | A | A | B | A | A | A |
| Ames et al., 2011 | A | A | A | A | A | A |
| Armes et al., 2007 | A | A | A | A | A | A |
| Arving et al., 2007 | A | A | A | A | A | A |
| Aubin et al., 2019 | A | A | A | A | A | A |
| Baucom et al., 2009 | A | A | B | A | A | A |
| Butow et al., 2017 | A | A | A | A | A | A |
| Capezzani et al., 2013 | A | A | B | A | A | A |
| Carpenter et al., 2014 | A | A | B | A | A | A |
| Casault et al., 2015 | A | A | B | A | A | A |
| Chan et al., 2017 | A | A | B | A | A | A |
| Cohen et al., 2007 | A | A | A | A | A | A |
| Compen et al., 2018 | A | A | A | A | A | A |
| Dolbeault et al., 2009 | A | A | A | A | A | A |
| Edelman et al., 1999 | A | A | B | A | A | A |
| Edelman et al., 1999 | A | A | B | A | A | A |
| Edmonds et al., 1999 | A | A | B | A | A | A |
| Ferguson et al., 2016 | A | A | B | A | A | A |
| Garland et al., 2014 | A | A | A | A | A | A |
| Germino et al., 2012 | A | A | A | A | A | A |
| Gielissen et al., 2006 | A | A | A | A | A | A |
| Goode et al., 2011 | A | A | B | A | A | A |
| Gregoire et al., 2017 | A | A | B | A | A | A |
| Gudenkauf et al., 2015 | A | A | B | A | A | A |
| Hummel et al., 2017 | A | A | A | A | A | A |
| Irwin et al., 2017 | A | A | A | A | A | A |
| Johannsen et al., 2016 | A | A | A | A | B | A |
| Johannsen et al., 2018 | A | A | A | A | A | A |
| Johansson et al., 2008 | A | A | A | A | A | A |
| Kangas et al., 2013 | A | A | A | A | A | A |
| Kazak et al., 2004 | A | A | A | A | A | A |
| Lau et al., 2020 | A | A | A | A | A | A |
| Lee et al., 2011 | A | A | B | A | A | A |
| Locke et al., 2008 | A | A | B | A | A | A |
| Mann et al., 2012 | A | A | A | A | A | A |
| Matthews et al., 2014 | A | A | A | A | A | A |
| May et al., 2009 | A | A | A | A | A | A |
| Mendoza et al., 2017 | A | A | B | A | A | A |
| Mishel et al., 2002 | A | A | B | A | A | A |
| Moon et al., 2020 | A | A | A | A | A | A |
| Mosher et al., 2016 | A | A | A | A | A | A |
| Nezu et al., 2003 | A | A | B | A | A | A |
| Nissen et al., 2020 | A | A | A | A | A | A |
| Norouzi et al., 2017 | A | A | B | A | A | A |
| Park et al., 2020 | A | A | A | A | A | A |
| Penedo et al., 2020 | A | A | A | A | A | A |
| Qiu et al., 2013 | A | A | A | A | A | A |
| Ren et al., 2019 | A | A | A | A | A | A |
| Ritterband et al., 2012 | A | A | A | A | A | A |
| Rogers et al., 2017 | A | A | A | A | A | A |
| Sandgren et al., 2000 | A | A | B | A | B | A |
| Savard et al., 2005 | A | A | A | A | A | A |
| Savard et al., 2006 | A | A | B | A | A | A |
| Stefanopoulou et al., 2015 | A | A | A | A | A | A |
| Strong et al., 2008 | A | A | A | A | A | A |
| Syrjala et al., 2018 | A | A | B | A | B | A |
| van de Wal et al., 2017 | A | A | A | A | A | A |
| van den Berg et al., 2015 | A | A | A | A | A | A |
| van der Lee et al., 2012 | A | A | B | A | A | A |
| van der Meulen et al., 2013 | A | A | A | A | A | A |
| van Weert et al., 2010 | A | A | B | A | A | A |
| Wells-Di Gregorio et al., 2019 | A | A | A | A | A | A |
| Abedini er al, 2021 | A | A | A | A | A | A |
| Acevedo-Ibarra er al, 2019 | A | A | A | A | A | A |
| Antoni et al, 2006 | B | A | A | A | A | A |
| Antoni et al, 2001 | A | A | A | A | A | A |
| Beatty et al, 2010 | A | A | A | A | A | A |
| Beatty et al, 2016 | A | A | A | A | A | A |
| Betiar et al, 2021 | A | A | A | A | A | A |
| Bottomley et al, 1996 | B | A | B | A | A | A |
| Chambers et al, 2017 | A | A | A | A | A | A |
| Chambers et al, 2014 | A | A | A | A | A | A |
| Chambers et al, 2018 | A | A | B | A | A | A |
| Cousson-Gealie et al, 2011 | A | A | A | A | A | A |
| David et al, 2013 | A | A | A | A | A | A |
| Davis et al, 1986 | B | A | A | B | A | A |
| Desautels et al, 2018 | A | A | A | A | A | A |
| Diaz et al, 2021 | A | A | A | A | A | A |
| do Camo et al, 2017 | A | B | A | A | A | A |
| Downe-Wamboldt et al, 2007 | A | A | A | A | A | A |
| DuHamel et al, 2010 | A | A | A | A | A | A |
| Evans et al, 1995 | A | B | A | A | A | A |
| Fadeal et al, 2011 | B | A | A | A | B | A |
| Ferguson et al, 2012 | A | A | A | A | A | A |
| Foley et al, 2010 | B | B | A | A | A | A |
| Garland et al, 2015 | A | A | A | A | A | A |
| Gaston-Johansson et al, 2013 | A | B | A | A | A | A |
| Graboyes et al, 2022 | A | A | A | A | A | A |
| Greer et al, 2012 | A | B | A | A | A | A |
| Groarke et al, 2013 | A | A | A | A | A | A |
| Ham et al, 2019 | A | A | A | A | A | A |
| Heinrichs et al,2012 | A | A | A | A | A | A |
| Herschbach et al, 2010 | A | A | A | A | A | A |
| Hyland et al, 2022 | A | A | A | B | A | A |
| Kingston et al, 2015 | A | A | A | A | A | A |
| Kissane et al, 2003 | A | A | A | A | A | A |
| Klosky et al, 2004 | A | A | A | B | A | A |
| Korstjens e al,2011 | A | A | A | A | A | A |
| Lechner et al, 2014 | A | A | A | A | A | A |
| Mann et al, 2012 | B | A | A | A | A | A |
| Manne et al, 2007 | A | A | A | A | A | A |
| Manne et al, 2017 | A | A | A | A | A | A |
| Marchioro et al,1996 | A | A | A | A | A | A |
| Merckaert et al, 2017 | A | A | A | A | A | A |
| Mishel et al, 2005 | A | A | A | A | A | A |
| Mokrivala et al, 2022 | A | A | A | A | A | A |
| Moorey et al, 1998 | B | B | A | A | A | A |
| Murphy et al, 2020 | A | B | A | A | A | A |
| Onyechi et al, 2016 | A | A | A | A | A | A |
| Peoles et al, 2017 | B | A | B | A | A | A |
| Phillips et al, 2011 | A | A | A | A | A | A |
| Richardson et al, 2017 | A | A | A | A | A | A |
| Serfaty et al, 2000 | A | A | A | A | A | A |
| Serfaty et al, 2012 | A | B | A | A | A | A |
| Solkoglu et al, 2023 | A | A | A | A | A | A |
| Speer, 1987 | B | A | A | A | A | A |
| Stagel et al, 2015 | A | B | A | A | A | A |
| Taeidi et al, 2018 | A | A | A | A | A | A |
| Trask et al, 2003 | A | B | A | A | A | A |
| Tyc et al, 1997 | B | A | A | A | A | A |
| Wells-Di et al, 2019 | A | A | A | A | A | A |
| Yanez et al, 2015 | A | A | A | A | A | A |
| Yang et al, 2022 | A | A | A | A | A | A |
| Zhang et al, 2023 | A | A | A | A | A | A |
| * A = Low risk of bias (green color); C = High risk of bias (red color); B = Some concerns (Mid-level risk of bias, yellow color)  1. Risk of bias arising from the randomization process  2. Risk of bias due to deviations from the intended interventions (effect of assignment to intervention)  3. Missing outcome data  4. Risk of bias in measurement of the outcome  5. Risk of bias in selection of the reported results  6. Overall risk of bias | | | | | | |

| **Supplement 3. Risk of Bias Assessment** | | | | | | | | |
| --- | --- | --- | --- | --- | --- | --- | --- | --- |
| ROBINS-I | | | | | | | | |
|  | Confounding^1^ | Participant^2^ | Classification^3^ | Deviation^4^ | Missingness^5^ | Measurement^6^ | Reporting^7^ | Overall^8^ |
| Bragard et al., 2017 | A | A | A | A | A | A | B | A |
| Cole et al., 1999 | B | B | A | A | A | A | B | A |
| Bottomley et al, 1996 | A | A | A | A | A | A | A | A |
| Chen et al, 2014 | A | B | A | A | A | A | A | A |
| McKierman et al, 2010 | A | A | A | A | A | A | A | A |
| Poggi et al, 2009 | A | A | A | A | A | A | A | A |
| Wojtyna et al, 2007 | A | A | A | A | B | A | A | A |
| Zhang et al., 2019 | A | A | A | A | B | A | A | A |
| * A = Low risk of bias (green color); C = High risk of bias (red color); B = Some concerns (Mid-level risk of bias, yellow color)  1. Bias due to confounding  2. Bias in selection of participants into the study  3. Bias in classification of interventions  4. Bias due to deviations from intended interventions  5. Bias due to missing data  6. Bias in measurement of outcomes  7. Bias in selection of the reported result  8. Overall bias | | | | | | | | |
