# Supplementary material for: The efficacy of cognitive behavioral therapy for mental health and quality of life among individuals diagnosed with cancer: A systematic review and meta‐analysis
Source: Cancer Med. 2024 Aug 21;13(16):e70063. doi: 10.1002/cam4.70063 (PMC11336377; doi:10.1002/cam4.70063)
Supplement: Supplementary file 2 — Data S2. [file CAM4-13-e70063-s004.docx]

| **Supplemental Table 2. Study characteristics of included studies** | | | | | | | | |
| --- | --- | --- | --- | --- | --- | --- | --- | --- |
| Author | Sample | Demographics | Control | Diagnosis | Treatment Stage | Intervention Description | Outcome | Measure |
| Abrahams et al., 2017 | T = 66  C = 66 | Age = 51.5  %Female = 100%  %White = NR | Treatment as usual or standard care | Breast cancer diagnosis | Post-treatment survivorship | Internet-Based Cognitive Behavioral Therapy (iCBT)  ***Intervention Characteristics:*** The format of iCBT consisted of three face-to-face individual sessions and a maximum of eight web-based modules, with a duration of six months for iCBT. The content of the intervention was developed from an evidence-based, face-to-face CBT protocol for severely fatigued cancer survivors with mixed cancer diagnosis. iCBT is based on a cognitive behavioral model of precipitating and perpetuating factors of fatigue, in which it is assumed that the malignancy and its treatment induce the fatigue whereas cognitive behavioral factors maintain the fatigue. iCBT was delivered online primarily but included therapists’ support.  ***Provider:*** Licensed cognitive behavioral therapists provided the electronic consultations. | Fatigue;  Functional impairment;  Psychological distress;  QoL. | CIS-fatigue  SIP-8  BSI-18  EORTC QOL-C30 |
| Agyemang et al., 2016 | T = 16  C = 15 | Age = 47.0  %Female = 79%  %White = 64% | Treatment as usual or standard care | Multiple cancer diagnoses | Initiating chemotherapy and/or radiation for the first time | Sleep Healthy Using the Internet (SHUTi)  ***Intervention Characteristics:*** SHUTi was formatted to include six, weekly treatment cores modeled after weekly face-to-face CBT-I sessions. Each core took approximately 45 to 60 minutes to complete. The content of SHUTi was organized in a serial manner. The first session provided a review and rationale of insomnia and its treatment. Second is the sleep behavior core, which introduced an implemented sleep restriction as well as stimulus control. The next core is sleep behavior core two and built on the previous core by addressing concerns about sleep restriction while expanding on stimulus control instructions. The fourth core is the sleep education core, which focuses on teaching sleep hygiene. The fifth core is the sleep thoughts core, aptly named for its focus on cognitive restructuring. Last is the problem prevention core, which sought to synthesize information from the previous five cores. No information on delivery was provided.  ***Provider:*** No information on the characteristics of the intervention provider(s) was available. | Insomnia;  Depression;  Anxiety;  Functional health. | ISI  PHQ-9  GAD-7  FACT-G |
| Allen et al., 2002 | T = 87  C = 77 | Age = 42.3  %Female = 100%  %White = 82.9% | Waitlist or attention control | Breast carcinoma diagnosis | Participants were beginning their first course of chemotherapy | Problem-Solving Approach to Stress Reduction  ***Intervention Characteristics:*** The Problem-Solving Approach to Stress Reduction was formatted to facilitate individual sessions on six occasions, with the first session approximately two hours long, plus four follow-up telephone sessions two weeks apart, and a final in-person two-hour session. Content was structured around five problem-solving skill training sessions: (1) problem orientation; (2) problem definition; (3) generation of alternatives; (4) decision-making; and (5) solution implementation. The intervention was delivered to participants by a combination of in-person and telephone.  ***Provider:*** The Problem-Solving Approach to Stress Reduction was provided by an oncology research nurse. | QoL;  Mental health;  Distress. | CARES  MHI  IES |
| Ames et al., 2011 | T = 27  C = 30 | Age = 76 median  %Female = 0%  %White = 89% | Waitlist or attention control | Prostate cancer diagnosis | Active on-going treatment | Multidisciplinary Quality of Life Intervention  ***Intervention Characteristics:*** The format of the Multidisciplinary Quality of Life Intervention consisted of eight, one-hour, structured treatment sessions in an in-person group setting. The content of each session in the Multidisciplinary Quality of Life Intervention followed the subsequent order: (1) program overview and medical education regarding prostate cancer; (2) goal setting, problem solving, and relaxation training; (3) nutrition and prostate cancer; (4) physical activity and conditioning; (5-7) mood management; and (8) social support and maintenance of positive health behavior change.  ***Provider:*** The Multidisciplinary Quality of Life Intervention was provided by a clinical health psychologist. | QoL;  Function;  Anxiety;  Mood;  Stress. | SF-36  FACT  MAX-PC  POMS  PSS |
| Armes et al., 2007 | T = 27  C = 27 | Age = 50.25  %Female = 100%  %White = 68% | Treatment as usual or standard care | Multiple cancer diagnoses | Various treatment stages | Brief Behavioral Oriented Intervention (BBOI)  ***Intervention Characteristics:*** The format of BBOI consisted of three individual, face-to-face, sixty-minute sessions that were delivered in-person at three-to-four-week intervals (coinciding with the administration of chemotherapy). BBOI had a treatment manual with audiotaped sessions for supervision. The content of BBOI was centered around goal setting, psychoeducation, cognitive restructuring, behavioral activation, and problem solving.  ***Provider:*** The intervention was provided by two therapists not further described. | Fatigue;  QoL. | VAS global fatigue  EORTC QLQ-C30 |
| Arving et al., 2007 | T_1_ = 60  T_2_ = 60  C = 59 | Age = 55.0  %Female = 100%  %White = NR | Treatment as usual or standard care | Breast cancer diagnosis | Post-treatment survivorship | Individual Psychosocial Support (INS/IPS)  ***Intervention Characteristics:*** The format of INS/IPS consisted of individual sessions delivered in-person, with the number of sessions defined by participants, and each session scheduled to last 45 to 60 minutes. The core strategies of INS/IPS content include problem solving, relaxation and distraction techniques, ways to improve communication, and activity scheduling to manage symptoms.  ***Provider:*** INS/IPS was provided by two oncology nurses trained in psychosocial support. | QoL;  Symptom severity. | EORTC QLQ  IES |
| Aubin et al., 2019 | T = 56  C = 52 | Age = 30.14  %Female = 76%  %White = 84% | Treatment as usual or standard care | Multiple cancer diagnoses | Post-treatment survivorship | Intervention  ***Intervention Characteristics:*** The format of the intervention consisted of three individual sixty-minute sessions delivered every other week. In addition, participants were given the option to receive the intervention by either face to face or through Skype delivery. The main objective of the intervention was to provide participants with a “toolbox” of coping strategies and resources to proactively manage stressful situations in three areas: (1) social/family relationships; (2) couple/sexual relationships; and (3) challenges related to cancer.  ***Provider:*** The intervention was provided by a Ph.D. clinical psychologist. | Depression;  Anxiety;  Distress;  Functional health;  Self-Efficacy;  Sexuality. | HADS  FACT-G  CDSES  SC |
| Baucom et al., 2009 | T = 4  C = 4 | Age = (median) 50  %Female = 100%  %White = 86% | Treatment as usual or standard care | Breast cancer diagnosis | Participants receiving on-going treatment | Couple-Based Relationship Enhancement (CBRE)  ***Intervention Characteristics:*** The format of CBRE consisted of six couple therapy session, delivered face-to-face in-person on a bi-weekly basis, at seventy-five minutes per session. The content of CBRE centered on psychoeducation, problem-solving, emotional expressiveness skills, meaning making, and cognitive restructuring.  ***Provider:*** CBRE was provided by advanced doctoral students in clinical psychology. | Relationship quality;  Sexual function;  Posttraumatic growth;  Psychological distress;  Functional health;  Fatigue;  Pain;  Symptom severity. | QMI  DISF  BSI  PGI  FACT  BFI  RSI |
| Bragard et al., 2017 | T = 10  C_1_ = 21  C_2_ = 68 | Age = 54.13  %Female = 100%  %White = NR | Active comparator | Breast cancer diagnosis | Multiple stages of treatment | Cognitive-Behavioral Therapy (CBT)  ***Intervention Characteristics:*** The format of CBT consisted of six weekly ninety-minute sessions in groups of three to eight participants, which were delivered in-person. CBT was modeled on previous studies. The intervention’s content centered on: (1) breast cancer, meaning of illness, understanding stress, and responses to it; (2) impact of treatment on body image; (3) impact of treatment on self-esteem; (4) fear of recurrence; (5) relationship with relatives and health professionals; and (6) life projects, return to daily activities, and work.  ***Provider:*** The intervention was provided by CBT-trained psychologists with experience in psycho-oncology. | Depression;  Anxiety;  QoL;  Functional health. | HADS  EORTC QLCQ 30 |
| Butow et al., 2017 | T = 121  C = 101 | Age = 52.82  %Female = 95%  %White = NR | Waitlist or attention control | Breast or colorectal or melanoma diagnoses | Post-treatment survivorship | ConquerFear  ***Intervention Characteristics:*** The format of ConquerFear consisted of individual treatment, intensity, frequency, and duration NR. ConquerFear was delivered in-person. The content of ConquerFear was theoretically based, focusing on a manualized intervention influenced by the Common-Sense Model of illness, the Self-Regulatory Executive Function model, and Relational Frame Theory. The intervention centered around teaching strategies for controlling worry and excessive threat monitoring, modify unhelpful beliefs about worries, develop appropriate monitoring and screening behaviors, educate about follow-up and strategies to reduce risk of recurrence, address existential issues, and promote goal setting.  ***Provider:*** The intervention was provided by therapists. | Fear of cancer recurrence;  Distress;  QoL. | FCRI  DASS  AQLF |
| Capezzani et al., 2013 | T = 10  C = 11 | Age = 52.70  %Female = 91%  %White = NR | Active comparator | Multiple cancer diagnoses | Participants receiving on-going treatment | Cognitive-Behavioral Therapy (CBT)  ***Intervention Characteristics:*** The format of CBT consisted of an individually focused approach, with delivery occurring in-person. The content of CBT centered around efforts to stabilize initial symptoms, and integrate strategies such as rational emotive imagery, psychoeducation and homework, address flashback and intrusive thoughts, cognitive restructuring.  ***Provider:*** No information on the provider(s) of CBT here was available. | Psychophysiology;  Anxiety;  Distress;  Traumatic Event; | QPF-R  STAI  BDI-II  CAPS |
| Carpenter et al., 2014 | T = 71  C = 61 | Age = 50.90  %Female = 100%  %White = NR | Waitlist or attention control | Breast cancer diagnosis | Participants receiving active treatment | Coping with Cancer Workbook (CCW)  ***Intervention Characteristics:*** The format of CCW consisted of six weekly ninety-minute sessions in groups of three to eight participants. CCW was delivered primarily through the internet, with clinicians facilitating discussion posts. The content of CCW focused on providing an introduction and 10 chapters of content (including didactic instruction) relating to cognitive and behavioral coping strategies and supporting interactive exercises; relaxation training, including guided imagery and meditation techniques; guided expressive writing exercise; and weekly homework activities to promote integration of new coping skills into daily life.  ***Provider:*** CCW was a self-help program delivered through the internet. | Self-efficacy;  Benefit finding;  Negative mood;  Distress;  Functional wellness;  Positive affect. | CBT  BFS  NMR  IES  SWB  FWB  PAFF |
| Casault et al., 2015 | T = 20  C = 18 | Age = 56.9  %Female = 92.1%  %White = NR | Waitlist or attention control | Multiple cancer diagnoses | Participants were receiving a mixture of on-going treatment and post-treatment survivor | Minimal Cognitive-Behavioral Therapy Insomnia (Minimal CBT-I)  ***Intervention Characteristics:*** The format of Minimal CBT-I consisted of self-help CBT offered in a bibliotherapy format, combined with two brief phone consultations. Phone consultations were at maximum thirty mins each, with an average twenty-two-minute duration, once every two weeks. Minimal CBT-I was delivered primarily in-person, with phone consultation available. The content of Minimal CBT-I focused on a multimodal approach using combined behavioural (i.e., stimulus control therapy, sleep restriction), cognitive (i.e., cognitive restructuring), and educational (i.e., sleep hygiene) strategies that were explained in the patients’ booklets.  ***Provider:*** The intervention was provided via phone consultations delivered by a psychologist every two weeks. | Insomnia;  Anxiety;  Depression;  QoL. | ISI  HADS anxiety  HADS depression  MFI  EORTC QOL  SBQ |
| Chan et al., 2017 | T = 34  C = 38 | Age = 53  %Female = 100%  %White = 0% | Treatment as usual or standard care | Breast cancer diagnosis | Participants were in early stage receiving on-going treatment | Multidisciplinary Psychoeducation Group Intervention (PEG)  ***Intervention Characteristics:*** The format of PEG consisted of participants attending three in-person educational sessions of PEG, with each session being four-and-one-half hours in duration. Each patient participated in three sessions and a group approach was utilized in each session. The content of PEG was conducted based on the principles of cognitive behavioral therapy, utilizing cognitive restructuring, behavioral activation, and problem-solving.  ***Provider:*** PEG was facilitated by healthcare professionals from various specialties who shared their knowledge and experiences. | Symptom distress;  QoL. | RSCL  EORTC QLQ-C30 |
| Cohen et al., 2007 | T = 38  C_1_ = 39  C_2_ = 37 | Age = 53.52  %Female = 100%  %White = NR | Active comparator  Treatment as usual or standard care | Breast cancer diagnosis | Post-treatment survivorship | Cognitive Behavioral Group Intervention (CBGI)  ***Intervention Characteristics:*** The format of CBGI was based on Beck’s model. CBGI was delivered in-person. The content of each session included cognitive and behavioral strategies. The cognitive component focused on learning to elicit negative thinking patterns, learning to identify and monitor automatic thoughts and beliefs and restructure them into more adaptive patterns, and finding alternative stress-reducing thoughts. Additional components include mental distraction, reframing, problem-solving, and decision-making strategies were taught. The behavioral component focused on activity scheduling, graded task assignment, behavioral distraction, and behavioral experiment techniques.  ***Provider:*** CBGI was provided by therapists, who were described no further. | General stress;  Sleep;  Fatigue;  Health locus of control. | GSI  FSI  PSS  MSQ  MHLC |
| Cole et al., 1999 | T_1_ = 14  T_2_ = 12 C = 16 | Age = 54  %Female = 74%  %White = 100% | Active comparator  Waitlist or attention control | Multiple cancer diagnoses | Participants were receiving on-going treatment | Internet-based Cognitive Behavioral Program (I-CBT)  ***Intervention Characteristics:*** The format of I-CBT consisted of manualized and small group CBT sessions with an average of six sessions. Each session of the intervention was delivered in-person. The content of I-CBT focused on building rapport, cognitive restructuring, behavioral activation, and problem solving.  ***Provider:*** I-CBT was provided by therapists that were licensed clinicians in social work or counseling psychology, and one was an advanced doctoral student in clinical psychology. All providers received eight to ten hours of training on the treatment manual and received weekly supervision. | Psychological distress;  Distress;  Pain. | BSI  IES  PS |
| Compen et al., 2018 | T_1_ = 77  T_2_ = 90 C = 78 | Age = 51.7  %Female = 85.7%  %White = NR | Treatment as usual or standard care | Multiple cancer diagnoses | Participants were in both active treatment and post-treatment survivorship | Face-to-face Mindfulness-Based Cognitive Therapy (MBCT)  ***Intervention Characteristics:*** The format of MBCT consisted of eight weekly two-and-one-half-hour group sessions, a six-hour silent day, and daily home practice assignment guided by audio files. MBCT was delivered in-person, with home practice guided by audio files. The content of MBCT and its provision followed the manual/protocol of published MBCT protocol by Segal and colleagues (2013).  ***Provider:*** The intervention was provided by a UK mindfulness-based teacher therapist network good practice guidelines certified clinician.  Internet-Based Mindfulness-Based Cognitive Therapy (eMBCT)  ***Intervention Characteristics:*** The format of eMBCT consisted of individual sessions including weekly asynchronous written interaction with a therapist over email. Web material for eight weeks plus a silent day and an inbox. eMBCT was delivered in person, with home practice guided by audio files. Each session of eMBCT included an introduction and daily meditation exercise with meditation audio files.  ***Provider:*** The intervention was provided by a UK mindfulness-based teacher therapist network good practice guidelines certified clinician. | Psychological distress;  Fear of cancer recurrence;  Mental health status. | HADS  FCRI  RRQ-rumination  SF-12 mental  SF-12 physical  FFMQ-SF  MHC-SF |
| Dolbeault et al., 2009 | T = 102  C = 101 | Age = 53.06  %Female = 100%  %White = NR | Waitlist or attention control | Breast cancer diagnosis | Participants were in early-stage treatment | Psychosocial Intervention (PI)  ***Intervention Characteristics:*** The format of PI consisted of group intervention for women with breast cancer. There were eight weekly two-hour sessions, and each group was composed of eight to twelve participants led by two therapists. PI was delivered in-person. The content PI focused on teaching patients to routinely use thought records, practice problem-solving and cognitive restructuring, communicate better with caregivers and health professionals through role-play, and practice relaxation.  ***Provider:*** PI was delivered by psychologists or psychiatrists, trained in group therapy and BCT. | Anxiety;  Mood state; | STAI  POMS |
| Edelman et al., 1999 | T = 43  C = 49 | Age = 50.0  %Female = 100%  %White = NR | Waitlist or attention control | Breast cancer diagnosis | Participants were in on-going active treatment | Cognitive Behavior Therapy (CBT)  ***Intervention Characteristics:*** The format of CBT consisted of individual-based therapy without other detailed information. CBT was delivered primarily in-person. The content of CBT here focused on teaching participants basic cognitive skills, including how to identify and challenge maladaptive thoughts and beliefs. Behavioral techniques included relaxation tapes and effective communication.  ***Provider:*** CBT was provided by two therapists with training and experience in facilitating group therapy with cancer patients. | Anxiety;  Depression;  Anger;  Vigour;  Fatigue;  Confusion;  Mood Disturbance;  Self-Esteem. | PMS  TMD  CSI-AD |
| Edelman et al., 1999 | T = 23  C = 24 | Age = 48.0 (median)  %Female = 100%  %White = NR | Active comparator | Primary breast cancer diagnosis | Participants were in on-going active treatment | Cognitive Behavior Therapy Intervention (CBT Intervention)  ***Intervention Characteristics:*** The format of CBT Intervention consisted of group sessions that were conducted in accordance with a prepared therapists’ manual. CBT Intervention delivered primarily in-person. The content of the CBT Intervention aimed to make participants aware of the thoughts and underlying beliefs which contribute to distress and negative responses, and to restructure these into more adaptive ways of thinking. The second half of each session comprised training in behavioral strategies such as deep relaxation, problem solving, and goal setting  ***Provider:*** The CBT Intervention was provided by two female therapists, of which at least one was a registered psychologist. | Anxiety  Depression  Anger  Vigor  Self-esteem  QoL | POMS  FLI |
| Edmonds et al., 1999 | T = 30  C = 36 | Age = 50.68  %Female = 100%  %White = NR | Treatment as usual or standard care | Metastatic breast cancer diagnosis | Participants were receiving active and on-going treatment | Long Term Group Therapy (LTGT)  ***Intervention Characteristics:*** The format LTGT consisted of group therapy with three components: (1) participants completed thirty-five weekly sessions of group meetings which lasted two hours, with approximately eight members per group; (2) then participants completed a twenty-week course of standard cognitive behavioral assignments; and, finally, (3) intervention subjects were asked to take an intensive weekend coping skills training course during the first 2-4 months. The content of LGTG was based on the work of Yalom and included mutual support, problem solving, facing the likelihood of one’s own health and death of other members, and providing encouragement to ventilate and integrate emotional reactions to the disease. Intervention homework included monitoring thoughts and behaviors, introducing substitute thoughts and behaviors; exploring resistance to change, and with insights gained to develop a new, more balanced lifestyle. The intervention was delivered in-person.  ***Provider:*** LGTG was provided by therapists experienced in the techniques used in previous studies. The principal therapist was a registered clinical psychologist with over 20 years’ experience. | Mood;  QoL;  Mental adjustment;  Emotional wellness. | POMS  FLIC  MAC  MCSD |
| Ferguson et al., 2016 | T = 27  C = 20 | Age = 54.6  %Female = 100%  %White = 100% | Active comparator | Breast cancer diagnosis | Participants were in post-treatment survivorship | Memory and Attention Adaptation Training (MAAT)  ***Intervention Characteristics:*** MAAT consisted of eight weekly visits of thirty to forty-five minutes each. The intervention was delivered in-person. The content of MAAT focused on four components: (1) education concerning chemotherapy related cognitive dysfunction; (2) self-awareness training to identify “at-risk” situations in which cognitive failures may occur; (3) stress management and self-regulation (relaxation, cognitive restructuring, activity pacing, and sleep quality improvement); and (4) cognitive compensatory strategies  ***Provider:*** MAAT was provided by the study investigator, who was Phd.-level psychologist. | Perceived cognitive impairment; | FACT-Cog  MIA-Anxiety  DASS |
| Garland et al., 2014 | T = 47  C = 64 | Age = 58.89  %Female = 72%  %White = 90% | Active comparator | Multiple cancer diagnoses | Participants were in post-treatment survivorship | Cognitive Behavioral Therapy Insomnia (CBT-I)  ***Intervention Characteristics:*** The format of CBT-I consisted of groups of six to ten individuals meeting over the course of eight, weekly, ninety-minute sessions, for a total of twelve total contact hours. CBT-I was delivered in-person. The content of CBT-I followed previously published CBT-I trials in patients with cancer. CBT-I contains the following four individually validated strategies: stimulus control, sleep restriction, cognitive therapy, and relaxation training. Combined, this intervention targets and reduces sleep-related physiologic and cognitive arousal to re-establish restorative sleep function.  ***Provider:*** CBT-I was provided by a doctoral-level student in clinical psychology, with training in CBT-I and supervised by a clinical psychologist. | Insomnia;  General stress;  Mood state;  Dysfunctional beliefs and attitude | ISI  PSQI  C-SOSI  POMS-SF  DBAS-16 |
| Germino et al., 2012 | T = 167  C = 146 | Age = 44.0  %Female = 100%  %White = 63% | Waitlist or attention control | Breast cancer diagnosis | Participants were in post-treatment survivorship | Younger Breast Cancer Survivor Uncertainty Management Intervention (YS-UMI)  ***Intervention Characteristics:*** The format of YS-UMI was a scripted, professionally acted and produced CD that provided specific cognitive and behavioral strategies to control uncertainty and to promote self-efficacy for dealing with thoughts of recurrence, to improve disclosure of cancer-related concerns to others and to make positive life changes. Materials were supplemented by four 20-minute weekly training calls conducted by trained nurse interventionists using a scripted protocol and planned appointment times. YS-UMI was delivered in-person and via technology platforms. The content of YS-UMI centered on cognitive restructuring and problem solving.  ***Provider:*** YS-UMI was provided by a nurse interventionist. | Negative Affect | PNAS |
| Gielissen et al., 2006 | T = 50  C = 48 | Age = 44.94  %Female = 49%  %White = NR | Waitlist or attention control | Multiple cancer diagnoses | Participants were all are disease-free cancer survivors | Cognitive Behavior Therapy (CBT)  ***Intervention Characteristics:*** The format of CBT consisted of eight weekly two-hour group sessions. CBT was delivered in-person, with at home practice for participants available. The content of CBT here focused on intensive training in mindfulness and treatment directed by the original MBCT manual. Focus areas included: psychoeducation, mindfulness practices, and cognitive-based discussions about depression and anxiety related to cancer.  ***Provider:*** CBT was facilitated by Elizabeth Foley, a senior clinician in mindfulness practice and CBT. | Fatigue;  Functional health;  Psychological distress. | CIS-fatigue  SIP-8  SCL-90 |
| Goode et al., 2011 | T_1_ = 70  T_2_ = 70  C = 68 | Age = 66.66  %Female = 0%  %White = 75% | Waitlist or attention control | Prostate cancer diagnosis | Participants were active patients and in post-treatment survivorship | Behavioral Therapy (BT)  ***Intervention Characteristics:*** The format of BT consisted of four visits approximately two weeks apart. BT was delivered in-person. The content of BT focused on psycho-education, behavioral activation, and problem-solving.  ***Provider:*** BT was provided by physician investigators or nurse practitioners. | Functional health;  Quality of life;  Symptom severity. | EPIC  IIQ  SF-36  AUA |
| Gregoire et al., 2017 | T_1_ = 68  T_2_ = 21  T_3_ = 10  C = 24 | Age = 53.81  %Female = 100%  %White = NR | Waitlist or attention control | Breast cancer diagnosis | Participants were receiving on-going active treatment | Cognitive Behavioural Therapy (CBT)  ***Intervention Characteristics:*** The format of CBT consisted of six weekly ninety-minute sessions in groups of three to eight participants. CBT was delivered in-person. The content of CBT focused on a manual utilized in other published studies, explicitly listing relaxation.  ***Provider:*** CBT-trained psychologists. | Depression;  Anxiety;  Fatigue;  Insomnia. | HADS  EORTC QLQ C 30 |
| Gudenkauf et al., 2015 | T = 55  C_1_ = 70  C_2_ = 58 | Age = 52.82  %Female = 100%  %White = NR | Active comparator  Waitlist or attention control | Breast cancer diagnosis | Participants had recently completed surgery | Cognitive Behavioral Training (CBT)  ***Intervention Characteristics:*** The format of CBT consisted of a five-week group intervention protocol. CBT was delivered in person. The content of CBT was modeled after Antoni’s CBSM programme. Taught adaptive coping skills to manage daily stressors and encouraged utilization of social resources, with a special emphasis on issues related to cancer and treatment. Thought monitoring, cognitive restructuring, coping skills training, and skill for social support utilization, anger management to promote conflict resolution and assertiveness training.  ***Provider:*** No information on the provider(s) of CBT was available. | Depression;  Distress;  Emotional wellbeing. | ABS  IES-R  FACT |
| Hummel et al., 2017 | T = 84  C = 85 | Age = 51.10  %Female = 100%  %White = NR | Waitlist or attention control | Breast cancer diagnosis | Participants were in post-treatment survivorship | Internet-based Cognitive Behavioral Therapy (iCBT)  ***Intervention Characteristics:*** The format of iCBT consisted of approximately twenty weekly sessions that had to be completed within a maximum period of twenty-four weeks. iCBT was delivered via the internet. The content of iCBT centered around ten modules, with a focus on cognitive restructuring, behavioral activation, and problem solving.  ***Provider:*** iCBT was provided by a personal psychologist or sexologist. | Sexual function;  Psychological distress. | FSFI  SAQ2  FSDS-R  FACT  HADS |
| Irwin et al., 2017 | T = 45  C = 45 | Age = 59.80  %Female = 100%  %White = 85% | Active comparator | Breast cancer diagnosis | Participants were in post-treatment survivorship | Mindfulness-Based Cognitive Therapy (MBCT)  ***Intervention Characteristics:*** The format of MBCT consisted of groups of seven to ten participants in weekly one-hundred-twenty-minute sessions. MBCT followed a content format informed by previously published trials, and contained five validated components: cognitive therapy, stimulus control, sleep restriction, sleep hygiene, and relaxation.  ***Provider:*** MBCT was provided by therapists who were experienced in CBT-I. | Sleep quality;  Fatigue;  Depression. | PSQI  AISI  MDFSI  ESS  IDSC |
| Johannsen et al., 2016 | T = 67  C = 62 | Age = 56.75  %Female = 100%  %White = NR | Waitlist or attention control | Breast cancer diagnosis | Participants were in post-treatment survivorship | Mindfulness-Based Cognitive Therapy (MBCT)  ***Intervention Characteristics:*** The format of MBCT consisted of group sessions that occurred weekly over the course of eight weeks. MBCT was delivered in-person. The content of MBCT adhered to a manual with specific modifications to study population. Mindfulness exercises and psychoeducation, cognitive exercises for pain catastrophizing, and homework mindfulness exercises were included. Also included were cognitive therapy exercises, with particular attention to cancer-specific rumination and physical problems.  ***Provider:*** MBCT was provided by a trained mindfulness instructor. | Pain;  QoL;  Psychological distress. | SF-MPQ-2  PPI  WHO-5  HADS |
| Johannsen et al., 2018 | T = 67  C = 62 | Age = 56.75  %Female = 100%  %White = NR | Waitlist or attention control | Breast cancer  diagnosis | Participants were in post-treatment survivorship | Mindfulness-Based Cognitive Therapy (MBCT)  ***Intervention Characteristics:*** The format of MBCT consisted of an eight-week protocol adhering to a program outlined in the original manual (see full text for specific citation). MBCT was provided in-person. Based on the manual mentioned above, MBCT content included mindfulness exercises and psychoeducation, cognitive exercises for pain catastrophizing, and homework mindfulness exercises. Also included cognitive therapy exercises, with particular attention to cancer-specific rumination and physical problems.  ***Provider:*** MBCT was provided by an experienced mindfulness instructor with training from Oxford University, receiving supervision from the center for mindfulness research and practice. | Pain;  Self-compassion;  Pain catastrophizing. | NRS  SCS-SF  PCS |
| Johansson et al., 2008 | T_1_ = 134  T_2_ = 117  T_3_ = 104  C = 126 | Age = 63.88  %Female = 58%  %White = NR | Treatment as usual or standard care | Multiple cancer diagnoses at multiple stages | N/A | Individual Support (IS)  ***Intervention Characteristics:*** The format of IS consisted of a frequency and intensity that varied based on the specific group. There was a median of three sessions of psychologist contact. IS was delivered in-person. The content of IS utilized techniques derived from cognitive behaviour therapy, including relaxation techniques, identification and challenging of negative automatic thoughts and activity scheduling and daily planning.  ***Provider:*** IS was provided by a project psychologist. | QoL;  Functional health;  Fatigue;  Pain;  Insomnia. | EORTC QLQ-C30 |
| Kangas et al., 2013 | T = 21  C = 14 | Age = 54.80  %Female = 20%  %White = NR | Active comparator | Multiple cancer diagnoses | Participants were receiving on-going treatment | Multi-modal Cognitive Behavioral Therapy Program (CBT)  ***Intervention Characteristics:*** The format of CBT consisted of an individually administered program comprised six consecutive weekly ninety-minute sessions with structured homework activities and a seventh booster session conducted four weeks after session six. CBT was delivered in-person. The content of the CBT program was adapted from early CBT interventions for trauma and comorbid depression and included behavioral activation tailored to coping with HNC treatment and recovery. The program focused on the following areas: (1) psychoeducation pertaining to HNC and illness-related stress; (2) breathing and relaxation training; (3) imaginal exposure to distressing cancer-related memories; (4) graded in vivo exposure; (5) cognitive restructuring; and (6) Behavioral activity scheduling.  ***Provider:*** CBT was provided by four masters-level clinical psychologists. | Posttraumatic stress;  Depression;  Anxiety;  Posttraumatic cognition;  Functional health. | PCL-S  BDI-II  PTCI  FACT |
| Kazak et al., et al., 2004 | T = 25  C = 30 | Age = 14.64  %Female = 52%  %White = NR | Waitlist or attention control | Multiple cancer diagnoses | Participants were in post-treatment survivorship | Surviving Cancer Competently Intervention Program (SCCIP)  ***Intervention Characteristics:*** The format of SCCIP consisted of a four-session, one-day manualized intervention to reduce PTSS in adolescent survivors of childhood cancer and their families, utilizing a family group treatment model. SCCIP was delivered in-person. The focus of content in SCCIP was broken up by session: (1-2) used cognitive-behavioral principles to reduce distress and were conducted with survivors, mothers, fathers, and siblings in separate groups; and (3-4) utilized family therapy approaches with the same aim, now with all members together.  ***Provider:*** SCCIP was provided by psychologists, psychology postdoctoral fellows, psychology graduate students and interns, nurses, and social workers who received intensive training. | Pain;  Sleep;  Fatigue;  Depression;  Cancer distress. | NRS-11  MOS-9  PROMIS  PHQ-8  PCS  CTDX |
| Lau et al., 2020 | T = 81  C = 76 | Age = 60.04  %Female = 60%  %White = 0% | Active comparator | Lung cancer diagnosis | Participants were receiving on-going active treatment | Cognitive Behavioral Therapy (CBT)  ***Intervention Characteristics:*** The format of CBT consisted of a closed-group format with eight to twelve participants per group and consisted of eight weekly three-hour sessions facilitated by two to three trained facilitators. CBT was delivered in-person. The content of CBT focused on teaching techniques of relaxation (e.g., breathing, muscle relaxation), identifying dysfunctional coping patterns, thoughts and values, the use of a mood diary, reappraisal, cognitive continuum, cost-benefit analysis, and positive self-statements for adjusting dysfunctional emotional responses and thoughts. Also constructed an implemented plan of pleasurable activities.  ***Provider:*** CBT was provided by a trained facilitator. | Functional health;  QoL;  Holistic wellbeing;  Depression;  Anxiety;  Dysfunctional attitude. | FACT-G  EORTC QLQ-30  HWS  HADS  DFAS |
| Lee et al., 2011 | T = 35  C = 36 | Age = NR  %Female = 100%  %White = NR | Waitlist or attention control | Breast cancer diagnosis | Participants were undergoing radiotherapy | Cognitive-Behavior Therapy (CBT)  ***Intervention Characteristics:*** The format of CBT consisted of a group session offered once a week for six weeks, with each session lasting between fifty and one-hundred-twenty minutes, with five to eight participants per group. CBT was delivered in-person. The content of CBT centered on session-based focus areas, which occurred in the following sequence:  (1) psychoeducation about CBT; (2) introducing the cognitive process (including cognitive restructuring); (3-5) cognitive distortion and cognitive restructuring; and (6) behavioral activation and problem solving.  ***Provider:*** CBT was provided by nurses. | Fatigue;  QoL. | RPFS  QoL Scale |
| Locke et al., 2008 | T = 12  C = 7 | Age = 50.15  %Female = 38%  %White = NR | Treatment as usual or standard care | Primary brain tumor diagnosis | Participants were receiving on-going active treatment | Cognitive Rehabilitation and Problem-Solving (CRPS)  ***Intervention Characteristics:*** The format of CRPS consisted of the completion of six fifty-minute sessions in an approximately two-week period for the cognitive rehabilitation portion and a problem-solving portion that also involved six fifty-minute sessions over a two-week period featuring specific goals. CRPS was delivered in-person. The content of CRPS centered around the following focus areas: (1) use of a calendar to compensate for cognitive symptoms; (2) problem-solving therapy modeled after the techniques described by Nezu and colleagues. Intervention teaches the patients and caregiver a model of stress and a specific positive problem-solving technique for its management.  ***Provider:*** CRPS was provided by neuropsychologist and master’s level behavioral therapist. | QoL;  Function; | FACT  MPAI-4  LASA  POMS |
| Mann et al., 2012 | T = 47  C = 49 | Age = 53.61  %Female = 100%  %White = 85% | Treatment as usual or standard care | Breast cancer diagnosis | Participants were in post-treatment survivorship | Group Cognitive Behavioural Therapy (CBT)  ***Intervention Characteristics:*** The format of group CBT consisted of ninety-minute sessions every week for six weeks. CBT delivery was self-paced, aside from the first training session, which was supervised. The content of CBT followed a treatment manual, which was produced in advance of the study. Content was session-focused, based on the following focus areas each session: (1) introduce the cognitive behavioural model; (2) focus on the role of stress in potentiating HFNS and CBT strategies for the reduction of stress and anxiety; (3) focus on cognitive and behavoural reactions to hot flushes; (4) focus on understanding night sweats and improving sleep habits and the application of behavioural strategies to reduce wakefulness after night sweats; (5) focus on the cognitive component of sleep problems; and (6) review throughout the session.  ***Provider:*** CBT was provided by a clinical psychologist who was trained to deliver the sessions with the help of an assistant. | Hot flushes and night sweats;  Depressed mood;  Insomnia;  Distress;  Anxiety;  QoL | HFNS  WHQ  SF-36 |
| Matthews et al., 2014 | T = 30  C = 26 | Age = 52.48  %Female = 100%  %White = NR | Waitlist or attention control | Breast cancer diagnosis at  stages I, II, and III | Participants were in post-treatment survivorship | Cognitive Behavioral Therapy for Insomnia (CBTI).  ***Intervention Characteristics:*** The format of CBTI consisted of individual weekly sessions. CBTI was delivered in person for sessions one through three, while sessions four and five were conducted by phone. The content of CBTI was structured with week-to-week content, including psychoeducation, cognitive restructuring, and behavioral activation.  ***Provider:*** CBTI was provided by nurses with CBTI training. | Insomnia severity;  QoL;  Physical function;  Attention function;  Fatigue;  Depression;  Anxiety;  Sleep attitude;  Sleep knowledge. | ISI  EORTC QLQ-C30  AFI  PFS  HADS  DBAS-16  PKT |
| May et al., 2009 | T = 76  C = 71 | Age = 48.8  %Female = 83%  %White = NR | Treatment as usual or standard care | Multiple cancer diagnoses | Participants were in post-treatment survivorship | Cognitive Behavioral Therapy (CBT) details described elsewhere see original article  ***Intervention Characteristics:*** The format of CBT consisted of groups of eight to twelve cancer survivors meeting once a week, for two hours each session. CBT was delivered in-person. The content of CBT was based on principles of self-management, i.e., goal selection, information collection, information processing and evaluation, decision making, action, and self-reaction. CBT was also based on a cognitive behavioral problem-solving protocol. Key session components included cognitive restructuring, behavioral activation, and problem solving.  ***Provider:*** CBT was provided by a psychologist and a social worker. | QoL;  Physical activity; | EORTC QLQ-C30  PASE |
| Mendoza et al., 2017 | T = 25  C = 30 | Age = 60.95  %Female = 89%  %White = NR | Treatment as usual or standard care | Multiple cancer diagnoses | Participants were in either active treatment or post-treatment survivorship | Valencia Model of Waking Hypnosis and Cognitive Behavioral Therapy (VMWH-CBT)  ***Intervention Characteristics:*** The format of VMWH-CBT consisted of four sessions of treatment that combined training in self-hypnosis with CBT. Each session lasted about an hour. VMWH-CBT was delivered in-person.  The content of VMWH-CBT centered around teaching participants to identify and restructure any unhelpful thoughts regarding their symptoms using CBT methods. Psychoeducation about pain, fatigue, and sleep problems and learned behavioral strategies to cope with them during the study to facilitate the maintenance of treatment gains.  ***Provider:*** VMWH-CBT was provided by the study clinician, who was not described further. | Pain;  Sleep problem;  Fatigue;  Depression;  Cancer distress. | NRS-11  MOS-9  PHQ-8  PROMIS  PCS  CTDX |
| Mishel et al., 2002 | T = 80  C = 80  [one arm dropped] | Age = 64.0  %Female = 0%  %White = 56.5% | Waitlist or attention control | Localized prostate carcinoma diagnosis | Participants were receiving on-going treatment | Uncertainty Management Intervention (UMI)  ***Intervention Characteristics:*** The format of UMI consisted of a weekly telephone call for eight consecutive weeks with a nurse. UMI was delivered by telephone. The content of UMI focused primarily on strategies of uncertainty management skills, including cognitive reframing (cognitive restructuring) and problem solving.  ***Provider:*** UMI was provided by a nurse who was trained in the intervention. | Cognitive coping strategy;  Symptom severity. | CSQ  SDS |
| Moon et al., 2020 | T = 11  C = 11 | Age = 63.0  %Female = 50%  %White = 0% | Active comparator | Multiple cancer diagnoses | Participants were receiving on-going treatment | Cognitive Behavioral Therapy Insomnia (CBT-I)  ***Intervention Characteristics:*** The format of CBT-I consisted of sessions once per week for four weeks. CBT-I was delivered in-person. The content of CBT-I centered on sleep hygiene, relaxation, sleep restrictions, stimulus control, and cognitive therapy (including cognitive restructuring).  ***Provider:*** CBT-I was provided by a therapist who had over five years of clinical experience. | Insomnia;  Sleep quality;  Anxiety;  Fatigue;  QoL. | ISI  PSQI  ESS  SAS  BFI  EQ-5D |
| Mosher et al., 2016 | T = 51  C = 55 | Age = 62.69  %Female = 53%  %White = 91% | ACT | Lung cancer diagnosis | Participants were receiving on-going treatment | Telephone Symptom Management (TSM)  ***Intervention Characteristics:*** The format of TSM consisted of four individualized sessions delivered via telephone. The content of CSM focused on session-specific material. During the four sessions, the patient and caregiver received instruction in symptom management strategies, including relaxation exercise, problem solving, cognitive restructuring, emotion-focused/self-soothing approaches, communication skills, pleasant activity scheduling, and activity pacing.  ***Provider:*** TSM was provided by a social worker. | Depression  Pain  Fatigue  Breathlessness  Self-efficacy  Social constraints | PHQ-8  GAD-7  BPI-SF  FSI  SES |
| Nezu et al., 2003 | T_1_ = 45  T_2_ = 43  C = 44 | Age = 47.24  %Female = 67%  %White = 77% | Waitlist or attention control | Multiple cancer diagnoses | Participants were receiving on-going active treatment | iCanADAPT Early (iCBT program)  ***Intervention Characteristics:*** The format of the iCBT program consisted of individual sessions, delivered on a weekly basis, one-and-one-half hours per session and for ten sessions. The iCBT program was delivered in-person. The content of iCBT program focused on empirically validated problem-solving training manuals originally developed for major depressive disorder and revised specifically for cancer population. Rational, positive, and constructive set of cognitive appraisal to problems in living and to problem solving as a means of coping.  ***Provider:*** The iCBT program was provided by fifteen advanced psychology graduate students and three social workers. All had master’s degrees and two years’ experience providing psychotherapy to medical patients. | QoL  Depression  Mood  Distress  Rehabilitation | QL Index  HRSD  POMS  BSI  CARES |
| Nissen et al., 2020 | T = 104  C = 46 | Age = 55.45  %Female = 91%  %White = NR | Waitlist or attention control | Breast and prostate cancer diagnosis | Participants were in post-treatment survivorship | Internet-delivered Mindfulness-Based Cognitive Therapy (iCMBT)  ***Intervention Characteristics:*** The format of iCMBT consisted of eight modularized sessions. iCBMT was delivered over the internet but included the supervision from a therapist. The content of the eight modules (each assigned to weekly sessions) included written material, audio exercises, writing tasks, cancer-specific patient examples, and video with patients and experts. Participants also completed a weekly training diary and mailed it to their therapist who gave written, asynchronous feedback on a prearranged day of the week.  ***Provider:*** iCMBT was provided by eight master’s level psychology students trained in MBCT, and one experienced psychologist trained in BMCT provided therapist feedback. | Anxiety  Depression  Stress  QoL  Insomnia | STAI  BDI-II  PSS  WHO-5  ISI |
| Norouzi et al., 2017 | T = 12  C = 12 | Age = 38.80  %Female = 100%  %White = NR | Waitlist or attention control | Breast cancer diagnosis | Participants were receiving on-going treatment | Mindfulness-Based Cognitive Therapy (MBCT)  ***Intervention Characteristics:*** The format of MBCT consisted of eight sessions, which were delivered in-person. The content of MBCT focused on psychoeducation, automatic guidance system; facing obstacles; mindfulness meditation; behavioral activation; cognitive restructuring; and sleep hygiene and other problem-solving related issues.  ***Provider:*** MBCT was provided by individuals with a master’s degree in clinical psychology and PhD degree in health psychology. | Posttraumatic growth;  Self-management;  Functional disability. | PTGI  PAM |
| Park et al., 2020 | T = 38  C = 36 | Age = 53.7  %Female = 100%  %White = 0% | Waitlist or attention control | Breast cancer diagnosis | Participants were receiving on-going active treatment | Mindfulness-Based Cognitive Therapy (MBCT)  ***Intervention Characteristics:*** The format of MBCT consisted of an eight-week program, with two hours each week dedicated to an in-person group session, with groups consisting of four to nine participants. The content of MBCT centered on formal meditational exercises, psychoeducation based on cognitive therapy, and discussion and interaction among the participants to facilitate their learning. Homework was assigned to the participants at every session, which was supposed to take twenty to forty-five minutes each day.  ***Provider:*** MBCT was provided by clinical psychologists, psychiatrists, and nurses who had five to seven years of mindfulness experience and had undergone MBCT training provided by the Oxford Mindfulness Center. | Distress;  Depression;  Anxiety;  Fear of cancer recurrence;  Fatigue;  Functional health. | HADS  FCRI  BFI  FACIT-FS  FACT |
| Penedo et al., 2020 | T = 95  C = 97 | Age = 68.84  %Female = 0%  %White = 58.9% | Active comparator | Advanced prostate cancer | N/A | Cognitive Based Stress Management (CBSM)  ***Intervention Characteristics:*** The format of CBSM consisted of weekly group sessions, which each lasted approximately ninety minutes. CBSM was delivered in-person. The content of CBSM integrated cognitive-behavioral stress- and self-management skills (e.g., cognitive restructuring, with relaxation skills training to improve quality of life and reduce symptoms). CBSM included behavioral activation, such as muscle relaxation, deep breathing, imagery, and meditation. CBSM also included homework assignments.  ***Provider:*** CBSM was provided by master’s- or doctoral-level therapists who completed an in-person facilitator training. | Functional health;  Depression;  Fatigue;  Pain;  Anxiety;  Distress | FACT  EPIC  PROMIS-depression  FSI  MPQ  MAX-PC  IES-R  MOCS  PSS  ABS  SIP |
| Qiu et al., 2013 | T = 31  C = 31 | Age = 50.63  %Female = 100%  %White = NR | Waitlist or attention control | Breast cancer diagnosis | Participants were receiving no post-treatment survivorship | Group Cognitive Behavioral Therapy (GCBT)  ***Intervention Characteristics:*** The format of GCBT consisted of a closed, treatment protocol-guided group intervention, met in-person weekly over the course of ten two-hour sessions. One month following the end of the intervention, one booster session was provided. The content of GCBT followed a protocol based on Beck’s principle of CBT, including: (1) cognitive restructuring; (2) behavioral activation; and (3) interpersonal communications.  ***Provider:*** GCBT was provided by one therapist who is a psychiatrist. | Depression;  Anxiety;  Self-Esteem;  Functional Health. | HADS  SAS  SES*  FACT-B |
| Ren et al., 2019 | T = 98  C_1_ = 98  C_2_ = 196 | Age = 47.06  %Female = 100%  %White = 0% | Active comparator  Treatment as usual or standard care | Breast cancer diagnosis | Participants were in post-treatment survivorship | Cognitive Behavioral Therapy (CBT)  ***Intervention Characteristics:*** The format of CBT consisted of nine sessions based on the manual codified by Aaron T. Beck and took twelve weeks to complete. Sessions one through five were delivered once a week, while sessions six through nine were delivered every 2 weeks. All sessions were delivered in-person. The content of CBT focused on combined behavioral, cognitive, and educational strategies. Participants were taught how to identify and restructure any unhelpful thoughts. Participants were also taught behavioral strategies and some psychological techniques to cope with psychological distress.  ***Provider:*** CBT was provided by two trained therapists. | Functional health. | FACT-B |
| Ritterband et al., 2012 | T = 14  C = 14 | Age = 56.70  %Female = 86%  %White = 93% | Waitlist or attention control | Multiple cancer diagnoses | Participants were in post-treatment survivorship | Internet Intervention for Insomnia (III)  ***Intervention Characteristics:*** The format of III consisted of six interactive cores with each core lasting between forty-five to sixty minutes in duration. III was an internet-delivered program, which focused on psychoeducation, behavioral activation, and problem solving.  ***Provider:*** There was no provider involved, as III was an online program. | Sleep problem;  Fatigue;  Depression;  Anxiety;  QoL. | MFSI-SF  HADS  SF-12 |
| Rogers et al., 2017 | T = 110  C = 112 | Age = 54.4  %Female = 100%  %White = 98% | Treatment as usual or standard care | Breast cancer diagnosis | Participants were in post-treatment survivorship | Better Exercise Adherence after Treatment for Cancer (BEAT Cancer)  ***Intervention Characteristics:*** The format of BEAT Cancer consisted of twelve supervised exercise sessions with a trained exercise specialist. During the second six weeks of the intervention, participants attended face-to-face update counseling sessions every two weeks. The first nine weeks of the intervention included six group discussions. BEAT Cancer was delivered in-person. BEAT Cancer’s content focused on self-efficacy, exercise barriers, behavioral capability, goal setting with self-monitoring, behavioral modification strategies, time management, stress management, safety, cognitive reframing, relapse prevention, and role models.  ***Provider:*** BEAT Cancer was provided by an exercise specialist. | Fatigue;  Depression;  Anxiety. | FSI  HADS |
| Sandgren et al., 2000 | T = 17  C = 10 | Age = 51.23  %Female = 100%  %White = 98% | Waitlist or attention control | Breast cancer diagnosis | Participants were receiving on-going treatment | Telephone-Delivered Cognitive Therapy (TDCT)  ***Intervention Characteristics:*** The format of TDCT consisted of up to ten telephone calls to the participant’s home. Phone calls were administered once a week for four weeks and then every other week for six more sessions. Each phone session lasted up to thirty minutes, averaging twenty to twenty-five minutes. The content of TDCT focused on providing support, teaching coping skills, managing anxiety and stress, and helping to solve patient-generated problems (e.g., interpersonal problems, problems returning to work). Therapists were trained to use cognitive restructuring, a technique that involves identifying erroneous beliefs or catastrophic thinking and implementing plausible alternative thoughts. Therapists also encouraged emotional expression, provided nonspecific support, and used problem-solving and relaxation techniques.  ***Provider:*** TDCT was provided by female clinical psychology master’s candidates. | Mood States including:   1. Anger 2. Depression 3. Fatigue 4. Vigor 5. Anxiety 6. Confusion | POMS |
| Savard et al., 2005 | T = 27  C = 30 | Age = 54.05  %Female = 100%  %White = NR | Waitlist or attention control | Breast cancer diagnosis | Participants were in post-treatment survivorship | Cognitive Behavioral Therapy for Insomnia (CBT-I)  ***Intervention Characteristics:*** The format of CBT-I consisted of eight weekly sessions, which were approximately ninety minutes in duration, and offered in-person to groups consisting of four to six patients. The content of CBT-I was centered around a protocol developed from procedures by Morin and was slightly adapted for a cancer-diagnosed population. The multimodal approach combined behavioral (i.e., stimulus control therapy, sleep restriction), cognitive (i.e., cognitive restructuring), and education (i.e., sleep hygiene, fatigue, and stress management) strategies that were described in a treatment manual given to all participants.  ***Provider:*** CBT-I was provided by a master-level psychologist with experience in the administration of this treatment protocol. | Insomnia;  Depression;  Anxiety;  Fatigue;  QoL | ISI  HADS  MFI  QLQ-C33 |
| Savard et al., 2006 | T = 21  C = 16 | Age = 51.6  %Female = 100%  %White = 100% | Waitlist or attention control | Breast cancer diagnosis | Participants were individuals with metastatic breast cancer receiving on-going treatment | Cognitive Therapy (CT)  ***Intervention Characteristics:*** The format of CT consisted of weekly individual sessions lasting sixty to ninety minutes occurring over the course of eight weeks. CT was delivered in-person. The content of CT focused on strategies that were elaborated on by Beck and colleagues. Psychoeducation of cognitive theory of emotion, behavioral activation, cognitive restructuring, and problem-solving.  ***Provider:*** CT was provided by two licensed psychologists with experience in cognitive therapy. | Depression;  Anxiety;  Fatigue;  Insomnia;  QoL. | BDI  HADS-D  HADS-A  MFI  ISI  QLQ-C33-global |
| Stefanopoulou et al., 2015 | T = 33  C = 35 | Age = 68.87  %Female = 0%  %White = 74% | Treatment as usual or standard care | Prostate cancer diagnosis | Participants were in post-treatment survivorship | Self-Help Cognitive Behavioral Therapy (CBT)  ***Intervention Characteristics:*** The format of CBT consisted of a four-week self-help intervention utilizing a booklet. The content of CBT focused on psychoeducation of CBT, symptoms; cognitive restructuring; behavioral activation; and problem-solving.  ***Provider:*** There was no provider involved, as this was a CBT self-help program. | Symptom frequency;  Depression;  Anxiety;  QoL. | HFNS frequency  HADS depression  HADS anxiety  EORTC QOL |
| Strong et al., 2008 | T = 101  C = 90 | Age = 56.6  %Female = 71%  %White = NR | Treatment as usual or standard care | Multiple cancer diagnoses | Participants were receiving active on-going treatment | SMaRT oncology 1, Depression Care for People with Cancer (DCPC)  ***Intervention Characteristics:*** DCPC consisted of ten one-to-one sessions over three months, preferably in person at the cancer center, but occasionally by telephone or at participants’ homes. The content of DCPC focused on education about depression and its treatment (including antidepressant medication); problem-solving treatment to teach the patients coping strategies designed to overcome feelings of helplessness; and communication about management of major depressive disorder.  ***Provider:*** DCPC was provided with the participants’ oncologist and primary care doctor. | Depression  Anxiety  Pain | SCL  EORTC QLQ C30 |
| Syrjala et al., 2018 | T = 115  C_1_ = 114  C_2_ = 115 | Age = 52.0  %Female = 44%  %White = 93% | Active comparator  Waitlist or attention control | Multiple cancer diagnoses | Participants were in post-treatment survivorship | Problem-Solving Therapy (PST)  ***Intervention Characteristics:*** The format of PST consisted of four to eight sessions of individual problem-solving therapy, with sessions occurring approximately two weeks apart. PST was delivered over the phone. The content of PST was delivered based on session. The first session explained PST, collected an initial problem list, and applied the PST process to one problem. Subsequent sessions lasted thirty minutes and applied PST to one problem per session.  ***Provider:*** PST was provided by Ph.D.-level psychologists. | Cancer distress;  Depression;  Fatigue;  Physical function. | CTDX  SCL-90-R depression  FSI  SF-36 |
| van de Wal et al., 2017 | T = 45  C = 43 | Age = 58.83  %Female = 53%  %White = NR | Treatment as usual or standard care | Breast, prostate, and colorectal cancer diagnoses | Participants were in post-treatment survivorship | Blended Cognitive Behavior Therapy (bCBT)  ***Intervention Characteristics:*** The format of bCBT consisted of five individual one-hour sessions, accompanied by three fifteen-minute e-consultations. bCBT was delivered through a combination of in-person and virtual methods. The content of bCBT centered around techniques including psychoeducation, cognitive restructuring, and behavioral modification.  ***Provider:*** bCBT was provided by psychologists with more than five years of experience. | Fear of cancer; reoccurrence;  Psychological distress;  Body vigilance;  Fatigue;  QoL. | CWS  FCRI  HADS  DT  BVS  CIS-8R  EORTC QOQ-C30  SWLS  LOT |
| van den Berg et al., 2015 | T = 70  C = 80 | Age = 50.77  %Female = 100%  %White = NR | Treatment as usual or standard care | Breast cancer diagnosis | Participants were in post-treatment survivorship | Breast Cancer E-Health (BREATH)  ***Intervention Characteristics:*** The format of BREATH consisted of a fixed sixteen-week modular self-help online program, with four phases of adjustment to breast cancer. The content in BREATH’s components are based on cognitive behavioral therapy and include information, assignment, assessment, and video.  ***Provider:*** BREATH was a self-help program, with no therapist contact. | Distress;  Depression;  Anxiety;  Fear of cancer recurrence;  Self-efficacy. | SCL-90  HADS  CIS  IES  EORTC QLQ |
| van der Lee et al., 2012 | T = 59  C = 24 | Age = 52.03  %Female = 84%  %White = 100% | Waitlist or attention control | Multiple cancer diagnoses | Participants were receiving on-going treatment | Mindfulness-Based Cognitive Therapy (MBCT)  ***Intervention Characteristics:*** The format of MBCT consisted of eight two-and-one-half-hour sessions, followed by one long six-hour session, and concluded with one two-and-one-half-hour follow-up session (provided two months after the ninth session). MBCT was provided in-person. The content of MBCT followed a protocol, based on the published protocol by Segal.  ***Provider:*** MBCT was provided by therapists, who were not described further. | Fatigue;  Functional impairment;  Well-being. | CIS-Fatigue  SIP  DHDI |
| van der Meulen et al., 2013 | T = 49  C = 42 | Age = 60.40  %Female = 30%  %White = NR | Treatment as usual or standard care | Head and neck cancer diagnoses | Participants were receiving on-going treatment | Nurse Counseling and after Intervention (NUCAI)  ***Intervention Characteristics:*** The format of NUCAI consisted of patients receiving six in-person counseling sessions of forty-five to sixty minutes over the course of a year, provided by a trained nurse in the outpatient clinic. Sessions were always combined with the two-month medical check-ups received by the participants. NUCAI sessions focused on problem evaluation, reducing irrational thoughts, restructuring behavior, and behavioral activation.  ***Provider:*** NUCAI was provided by nurse interventionists. | Depression;  Functional health. | CES-D  EORTC |
| van Weert et al., 2010 | T = 76  C_1_ = 71  C_2_ = 62 | Age = 49.55  %Female = 86%  %White = NR | Active comparator  Waitlist or attention control | Multiple cancer diagnoses | Participants were in post-treatment survivorship | Cognitive-Behavioral Therapy (CBT)  ***Intervention Characteristics:*** The format of CBT consisted of group-based two-hour sessions, which were delivered in-person once a week, for twelve weeks. The content of CBT centered around training self-management skills based on the cognitive-behavioral problem-solving approach of Nezu et al. The approach was a psychotherapeutic, systematic, goal-oriented approach, aimed at finding effective and adaptive solutions to stressful problems and at changing dysfunctional cognition, emotions, and behaviors.  ***Provider:*** CBT was provided by two psychologists. | General fatigue;  Physical fatigue;  Mental fatigue. | MFI |
| Wells-Di Gregorio et al., 2019 | T = 17  C = 11 | Age = 56.54  %Female = 82%  %White = 93% | Waitlist or attention control | Cancer diagnosis in advanced stage | N/A | CBT-ACT (FOCUS) intervention  ***Intervention Characteristics:*** The format of FOCUS consisted of three one-and-ond-half-hour modules (two in-person one via DVD), delivered over six weeks. The content of FOCUS centered around CBT components including relaxation, sleep hygiene, sleep restriction, constructive worry, problem solving, SMART goal setting, activity pacing, and behavioral activation. FOCUS also included ACT processes, such as perspective taking and recognizing worry thoughts as thoughts, identifying values and acceptance of present-moment experience in the service of values, and committed actions.  ***Provider:*** FOCUS was provided by three postdoctoral fellows in psychosocial oncology. | Sleep difficulties;  Worry;  Hyperarousal;  Distress;  Fatigue;  Depression;  Anxiety. | WASO  PSWQ  IUS  ESS  STAI  CES-D  FSI  JSCS  IES-R |
| Zhang et al., 2019 | T = 20  C = 20 | Age = 52.8  %Female = 20%  %White = 0% | Treatment as usual or standard care | Gastrointestinal tract cancer diagnosis | Participants were in on-going treatment | Cognitive Behavioral Therapy (CBT)  ***Intervention Characteristics:*** The format of CBT consisted of individual cognitive behavioral therapy received in-person over the course of a five-week intervention. The content of CBT was divided and ordered sequentially by session: (1) week one cognitive reframing and cancer diet education were main themes; (2-4) week two through four focused on relaxation; and (5) week five focused on relaxation and exercise, including behavioral activation.  ***Provider:*** CBT was provided by three trained research assistants. | Symptom cluster;  Illness perception;  Anxiety;  Depression. | MDASI  B-IPQ  HADS |
| Abedini er al, 2021 | T = 20  C = 20 | Age = 12.12  %Female = 0.48  %White = NA | Treatment as usual or standard care | Pediatric cancer patients | Ongoing curative treatment | Mindfulness-based Cognitive Therapy for Children (MBCT-C)  ***Intervention Characteristics:*** The the program was compressed from 12 to 4 weeks. The intervention was conducted in Persian and handouts were translated into Persian by an experienced translator. A needs assessment pilot study was conducted with six participants (two small groups) to ascertain what modifications to the MBCT-C procedures and interventions might be required. Each session was 45 minutes. In response to the medical conditions of the participants, several activities were eliminated (mindful eating, yoga movements, and moving mindfully).  ***Provider:*** MBCT-C was provided by one trained therapist. | CBCL Internalizing; YSR Internalizing; CBCL Attention; YSR Attention | K-SADS-PL;  CBCL/6–18;  YSR/11-18 |
| Acevedo-Ibarra er al, 2019 | T = 40  C = 54 | Age = 54.82  %Female = 0.36  %White = NA | Treatment as usual or standard care | Colorectal cancer | Ongoing curative treatment | Cognitive Behavioral Stress Management (CBSM)  ***Intervention Characteristics:*** The CBSM intervention was modified from Penedo et al (208). The main adaptation was linguistic and cultural; the content was adapted to the needs of patients with colorectal cancer. The program consist of five two hours sessions. The components of the intervention were also preserved: cognitive behavioral techniques for stress, automatic thoughts, cognitive distortions, cognitive restructuring, coping, anger management, and assertive communication. And relaxation training using techniques like progressive muscular relaxation, diaphragmatic breathing, deep breathing, passive progressive muscular relaxation, guided imagination, and relaxation for healing and well‐being. The participant was given a homework manual, which included information about the session, monitoring sheets on the techniques used, and monitoring sheets on relaxation training.  ***Provider:*** CBSM was provided by trained facilitators. | Intrusive thoughts; Avoidance behavior; Hyperactivation; Distress; Optimism; Functioning; Symptoms; Total quality of life; Global health; Symptoms with a stoma; Symptoms without a stoma | IES; HADS; LOT-R;EORTC |
| Antoni et al, 2006 | T = 92  C = 107 | Age = 50.25  %Female = 1  %White = 0.68 | Waitlist or attention control | Breast Cancer | Newly diagnosed treatment | Cognitive Behavioral Stress Management (CBSM)  ***Intervention Characteristics:*** The Intervention—The closed, structured, manualized group intervention (Antoni, 2003) met weekly for ten 2-hr sessions. It interwove CBSM techniques with didactics, including in-session experiential exercises and out-of-session assignments (e.g., practicing relaxation). The intervention used group members and leaders as role models (for positive social comparisons and support), encouraged emotional expression, replaced doubt  appraisals with confidence (Beck & Emery, 1985), and honed skills in anxiety reduction (by  muscle relaxation and relaxing imagery; Bernstein & Borkovec, 1973) and skills in conflict resolution and emotional expression (via assertion training; Fensterheim & Baer, 1975).  ***Provider:*** CBSM was provided by by female postdoctoral fellows and advanced pre-doctoral trainees in clinical psychology. | Illness-Related Disruption of Interpersonal Activities; Positive states of mind; Positive emotions; Benefit finding; Positive lifestyle change; Specific Stress Management Skills and Nonspecific Effects | PSOM; SIP |
| Antoni et al, 2001 | T = 50  C = 50 | Age = 50.23  %Female = 1  %White = 0.74 | Waitlist or attention control | Early-Stage Breast Cancer | Ongoing curative treatment | Cognitive Behavioral Stress Management (CBSM)  ***Intervention Characteristics:*** The Intervention—The closed, structured, manualized group intervention (Antoni, 2003) met weekly for ten 2-hr sessions. The CBSM package thus included both problem-focused (e.g., active coping and planning) and emotion-focused (e.g., relaxation training, use of emotional support) coping strategies.  ***Provider:*** CBSM was provided by by postdoctoral fellows and advanced graduate students in clinical psychology who had been trained in the intervention and seminar protocols. | Mood disturbance; Depression; Thought intrusion and avoidance; Optimism; Benefit finding; Emotional processing | POMS; CES-D  ; IES; LOT-R |
| Beatty et al, 2010 | T = 25  C = 24 | Age = 55.2  %Female =1  %White = NA | Waitlist or attention control | Breast cancer | Newly diagnosed treatment | Self-help Workbook Intervention (SWI)  ***Intervention Characteristics:*** Treatment participants received a self-help workbook entitled “Finding your way: a workbook to help you cope with your breast cancer diagnosis and treatment”. Informational content was derived in consultation with consumers, cancer volunteers, and health professionals during a series of focus groups. Each chapter contained educational information on common medical and psychosocial issues; suggestions and work-sheets to address the issues; and survivors’ quotes. | Depression; Anxiety; Posttraumatic stress; Quality of life-Global; Quality of life- Body image; Anxious preoccupation; Helplessness/hopelessness; Cognitive avoidance | DASS; QOL |
| Beatty et al, 2016 | T = 30  C = 30 | Age = 52.73  %Female = 0.95  %White = NA | Waitlist or attention control | Multiple cancer diagnoses | Ongoing curative treatment | Cancer Coping Online (CCO)  ***Intervention Characteristics:*** CCO is a six module password-protected cognitive behavior therapy (CBT) programme, where each module is comprised of three key elements: (a) psycho-education, (b) CBT-based activities, including worksheets, quizzes, and relaxation and meditation exercises, and (c) written survivor testimonials and quotes. CCO also contains an online personal journal/blog and a resources section with links to reputable cancer-related organisations and other health websites. | Cancer distress; General distress; QOL-Global; QOL-Physical function; QOL-Role function; QOL-Emotional function; QOL-Social function; Helplessness/hopelessness; Anxious preoccupation; Cognitive avoidance | HRQOL; CCO; EORTC QLQ-C30; mini-MAC |
| Betiar et al, 2021 | T = 15  C = 15 | Age = 40.27  %Female = 0.48  %White = NA | Waitlist or attention control | Leukemia and Lymphoma | Ongoing curative treatment | Cognitive Behavioral Therapy (CBT)  ***Intervention Characteristics:*** The  ***Provider:*** CBT was provided by | Physical health; Mental health; HRQoL (total) | HRQOL |
| Bottomley et al, 1996 | T = 9  C = 14 | Age = 53.4  %Female =0.77  %White = NA | Active comparator | Multiple cancer diagnoses | Ongoing curative treatment | Cognitive Behavioral Therapy (CBT)  ***Intervention Characteristics:*** The CBT group intervention was modificd from the one developed by Moorey and Greer (1989), who used a more diadic approach. Each session lasted 90 minutes and ended on a positive note with a relaxation exercise. Example, the first three sessions focused on introductions and behavioral exercises (e.g., relaxation and scheduling of activities), and the fourth session focused on the CBT model and concepts. Sessions five through eight dealt with challenging dysfunctional thinking and learning coping skills. | Flightling Spirit; Anxious Preoccupation; Fatalism; Helplessness; Avoidance; Anxiety; Depression; Affective Social Support; Confident Social Support | HADS; MAC; BCSSS |
| Chambers et al, 2017 | T = 94  C = 95 | Age = 70.69  %Female = 0  %White = 0.66 | Treatment as usual or standard care | Proven metastatic and/or castration-resistant biochemical progression | Ongoing curative treatment | Mindfulness-based Cognitive Therapy (MBCT)  ***Intervention Characteristics:*** The program included eight group sessions delivered at weekly intervals. Each participant had an individual introductory telephone call with their facilitator to allow them to connect, prepare for the program, and enhance motivation. Participants unable to attend one or more sessions were offered a shortened, one-on-one, catch-up session with the facilitator to help rejoin the group at the next session. Each session was designed to run for 1.25 hours, with short meditations (15 minutes or less) to support group engagement and to alleviate practical concerns (eg, holding the telephone receiver). Workbooks included session plans so that participants could navigate telephone sessions more easily and interactive worksheets to keep group discussions on task. Peer interaction was directed at support for the learning of mindfulness skills and mutual support in facing the challenges of advanced PC. | Psychological distress; Cancer-specific distress; PSA anxiety; Quality of life; Benefit finding; Observing; Describing; Acting with awareness; Non-judging of inner experience; Non-reactivity to inner experience | BSI; IES; PSAA-MASPC |
| Chambers et al, 2014 | T = 178  C = 176 | Age = 55.38  %Female = 0.83  %White = NA | Active comparator | Multiple cancer diagnoses | Ongoing curative treatment | Cognitive Behavioral Therapy (CBT)  ***Intervention Characteristics:*** Participants in this arm were allocated to five sessions of telephone-based counseling from a psychologist that included, as core components, psychoeducation about the psychological impact of cancer, coping and stress management skills, problem solving, cognitive therapy, and enhancing support networks. The sessions followed principles of cognitive behavioral therapy with therapists applying flexibility in the order and depth of counseling in the core components to respond to the therapy goals of each participant. | BSI total; IES total; PTGI total | BSI-18; IES; PTGI |
| Chambers et al, 2018 | T = 22  C = 84 | Age = 57  %Female = 0.68  %White = NA | Active comparator | Multiple cancer diagnoses | NR | Web-Delivered Cognitive Behavioral Therapy (W-CBT)  ***Intervention Characteristics:*** Participants in the intervention arm were provided access to the Cancer Cope program, an online support program based on a 5-session telephone-based cognitive behavioral therapy intervention and modified  to include 6 cores covering: the cancer journey, understanding stress, managing worry, tackling problems, taking care (improving well-being), and moving forward. The cores consisted of educational information and expert videos from psychologists as well as stories and videos of 4 fictional characters on their cancer journey as a way to illustrate the different experiences of others. | BSI total; IES total; PTGI total; PGI total; AQOL total | BSI; IES; SCNSSF; PGI; AQOL |
| Chen et al, 2014 | T = 63  C = 56 | Age = 61.5  %Female =0.04  %White = 0 | Active comparator | Throat cancer | NR | Cognitive Behavioral Therapy (CBT)  ***Intervention Characteristics:*** The physician who provided CBT to all the patients in the CBT group was a licensed psychiatrist and counselor. CBT was delivered one-on-one; each session was documented in the out-patient records. Patients communicated with the clinicians by writing their responses. There were a total of 12 sessions over a period of eight weeks (one or two 1.0- to 1.5- hour sessions per week). The content of the sessions focused on discussion of patients’ understanding of their experiences of cancer treatment and on their interpretation of the difficulties they were experiencing in their daily lives. | SAS total; SDS total | SAS; SDS |
| Cousson-Gealie et al, 2011 | T = 22  C = 29 | Age = 53  %Female = 1  %White = NA | Treatment as usual or standard care | Breast cancer | Ongoing curative treatment | Cognitive Behavioral Therapy (CBT)  ***Intervention Protocol:***  the intervention comprised eight steps: discussion of most common irrational thoughts; self-efficacy and perceived control; effects of treatment on body; coping with illness and treatment; coping with anxiety (management of uncertainty); social support such as relations with family; expression of feelings (anger, sadness, anxiety); and discussion of personal goals and a summary. The format was identical to that of the Specific Intervention, eight sessions over one month. | Depression; Hospital Anxiety and Depression Scale; Social functioning; Cognitive functioning; Emotional functioning; Role functioning; Physical functioning; Quality of Life Questionnaire–C30-Global | SSQ; CLCS; CECS; MACS; HADS; QLQ-C30; |
| David et al, 2013 | T = 105  C = 81 | Age = 47.34  %Female = 0.6  %White = NA | Waitlist or attention control | Hematologic cancer | Ongoing curative treatment | Internet-based program for coping with cancer  ***Intervention Characteristics:***  The program developed for the study built on existing psychoeducational manuals for cancer patients that use elements of CBT. The goal was to communicate CBT-based techniques that can be of help when patients find themselves in situations of acute illness-related stress and which are effective in improving the momentary sense of well-being when it is compromised by anticipation of illness-related stress situations. Besides, Patients were permitted to communicate with one of two psychologists by web-based e-mail. Both had training and experience in psycho-oncology and the use of electronic media, with 3 years of prior experience counseling cancer patients by e-mail. | Fighting spirit (FS); Helpless/hopelessness (HH); Anxious preoccupation (AS); Fatalism (F); Avoidance (A); BSI - GSI | MAC; BSI; ZUF-8 |
| Davis et al, 1986 | T = 5  C = 7 | Age = 50.54  %Female = 1  %White = NA | Active comparator | Breast cancer | Newly diagnosed treatment | Cognitive Behavioral Therapy (CBT)  ***Intervention Characteristics:*** The Biofeedback.-Training was conducted over 8 wk. in 10 biweekly, 45-min. sessions followed by three once-weekly sessions.  ***Provider:*** CBT was provided by a Bachelor of Social Work, degree and 6 yr. of clinical experience. Her work was supervised by the principal investigator, a PhD-level clinical psychologist with experience in biofeedback, cognitive therapy, and therapist training/supervision. | Cortisol production |  |
| Desautels et al, 2018 | T = 25  C = 26 | Age = 57.1  %Female =1  %White = 1 | Active comparator | Breast Cancer | Ongoing curative treatment | Cognitive Therapy (CBT)  ***Intervention Characteristics:*** CT was administered individually by a doctoral-level student in clinical psychology and involved 8 weekly sessions of approximately 60 min. The treatment was manualized, and the protocol was based on Beck’s CT for depression. | HADS-D total; BDI-II total; HDRS total | HADS-D; BDI-II; HDRS |
| Diaz et al, 2021 | T = 23  C = 14 | Age = 53.65  %Female = 1  %White = 0.45 | Waitlist or attention control;  Active comparator | Breast  cancer | Ongoing curative treatment | Cognitive Behavioral Therapy (CBT)  ***Intervention Characteristics:*** The CBT condition was derived from the CBT components of the 10-week CBSM manualized intervention (Antoni, 2003). Theory. Session content included thought monitoring, cognitive restructuring, adaptive coping skills, communication skills, and social network building. Weekly home exercises were assigned to emphasize session material. | NF-κB DNA binding; Circulating pro-inflammatory cytokines and s100A8/A9; Phenotype analysis; IES-R total; ABS-NA total; PSMS total | IES-R; ABS-NA; PSMS |
| do Camo et al, 2017 | T = 19  C = 22 | Age = 53.12  %Female = 0.65  %White = 0.59 | Treatment as usual or standard care;  Active comparator | Multiple cancer diagnoses | Palliative treatment (including transition from curative to palliative) | Cognitive Behavioral Therapy (CBT)  ***Intervention Characteristics:*** The intervention consisted of five weekly individual sessions performed in a room fit to receive the participants. The psychosocial intervention was based on CBT techniques; the protocol was developed based on the session structure method formulated by previous researchers. | ESAS- Anxiety; ESAS-Depression; ESAS- Emotional domain; EORTC QLQC-15Pal Total; EORTC QLQC-15Pal Emotional domain; HADS-Depression; HADS-Anxiety; PHQ-9 | HADS-A; HADS-D; PHQ-9; ESAS; QLQ-C15 |
| Downe-Wamboldt et al, 2007 | T = 76  C = 73 | Age = 62.3  %Female =0.6  %White = 0.91 | Treatment as usual or standard care | Multiple cancer diagnoses | Mixed | Telephone problem-solving counseling intervention (TPCI)  ***Intervention Characteristics:*** In addition to the usual cancer care, a series of telephone problem-solving counseling sessions were offered at the participant’s convenience during a 3-month interval starting within 1 week after consent was obtained (usually 2-3 months after surgery). Participants were encouraged to identify alternate solutions and evaluate the consequences of the alternative solutions. The telephone nurse counselor facilitated the patient’s selection of the most effective solution and verified the efficacy or outcomes of the solutions using a study protocol.  ***Provider:*** Two baccalaureate registered nurses (RN) were recruited and further trained in problem-solving counseling by a doctoral-prepared nurse therapist with 25 years’ experience. | Confrontive coping; Evasive coping; Optimistic coping; Fatalistic coping; Emotive coping; Palliative coping; Supportant coping; Self-reliant coping | CES-D; PAIS-SR |
| DuHamel et al, 2010 | T = 47  C = 34 | Age = 51.01  %Female = 0.51  %White = 0.81 | Treatment as usual or standard care  Waitlist or attention control  Active comparator? | Survived from Hematopoietic stem-cell transplantation | Post-treatment survivorship | Telephone-Administered Cognitive-Behavioral Therapy (T-CBT)  ***Intervention Characteristics:*** The 10-session manualized T-CBT intervention was delivered during a period of 10 to 16 weeks. Team members were supervised individually and as a group by senior CBT clinicians throughout the study. The  intervention included education regarding illness-related PTSD symptoms and CBT, self-monitoring and alteration of maladaptive beliefs, guided exposure to cues associated with PTSD symptoms, enhancement of social support through training in communication skills, and relaxation training. | Total PCL-C; PTSD diagnosis; Global BSI distress; BSI depression | OCI-C; BSI; The Clinician-  Administered PTSD Scale for DSM-IV |
| Evans et al, 1995 | T = 27  C = 24 | Age = 53.92  %Female = 0.35  %White = 0.6 | Active comparator | Multiple cancer diagnoses | Mixed | Cognitive Behavioral Therapy (CBT)  ***Intervention Characteristics:*** Each session had a skill training theme, and the sessions involved teaching participants coping skills, group discussion of potential uses and benefits of the skills, and weekly review of success in implementation. Skill areas included modification of cognitions that exacerbate anxiety or depression, progressive muscle relaxation, and establishment of a network of supportive relationships. The questions, concerns, and problems of participants in implementation of change were handled from a problem-solving perspective. Sessions also included at-home practice assignments. | CES-D total; SCL-90-R-Global; SCL-90-R-Deprssion; SCL-90-R-Somatization; SCL-90-R-Hostility; SCL-90-R-Phobia; SCL-90-R-Anxiety; Social support | CES-D; SPS; SCL-90-R; MHLC |
| Fadeal et al, 2011 | T = 32  C = 40 | Age = 43.84  %Female = 1  %White = 0 | Waitlist or attention control | Breast cancer | Ongoing curative treatment | Ellis rational emotive behavior therapy (REBT) ***Intervention Characteristics:*** This intervention conducted for 6 sessions during 3 weeks. Consultation contained logical treatment, training of muscle relaxation, adaptive skills, and problem solving. The patients should have done their tasks in their home between the sessions. Each session lasted 90 minutes and each group was comprised of 10-11 patients. | EORTC total | BIS; EORTC |
| Ferguson et al, 2012 | T = 19  C = 21 | Age = 50.28  %Female = 1  %White = 0.975 | Waitlist or attention control | Multiple cancer diagnoses | Post-treatment survivorship | Memory and Attention Adaptation Training (MAAT) ***Intervention Characteristics:*** This study consisted of four biweekly individual office visits 30–50 min in duration with phone contacts between visits. In each visit, participants reviewed present findings and knowledge about cognitive effects of chemotherapy, learned how to self-monitor and identify ‘at-risk’ situations where cognitive failures are likely to occur (self-awareness training), and learned and rehearsed compensatory strategies to prevent or lessen negative consequences of cognitive failure (e.g. emotional distress or daily occupational or social role performance). Participants applied the strategies in daily situations to foster adaptive learning and telephone contacts between visits were intended to reinforce use of new behaviors or modify the strategy to enhance effectiveness. | MASQ total score; Quality of life–CS psychological well-being; Quality of life—CS spiritual well-being; Quality of life—CS physical well-being; Quality of life—CS social well-being; CVLT-II-Total; Digit symbol–coding; Color word trial; Color word switching trial; Trail making number–letter trial | MASQ; QOL-CS; CES-D; STAI; Treatment Satisfaction |
| Foley et al, 2010 | T = 55  C = 60 | Age = 55.18  %Female = 0.77  %White = NA | Waitlist or attention control | Multiple cancer diagnoses | NR | Mindfulness-based Cognitive Therapy (MBCT) ***Intervention Characteristics:*** The program was delivered in eight weekly 2-hr sessions with a group of 8 –12 individuals. Participants were provided with handouts containing information pertinent to each session, including suggested reading from Jon Kabat-Zinn’s Full Catastrophe Living (1990). | HAM-D total; HAM-A total; FMI total; DASS total; FACT-G total | HAM-D; HAM-A; FMI; DASS; FACT-G |
| Garland et al, 2015 | T = 40  C = 32 | Age = 59.44  %Female = 0.72  %White = NA | Active comparator | Insomnia | Post-treatment survivorship | Cognitive Behavioral Therapy for Insomnia (CBT-I)  ***Intervention Characteristics:*** The CBT-I program followed the format of previously  published CBT-I trials in cancer patients.51,52 It is delivered to groups of 6–10 individuals over the course of eight weekly 90-minute sessions, for a total of 12 contact hours. Participants are guided through the implementation of stimulus control, sleep restriction, and relaxation training techniques. They are also taught how to apply cognitive strategies to challenge dysfunctional sleep beliefs. Proper sleep hygiene is also reviewed. | ISI total; DBAS Expectations; Worry; Consequences; Medication; FFMQ total | ISI; FFMQ |
| Gaston-Johansson et al, 2013 | T = 38  C = 35 | Age = NA  %Female = 1  %White = 0.88 | Treatment as usual or standard care | Breast cancer | Ongoing curative treatment | Comprehensive coping strategy program (CCSP) ***Intervention Characteristics:*** The CCSP intervention is a multimodal coping strategies approach consisting of four components: (i) preparatory education, (ii) cognitive restructuring, (iii) coping skills enhancement, and (iv) relaxation with guided imagery. Specific content areas are matched with the most appropriate teaching strategy (e.g., presentation, power points, handouts, and relaxation tape) and with the needs of the learner (e.g., communication style and computer or telephone reinforcement). | State-anxiety; Trait-Anxiety; Depression; Ignoring pain; Coping self-statements; Reinterpretation; Diverting attention; Praying; Behavioral adaptation; Avoidance of catastrophizing; Overall coping | QOLI-CV; BDI; STAI; CSQ |
| Graboyes et al, 2022 | T = 20  C = 20 | Age = 63  %Female = 0.61  %White = 84 | Waitlist or attention control | Head and Neck Cancer | Post-treatment survivorship | Brief Tele–Cognitive Behavioral Treatment (BRIGHT)  ***Intervention Characteristics:*** BRIGHT is a manualized, theory-based23-26CBT consisting of 5  weekly 60-minute sessions delivered one-on-one by a licensed clinical psychologist via video telemedicine platform, as previously described.17 BRIGHT session topics include (1) psychoeducation about the cognitive model of body image; (2) self-monitoring about thoughts, feelings, and body image  behaviors; (3) cognitive restructuring to identify and challenge unhelpful automatic HNC-related body image thoughts; (4) positive body image coping strategies; and (5) maintenance and relapse prevention. Patients receive a BRIGHT workbook  with objectives, educational materials, in-session exercises, and weekly homework. | BID total | BID |
| Greer et al, 2012 | T = 20  C = 20 | Age = 55.9  %Female =0.7  %White = 0.95 | Waitlist or attention control | Multiple cancer diagnoses | Mixed | Cognitive Behavioral Therapy (CBT)  ***Intervention Characteristics:*** All study therapists and independent evaluators received extensive training from the principal investigator (PI), a licensed clinical psychologist, regarding the intervention and evaluation procedures prior to meeting with participants. The sessions occurred weekly whenever possible. The principal investigator (a licensed clinical psychologist) and four clinical psychology fellows with at least 4 years supervised experience in delivering CBT served as study therapists. The duration of treatment was brief (approximately 2 months) by design, considering the progressive morbidity of the study population. | HAM-A total; CGI - total; HADS-A total; IES total | HAM-A; CGI; HADS-A; IES |
| Groarke et al, 2013 | T = 87  C = 92 | Age = 53.7  %Female =1  %White = NA | Active comparator | Breast cancer | Post-treatment survivorship | Cognitive-behavioral stress management (CBSM)  ***Intervention Characteristics:*** The team members met on the university campus, in groups of 8–12 for 3hr per week for 5 weeks. The group was facilitated by an experienced clinical psychologist. To achieve reliability, this trial utilized the same facilitator throughout and used a session-by-session manual for the eight intervention cohorts. | PSS total: IES total; LOT total; HADS total; SLQ total | PSS: IES; LOT; HADS; SLQ |
| Ham et al, 2019 | T = 21  C = 21 | Age = 44.1  %Female =0.86  %White = 0 | Waitlist or attention control | Multiple cancer diagnoses | NR | Mobile-application-based cognitive behavioral therapy program (HARUToday)  ***Intervention Characteristics:*** An app-based CBT program, HARUToday, which was developed by the authors for the purpose of this study, was provided to the participants in the intervention group. HARUToday is composed of five zones: (1) psycho-education, (2) behavioral activation, (3) relaxation training, (4) cognitive restructuring, and (5) problem-solving. The program was developed in the form of contents-based e-learning that minimizes text and takes advantage of visual and auditory examples, taking into account the age range and interests of the participants. | BDI-II; STAI-State; STAI-Trait; STAI-SF-36; STAI-SAS | SDI-II; STAI |
| Heinrichs et al,2012 | T = 38  C = 34 | Age = 52.2  %Female =1  %White = NA | Waitlist or attention control | Multiple cancer diagnoses | Ongoing curative treatment | Relationship Skills Program—Side by Side  ***Intervention Characteristics:*** The Side by Side intervention consists of four bi-weekly, face-to-face sessions with a therapist in the couple’s home, each session lasting 120 min (intervention time in sum: approx. 8 h). The intervention components included in Side by Side are all adapted from empirically supported cognitive-behavioral interventions and maintain a focus on the couple | QSC-R total; PGI total; QMI total; PQ total; DCI total | QSC-R; PGI; QMI; PQ; DCI |
| Herschbach et al, 2010 | T = 91  C = 83 | Age = 53.7  %Female = 0.83  %White = NA | Active comparator | Multiple cancer diagnoses | NR | Cognitive Behavioral Therapy (CBT)  ***Intervention Characteristics:*** CBT focused on fear of progression and was based on the principles of cognitive behavior therapy. The groups were led by 11 psychotherapists. Requirements were a minimum of 3 years psychotherapeutic experience and/or that the psychologists were in the final phase of their psychotherapy training. | FoP-Q total score; affective reaction; partner; work; loss of autonomy; coping with fears | FoP-Q |
| Hyland et al, 2022 | T = 22  C = 14 | Age = 56  %Female = 0.47  %White = NA | Waitlist or attention control | Chronic Myeloid Leukemia | Ongoing curative treatment | Cognitive behavioral therapy for targeted-therapy related fatigue (CBT-TTF)  ***Intervention Characteristics:*** Patients randomized to CBT-TTF met with a study therapist for an initial in-person session at Moffitt Cancer Center. The intervention was delivered by the study therapist over 18 weeks, and sessions took place approximately weekly. CBT-TTF consists of six modules targeting factors thought to perpetuate targeted therapy-related fatigue: (a) a disturbed sleep/wake cycle, (b) dysregulated activity patterns, (c)maladaptive cognitions about fatigue and cancer, (d) insufficient processing of cancer diagnosis and treatment, (e) inadequate social support and interactions, and (f) heightened fear of cancer progression. | Sleep; Mental activity; Physical activity; social activity; self-efficacy; fatigue catastrophizing; helpless; focusing on symptoms; avoidance; social; fear of progression; intrusive thoughts | FACIT-F; SIP-SR; SES; FCS; ICQ; IMQ; IES-I; IES-A; SSL |
| Kingston et al, 2015 | T = 7  C = 6 | Age = 50.07  %Female = 0.63  %White = 1 | Active comparator | Multiple cancer diagnoses | Post-treatment survivorship | Mindfulness-based cognitive therapy (MBCT)  ***Intervention Characteristics:*** The participants received an 8-week programme of MBCT. Cancer specific adjustments were made to the MBCT programme in the cognitive sections of the sessions, with particular attention to cancer-specific rumination and physical problems such as cancer-related fatigue. The mindful movement exercises contained in the pro-gramme were modified in consultation with the hospital physiotherapy department to be safe in individuals who might have had recent surgery, or who had ongoing physical problems secondary to their cancer.  ***Provider:*** each. The sessions were carried out by two experienced therapists, one a Senior Registrar in Psychological Medicine, the other a Senior Clinical Psychologist. Both are accredited by the British Association of Behavioral and Cognitive Therapists and have received formal training in MBCT. | KIMS observe; KIMS describe; KIMS act; KIMS acceptance; KIMS total; Self-kindness; Self-judgement; Common humanity; Isolation; Mindfulness; Over-identification; HADS depression; POMS depression | HADS; POMS; BSI; WHO-5; KIMS; SCS |
| Kissane et al, 2003 | T = 154  C = 149 | Age = 46.3  %Female =1  %White = NA | Waitlist or attention control | Breast Cancer | Ongoing curative treatment | Cognitive-existential group therapy (CEGT)  ***Intervention Characteristics:*** This manualised group therapy had 6 goals: promoting a supportive environment; facilitating grief over losses; reframing negative thoughts; enhancing problem solving and coping; fostering hope; and examining priorities for the future. Groups, comprising 6–8 patients and 2 therapists (one always a woman) met for 20 weekly sessions, each lasting 90 min. Preparation of each patient individually in a standardised and manualized pretreatment session was an intrinsic feature to reduce dropouts. | MILP total; ABS total; MAC total; FAD total | MILP; ABS; MAC; FAD |
| Klosky et al, 2004 | T = 41  C = 38 | Age = 4.2  %Female = NA  %White = NA | Waitlist or attention control | Multiple cancer diagnoses | NR | Radiation therapy (RT)  ***Intervention Characteristics:*** Children assigned to the IG received a cognitive–behavioral intervention package (STARBRIGHT Hospital  Pals) that included exposure to an interactive animatronic plush Barney character, an educational video including filmed modeling, and passive auditory distraction via Barney-narrated stories delivered during the RT procedure. | HR grade | STAI |
| Korstjens e al,2011 | T = 76  C = 71 | Age = 48.8  %Female =0.83  %White = NA | Active comparator | Multiple cancer diagnoses | Post-treatment survivorship | Cognitive Behavioral Therapy (CBT)  ***Intervention Characteristics:*** The intervention was a PT programme (twice weekly, 2 h sessions) plus a CBT (weekly, 2 h sessions). PT was guided by two physical therapists and CBT was guided by a psychologist and a nurse, physical therapist or social worker. The experience of physical therapists in cancer rehabilitation ranged from 2.5 to 6.3 years (median 5.1 years) and CBT therapists were working in cancer rehabilitation between 2.4 and 11.3 years (median 4.4 years). All therapists received group training to apply the standardised self-management protocols: PT therapists for 1 day; CBT therapists for 2 days. | Anxiety; Depression; Negative problem orientation; positive problem orientation; Raditional problem solving; impulsive style; avoidance style | SPSI-R; HADS |
| Lechner et al, 2014 | T = 57  C = 57 | Age = 51.1  %Female =1  %White = 0 | Active comparator | Breast Cancer | Post-treatment survivorship | Community-Based Stress Management Intervention (CBSM)  ***Intervention Characteristics:*** Groups were led by a licensed clinical psychologist who was also a black woman. Participants received a workbook that contained the session content, short out-of-session exercises, and the content of the CW condition needs). Lechner (2016) provides the details of the process of adapting the CBSM intervention into a culturally targeted version. | PSS total; CESD total; CRTI total; PMSSV total; FACT total | PSS; CESD; CRTI; PMSSV; FACT |
| Mann et al, 2012 | T = 15  C = 17 | Age = 53.61  %Female = 1  %White = 0.85 | Treatment as usual or standard care | Breast Cancer | Mixed | Cognitive Behavioral Therapy (CBT)  ***Intervention Characteristics:*** The intervention consisted 90 min session every week for 6 weeks. A treatment manual was produced in advance of the study, which contained detailed session content, presentation slides and handouts, and notes for facilitators. A clinical psychologist was trained to deliver the sessions with the help of an assistant (five assistants took part over the course of the study). All sessions were audio taped, then  10% were randomly selected (with a computer-generated random number sequence) and a psychologist (MSH) experienced in cognitive behavioral therapy for HFNS, rated them for adherence to the treatment manual, by indicating on coding sheets the extent to which the group leader covered each topic. | HNFS total | HFNS |
| Manne et al, 2007 | T = 12  C = 120 | Age = 50.01  %Female = 1  %White = 0.895 | Waitlist or attention control;  Active comparator | Gynecological Cancers | Ongoing curative treatment | C Communication-enhancing intervention (CCI) and supportive counseling (SC)  ***Intervention Characteristics:*** Both intervention consisted of six hour-long individual sessions and one telephone booster session that took place 1 week after the sixth session. | Beck Depression Inventory total; Impact of Event Scale total | BDI; IES; CREY; EEQ; |
| Manne et al, 2017 | T = 118  C = 118 | Age = 55.3  %Female =1  %White = 0.79 | Waitlist or attention control;  Active comparator | Gynecological cancer | Newly diagnosed treatment | Communication-enhancing intervention (CCI) and supportive counseling (SC)  ***Intervention Characteristics:*** Both intervention consisted of seven hour-long individual weekly sessions and one telephone booster session two to three weeks after session seven. Therapists comprised social workers, master-level or doctoral-level psychologists, or psychiatrists who were practicing in the community or employees of each cancer center. They had between one and 34 years of therapy experience. Each therapist underwent 6 h of training in the manual-based CCI or SC interventions. | Depression; Cancer-specific distress; Fear of recurrence; Emotional well-being | BDI; IES; FACT-G; EEQ; CARES |
| Marchioro et al,1996 | T = 18  C = 18 | Age = 52  %Female =1  %White = NA | Treatment as usual or | Breast Cancer | Ongoing curative treatment | Cognitive Behavioral Therapy (CBT)  ***Intervention Characteristics:*** The psychological intervention, administered to Group A, consisted of weekly 50 minute individual cognitive psycho-therapy sessions with a psychologist. All sessions were performed by the same psychologist. Group B patients underwent only self-appraisal questionnaires and personality testing during a schedule oncological examination, but they did not receive any psychological intervention. | IIQ total; BDI total; FLIC total | IIQ; BDI; FLIC |
| McKierman et al, 2010 | T = 36  C = 33 | Age = 50.72  %Female = 1  %White = NA | Treatment as usual or standard care | Breast Cancer | Post-treatment survivorship | Cognitive Behavioral Therapy (CBT)  ***Intervention Protocol:***  Session 1: Coping- Identifying strategies for coping with the experience of cancer.  Session 2: Relaxation- Introduction to the cognitive behavioral theory model: practicing relaxation and activity scheduling.  Session 3: Communication- Understanding the value of effective communication with health care professionals, friends and family.  Session 4: Though monitoring-Recognizing cognitive distortions and monitoring negative automatic thoughts.  Session 5: Assumptions & beliefs-Examining the influence of assumptions and core beliefs in adjusting to the cancer experience.  Session 6: Problem solving &goal setting-A systematic approach to problem solving: defining values and goals. | Adaptive  Coping; Maladaptive  Coping; Quality of  Life; Total Mood  Disturbance | COPE; PPGQ; SUQ |
| Merckaert et al, 2017 | T = 82  C = 77 | Age = 50.6  %Female =1  %White = 0.8 | Treatment as usual or standard care | Breast Cancer | Post-treatment survivorship | Single‐component and Multiple‐component Group Interventions (SGI & MGI)  ***Intervention Characteristics:*** Both interventions were delivered in a closed‐group format (in groups of 6 participants) and comprised 15 weekly 120‐minute sessions occurring within a 6‐month period. Clinical psychologists followed a session‐by‐session structured manual. Audio and video recordings of all sessions were collected to be used in these intevisions if needed. Basic skills acquired by psychologists during their university training were sufficient for the SGI while a specific training in hypnosis and cognitive‐behavioral techniques was necessary for the MGI. All the psychologists had at least 1 year of experience in cancer care. | HADS-Anxiety; HADS; Distress; HADS-Depression; EAR-Anxiety level; FCRI-Triggers; FCRI-Severity; Psychological distress; Functioning impairments ; Coping strategies; Reassurance; Insight; Fighting spirit; Anxious preoccupation; Helplessness/hopelessness; Fatalism; Avoidance | VAS; RSQ; HADS; MACS; FCRI; EAR |
| Mishel et al, 2005 | T = 244  C = 265 | Age = 64  %Female1 =  %White = 0.71 | Treatment as usual or standard care | Breast Cancer | Post-treatment survivorship | Theoretically based uncertainty management intervention delivered  ***Intervention Characteristics:*** Using a standardized protocol, intervention nurses guided women through the intervention over the course of four weekly telephone calls. Nurses instructed the women to use these skills when confronting a trigger of fear of recurrence or when anticipating such a trigger. During each telephone session, nurses encouraged women to practice the skills by listening to the audiotapes and using the manual during the following week. | Cognitive  reframing, problem solving, cancer knowledge,  social support satisfaction, and patient–provider  communication; CSQ total | CSQ; POMS-SF |
| Mokrivala et al, 2022 | T = 18  C = 19 | Age = 48.64  %Female = 1  %White = NA | Waitlist or attention control | Breast Cancer | Post-treatment survivorship | Gestalt Therapy in Combination with Cognitive Behavioral Therapy (GT-CBT)  ***Intervention Protocol:***  1 Introduction, goals, expectations, future sessions, breast cancer and psychotherapy effectiveness, GT and CBT effectiveness for cancer patients  2 The unfinished works listed (unfinished works technique), the automatic thoughts identified through worksheets (first step of cognitive restructuring)  3 Challenge with automatic thoughts identified through worksheets (second step of cognitive restructuring), the technique of inversed verb game used while sharing personal experience in the group: “I take responsibility of feeling...”  4 Challenging automatic thoughts replaced through worksheets (third step of cognitive restructuring), the inverted role game was introduced and taught  5 Three steps of cognitive restructuring were reviewed, and group discussion about the steps and experiences in the last sessions  6 The projecting game was taught by members and the relaxation technique was introduced and trained  7 Desensitization of stress was taught and the hot chair technique was used for voluntaries  8 The sessions and techniques were reviewed, providing feedback, answering questions, and performing post-test | Pain intensity; Hope | MHS; VAS |
| Moorey et al, 1998 | T = 25  C = 22 | Age = 51  %Female0.7447 =  %White = NA | Active comparator | Multiple cancer diagnoses | NR | Adjuvant Psychological Therapy(APT)  ***Intervention Characteristics:*** In this study, APT was administered as 8 weekly sessions with the patient, and the spouse where appropriate. The treatment was carried out by therapists, SM and SG. These therapists had developed APT and so were expert in this approach. They had experience of using the techniques of the comparison therapy in their practice of supportive psychotherapy as psychiatrists. | HADS total; MACS total; SSTAI total; CCS total; BDI total; SDPS total | HADS; MACS; SSTAI; CCS; BDI; SDPS |
| Murphy et al, 2020 | T = 53  C = 61 | Age = 53.29  %Female = 0.89  %White = NA | Treatment as usual or standard care | Multiple cancer diagnoses | Ongoing curative treatment | Cognitive Behavioral Therapy (i-CBT)  ***Intervention Characteristics:*** The iCBT program, named “iCanADAPT Early” consisted of an online self‐managed but clinician supervised, 16‐week, eight‐lesson program with general and cancer‐specific cognitive‐behavioral (CBT) skills. It was adapted from a proven trans-diagnostic program.11 There are four components: (a) The compulsory “lesson” i.e., content, presented in simplified comic form (low literacy requirement); (b) A noncompulsory supplementary extra resources section post lesson (moderate literacy requirement); (c) A downloadable “lesson summary” outlining the CBT practice and homework exercises; and (d) an audio‐visual component comprising of an introductory short video and two CDs (one outlined relaxation strategies, the other basic mindfulness practices). | DSM diagnosis; Depression/anxiety before cancer (self‐report); HADS‐Total; K‐10; FCRI‐Total; FACT‐G‐Total; Social/Family well‐being; Emotional well‐being; Functional well‐being; Physical well‐being | HADS; FCRI; FACT-G; CESQ; BDI-II; PHQ9 |
| Onyechi et al, 2016 | T = 16  C = 16 | Age = 48.33  %Female = 0.875  %White = 0 | Treatment as usual or standard care | Multiple cancer diagnoses | Mixed | Rational emotive hospice care therapy (REHCT) ***Intervention Characteristics:*** The manual of this intervention is based on a cognitive behavioral approach, incorporating some of Albert Ellis’s quotes to aid in disputing participants’ problematic assumptions and to motivate participants to change their dysfunctional emotions and thoughts around cancer. The manual indicates treatment sessions anchored on several treatment strategies, including  cognitive restructuring, confrontation, therapeutic alliance, acceptance, Socratic dialogue, reframing,  metaphors, worksheets, and motivation, which form the standard of care for the patients and caregivers in the treatment group. | CPFCAQ total; DAQ total | CPFCAQ; DAQ |
| Peoles et al, 2017 | T = 23  C = 24 | Age = 56  %Female0.88 =  %White = 0.9 | Active comparator | Multiple cancer diagnoses | Post-treatment survivorship | Cognitive behavioral therapy for insomnia (CBT-I)  ***Intervention Characteristics:*** The CBT-I intervention was 7 weeks long, was provided on an individual basis by therapists trained in CBT-I, and followed a published treatment manual. There were 7 individual weekly CBT-I sessions, with sessions 1, 2, and 4 (30–60 min) conducted in person, and sessions 3, 5, 6, and 7 (15–30 min) conducted over the telephone. Armodafinil is a CNS stimulant and the R-enantiomer of modafinil. | FACT-G; ISI | FACT-G; ISI |
| Phillips et al, 2011 | T = 65  C = 63 | Age = 49.69  %Female = 1  %White = 0.7 | Waitlist or attention control | Non-Metastatic Breast Cancer | NR | Cognitive behavioral stress management (CBSM)  ***Intervention Characteristics:*** Participants randomized to the CBSM intervention (n = 65) met in groups of three to nine patients for 10 weekly 2-h sessions. Groups were led by two female facilitators (one pre-doctoral and one post-doctoral) trained in the manualized CBSM intervention. The manualized intervention (Antoni 2003) was developed following a biobehavioral conceptualization of the breast cancer experience (Andersen et al. 1994). | Cortisol; MOCS-relaxation; MOCS-reappraisal | MOCS |
| Poggi et al, 2009 | T = 17023  C = | Age = 9.41  %Female =0.3  %White = NA | Treatment as usual or standard care | Brain tumor | Post-treatment survivorship | Cognitive Behavioral Therapy (CBT)  ***Intervention Characteristics:*** Treatment lasted from 4 to 8 months with 2/3 weekly individual sessions lasting 45–60 min. A weekly session for parents was also planned. The CBT for paediatric cancer patients include both cognitive techniques (cognitive mediation and analysis, and change in dysfunctional cognitive schemata) and behavioural techniques (positive and negative reinforcement, extinction, response cost, token economy, contingency contract, time-out, relaxation training, modelling, shaping, prompting, fading, systematic desensitising, relaxation training) | Daily living skills; Communication; Social skills; Motor skills; Withdrawn; Somatic complaints; Anxiety/Depression; Social problems; Thought problems; Attention problems; Delinquent behavior; Aggressive behavior; Internalising; Externalising | VABS; WPPSI-R; WISC-R; WAIS-R |
| Richardson et al, 2017 | T = 17  C = 16 | Age = NA  %Female 0.23=  %White = 0.28 | Treatment as usual or standard care | Head and Neck  Cancer | Ongoing curative treatment | Cognitive Behavioral Therapy (CBT)  ***Intervention Characteristics:*** The intervention consisted of three 60-min face-to-face sessions with a health psychologist. A 30-min follow-up phone call also took place approximately 3 weeks after the final session. Intervention sessions took place at hospital or at patients’ homes, depending on their personal preference. An educational manual of materials entitled “Head and Neck Cancer: A Guide for Patients and their Family  Members” was developed as part of this study and provided to patients as a supplement to intervention sessions. | Total HRQL; Physical; Social; Emotional; Functional; Additional | FACT-H&N; GHQ-12; Brief IPQ |
| Serfaty et al, 2000 | T = 115  C = 115 | Age = 59.5  %Female =0.66  %White = 0.73 | Treatment as usual or standard care | Multiple cancer diagnoses | End of life care  Mixed | Cognitive Behavioral Therapy (CBT)  ***Provider:*** CBT therapists attended a 1-day course on how to use the manual and adapt their standard CBT work for people with advanced cancer. The study used only high-level (level 3) ‘high-intensity therapists’ with at least 2 years postgraduate diploma experience in CBT. | BDI-II total; PHQ-9 total; EQ-5D total; ECOG-PS total | BDI-II; PHQ-9; EQ-5D; ECOG-PS |
| Serfaty et al, 2012 | T = 20  C = 19 | Age = 52.5  %Female =0.79  %White = 0.795 | Active comparator | Multiple cancer diagnoses | NR | Cognitive Behavioral Therapy (CBT)  ***Intervention Characteristics:*** The intervention was operationalised using a checklist covering the following areas: (i) The effects of physical illness: the impact of the illness, beliefs and expectations about the illness, their plans and hopes for care as the disease advances. (ii) The emotional impact of the disease and coping strategies: the relationship between emotions, physical symptoms and disability caused by the disease and concerns about the patients ability to cope, their loss of control, and preparedness to accept help and discuss issues around dying. (iii) The social impact: impact of disease and mood on behavior and ability to fulfil roles, impact of disease on loved ones. (iv) Spiritual and existential issues: discussion of the meaning of the illness, suicide/euthanasia issues, the potential for spiritual reconciliations, absolutions forgiveness and acceptance of unfinished business.  ***Provider:*** Therapists delivering the CBT were accredited with the British Association of Behavioral and Cognitive Psycho-therapists and were supervised (by MS) weekly. | POMS total | HADS; POMS |
| Solkoglu et al, 2023 | T = 49  C = 23 | Age = 47.8  %Female =1  %White = NA | Treatment as usual or standard care | Breast cancer | Post-treatment survivorship | Cognitive Behavioral Therapy (CBT)  ***Intervention Characteristics:*** The iCBT programme is a 7‐module intervention based on a transdiagnostic approach for depression and anxiety provided through the SilverCloud platform. The intervention consisted of various tools such as quizzes, goal setting, mood monitoring, activity scheduling, thoughts‐feelings‐behaviours cycle, worry tree, and relaxation exercises. Intervention participants were assigned a supporter, who provided guidance and post‐session feedback on a weekly basis over 8 weeks. There were 10 supporters, who were graduate psychologists doing their masters. Supporters received training on the delivery of online support and weekly supervision provided by SA and DH. | HADS-T; EORTC-QLQ; CWC; Active coping; Avoidant coping; MOS-SSS | HADS-T; EORTC-QLQ; CWC; Brief COPE; MOS-SSS |
| Speer, 1987 | T = 15  C = 15 | Age = 48.85  %Female = 0.67  %White = 1 | Waitlist or attention control | Multiple cancer diagnoses | Ongoing curative treatment | Cognitive Behavioral Therapy (CBT)  ***Intervention Characteristics:*** Patients were invited to attend weekly, one-hour, individual sessions over a 10-weeek period. Patients were taught skills in assertiveness. All sessions were conducted by the investigator, under the supervision of a physician, and in consultation with a licensed psychologist. | Depression; Distress | BDI; SDS |
| Stagel et al, 2015 | T = 120  C = 120 | Age = 56.71  %Female = 1  %White = 0.7154 | Waitlist or attention control | Breast Cancer | NR | Cognitive–behavioral stress management (CBSM)  ***Intervention Characteristics:*** The CBSM intervention was a 10-week group based intervention for women undergoing BCa treatment (Antoni, 2003). CBSM incorporates cognitive-behavioral therapy (e.g., cognitive reframing, assertiveness training) and relaxation training (e.g., progressive muscle relaxation) to decrease stress and negative mood. | Depression | CES-D |
| Taeidi et al, 2018 | T = 17  C = 17 | Age = 44.465  %Female = 1  %White = NA | Waitlist or attention control | Breast Cancer | Ongoing curative treatment | Problem solving therapy (PST)  ***Intervention Characteristics:*** The intervention included 8 weekly sessions designed based on the Nezu’s problem-solving model by the author and consultant professor in order to improve the psycho- logical well-being of patients with breast cancer. The PST protocol consists of the following steps: Assessing the problems, setting an achievable goal related to problems, generating solution, choosing the solution, implementing the solution, and evaluating the outcome. | Psychological well-being (total); Autonomy; Environmental mastery; Personal growth; Positive relation with others; Purpose of life; Self-acceptance | PWBI |
| Trask et al, 2003 | T = 25  C = 23 | Age = 53.4  %Female =0.69  %White = 1 | Treatment as usual or standard care | Melanoma | NR | Cognitive Behavioral Therapy (CBT)  ***Intervention Characteristics:*** Individuals who were assigned randomly to the CBI treatment group received a psychiatric intake and 3 subsequent 50-minute weekly sessions focused on relaxation training, cognitive challenging, and problem solving. Each session was devoted to a discussion of one of the topics, with the content tailored to specific patient examples. Patients attended sessions once per week over a period of 4 weeks. | Physical functioning; Bodily pain; General health; Vitality; Social functioning; Mental health; STAIT | GSI; SF-36; STAI |
| Tyc et al, 1997 | T = 28  C = 27 | Age = 12.5  %Female =0.49  %White = 0.91 | Treatment as usual or standard care | Multiple cancer diagnoses | Ongoing curative treatment | Cognitive Behavioral Therapy (CBT)  ***Intervention Characteristics:*** The CBT intervention consisted of a package of five components: filmed modeling, breathing exercises, emotive imagery, behavioral rehearsal, and positive incentive. | Distress score | STAI; STAI-C |
| Wells-Di et al, 2019 | T = 17  C = 11 | Age = 56.54  %Female = 0.82  %White = 0.93 | Waitlist or attention control | Multiple cancer diagnoses | Mixed | Three‐session acceptance‐based cognitive behavioral ‐acceptance and commitment therapy (CBT‐ACT)  ***Intervention Characteristics:*** The finding this research center under stress (FOCUS) intervention included three 1.5‐hour modules (two in‐person, one via DVD) delivered over 6 weeks. CBT components of FOCUS included relaxation, sleep hygiene, sleep restriction, constructive worry, problem solving, SMART goal setting, activity pacing, and behavioral activation. ACT processes included perspective taking and recognizing worry thoughts as thoughts (ie, self‐as‐context and defusion), identifying values and acceptance of present‐moment experience in the service of values, and committed action. | Daytime sleepiness; Anxiety; Fatigue interference; Hyperarousal; Emotional distress; Physical symptoms; Cancer‐related distress | WASO; PSWQ; IUS; ESS; STAI; CES-D; FSI; JSCS; IES-R |
| Wojtyna et al, 2007 | T = 35  C = 32 | Age = 52.96  %Female = 1  %White = NA | Waitlist or attention control | Breast  cancer | Ongoing curative treatment | Cognitive Behavioral Therapy (CBT)  ***Intervention Characteristics:*** The therapy given to the subjects in this study was of a group type and included eight weekly two-hour therapeutic sessions, held at the Public Hospital in Ruda Śląska. The 10–12-patient groups consisted of patients and their support partners (spouses, friends or children). The session focused on: elements of Rational Behaviour Therapy [20] aimed at changing dysfunctional beliefs, work on imagination (visualization), training of coping with the disease and stress, improvement of the support system and interpersonal communication, work on fear of death and sense of life. | Self-esteem; Evaluation of general functioning | EORTC QLQ-C30 |
| Yanez et al, 2015 | T = 36  C = 36 | Age = 68.84  %Female = 0  %White = 0.57 | Waitlist or attention control | Multiple cancer diagnoses | Ongoing curative treatment | Cognitive-behavioral stress management (CBSM)  ***Intervention Characteristics:*** The current CBSM intervention was adapted to provide didactics and examples relevant to men with APC (e.g. impotence, incontinence, and intimacy concerns) and provide additional skills that are more appropriate for men with APC (e. g, acceptance, existential concerns, and life narratives). The manualized CBSM treatment was delivered by a group facilitator. CBSM that were discussed during the weekly group sessions as well as audio recordings of relaxation strategies (e. g, guided imagery) that study participants were encouraged to review and practice on a weekly basis. | PROMIS: depression; IES: total score; IES: intrusive thoughts; IES: hyperarousal; IES: avoidance; FACT: total; FACT: emotional; FACT: functional; FACT: physical; FACT: social; MOCS: relaxation; MOCS: assertiveness; MOCS: bonding; MOCS: awareness of tension | IES-R; FACT-G |
| Yang et al, 2022 | T = 40  C = 40 | Age = 51  %Female = NA  %White = 0 | Treatment as usual or standard care | Laryngectomy | Ongoing curative treatment | Computer-assisted Cognitive Behavioral Therapy  (cCBT)  ***Intervention Characteristics:*** The program is a remote computer program that developed by researchers. The program is based on CBT elements, and perioperative education which comprised five sessions, and each session took about 20 minutes to complete. The preoperative preparation time is usually only 5 (±2) days for patients with laryngectomy since admission and postoperative recovery time is about 10 (±2) days. The contents were reviewed and passed by clinical nursing specialists and psychotherapists. | PHQ-9; VAS -10; AIS; FACT-H&N | PHQ-9; VAS -10; AIS; FACT-H&N |
| Zhang et al, 2023 | T = 10  C = 7 | Age = 20.24  %Female = 0.706  %White = 0.647 | Active comparator | Multiple cancer diagnoses | Mixed | tCBT –Mind Your Total Health (MYTH) ***Intervention Characteristics:*** MYTH is an 8-session tCBT specifically targeting depression among AYAs  with cancer, intentionally tailored based on its parent program EntertainMeWell (EMW). Each MYTH session includes a combination of psychoeducational content delivered via video and text (including examples, vignettes) and an “episode” of the character-driven video storyline. After MYTH participants completed each session, a trained study coach reached out to participants through telephone or secure, web-based videoconference platforms (like Zoom or Google Meet) for a brief check-in call (10–15 mins). The trained study coach connected each session’s content to individual participants’ cancer diagnosis and care management. | Depression; Amxiety | PHQ-9; GAD-7; AIM |
| **400MWT** = 400 meter walk test; **ABS** = Affects Balance Scale; **ABS** = Affects Balance Scale; **ABS-NA** = Affects Balance Scale – Negative Affect; **AFI** = Attentional Function Index; **AISI** = Athens Insomnia Severity Index; **AQL** = Assessment of Quality of Life; **AUA** = American Urological Association Symptom Index; **BDI** = Beck Depression Inventory; **BDI-II** = Beck Depression Inventory, 2^nd^ version; **BFS** = Benefit Finding Scale; **BFI** = Brief Fatigue Inventory; **BFLUTS** = Bristol Female Lower Urinary Tract Symptoms Questionnaire; **B-IPQ** = Brief Illness Perception Questionnaire; **BIS** = Body Image Scale; **BPI-SF** = Brief Pain Inventory – Short Form; **BSI** = Brief Symptom Inventory; **BSI-18** = Brief Symptom Inventory, 18 items; **BSI depression** = Brief Symptom Inventory, depression subscale; **BVS** = Body Vigilance Scale; **CAPS** = Clinician-Administered PTSD Scale; **CARES** = the Cancer Rehabilitation Evaluation System; **CBCL** = Child Behavior Checklist; **CBI** = Self-Efficacy for Coping with Cancer; **CDSES** = Chronic Disease Self-Efficacy Scale; **CES-D** = Center for Epidemiologic Studies – Depression scale; **CIPN** = Chemotherapy-Induced Peripheral Neuropathy; **CIS-8R** = Checklist Individual Strength 8R; **CIS-concentration** = Checklist Individual Strength, concentration subscale; **CIS-F** = Checklist Individual Strength, Fatigue Subscale; **CLCS** = Cancer Locus of Control Scale; **COPE** = Brief COPE Inventory; **CPFCAQ** = Cancer Patients’ and Family Caregivers’ Assumptions Questionnaire; **CRS** = Concerns about Recurrence Scale; **CSES** = Cibor’s Self-Esteem Scale; **CSI-AF** = Coopersmith Self-Esteem Inventory – Adult Form; **C-SOSI** = Calgary Symptoms of Stress Inventory; **CSW** = Cancer Worry Scale; **CSQ** = Cognitive Coping Strategies Questionnaire; **CTDX** = Cancer Treatment Distress Scale; **DAQ** = Death Anxiety Questionnaire; **DAS** = Dysfunctional Attitude Scale; **DASS** = Depression, Anxiety Stress Scale; **DBAS-16** = Dysfunctional Beliefs and Attitudes About Sleep – 16; DFAS = Dysfunctional Attitudes Scale; **DHDI** = Dutch Health and Disease Inventory; **DISF** = Derogatis Inventory of Sexual Functioning; **DSD** = Daily Sleep Diaries; **DT** = Distress Thermometer; **DWI-R-Av** = Dealing with Illness Inventory – Revised-Avoidance-Defense Scale; **EORTC QLCQ 30** = European Organization for Research and Treatment of Cancer-Quality of Life Core Questionnaire-30; **EORTC QLQ-C30** = European Organisation for the Research and Treatment of Cancer Quality of Life Questionnaire. **EORTC QOL** = European Organization for Research and Treatment of Cancer Quality of Life Questionnaire; **EORTC QOQ-C30** = European Organization for Research and Treatment for Cancer Quality of Life Questionnaire – Core 30; **EPIC** = Expanded Prostate Cancer Index Composite; **EQ5-D** = EuroQol – 5 Dimension; **ESS** = Epworth Sleepiness Scale; **FACT** = Functional Assessment of Cancer Therapy; **FACT-B** = Functional Assessment of Cancer Therapy – Breast; **FACT-Cog** = Functional Assessment of Cancer Therapy – Cognitive Function; **FACT-G** = Functional Assessment of Cancer Therapy – General; **FACIT-FS** = Functional Assessment of Chronic Illness Therapy – Fatigue Subscale; **FACT-G HRQOL** = Functional Assessment of Cancer Therapy – General, which measures four domains of HRQOL; **FACT-H&N** = Functional Assessment of Cancer Therapy – Head and Neck; **FCRI** = Fear of Cancer Reoccurrence Inventory; **FFMQ** = the Five Facet Mindfulness Questionnaire; **FFMQ-SF** = Five Facet Mindfulness Questionnaire – short form; **FLI** = Functional Living Index; **FLIC** = Functional Living Index Cancer; **FLZ** = Questions on Life Satisfaction, FLZ, German version; **FMI** = Frieburg Mindfulness Inventory; **FoP-Q** = Fear of Progression Questionnaire; **FSDS-R** = Female Sexual Distress Scale – Revised; **FSFI** = Female Sexual Function Index; **FWB** = Functional Well-Being; **GAD-7** = Generalized Anxiety Disorder, 7-item; **GHQ** = General Health Questionnaire; **Global BSI** = Global Brief Symptom Inventory; **GSI** = Global Severity Index; **HADS** = Hospital Anxiety and Depression Scale; **HADS-D/HADS depression** = Hospital Anxiety and Depression Scale, Depression Subscale; **FSI** = Fatigue Symptom Inventory; **HADS anxiety** = Hospital Anxiety and Depression Scale, Anxiety Subscale; **HAM-A** = Hamilton Rating Scale for Anxiety; **HAM-D** = Hamilton Rating Scale for Depression; **HDRS** = Hamilton Depression Rating Scale; **HFNS** = Hot Flashes and Night Sweats; **HFNS frequency** = Hot Flashes and Night Sweats, frequency; **HRSD** = Hamilton Rating Scale for Depression; **HWS** = Holistic Well-being Scale; **IDSC** = Inventory of Depressive Symptoms; **IES** = Impact of Event Scale; **IES-R** = Impact of Event Scale – Revised; **IIQ** = Incontinence Impact Questionnaire; **ISI** = Insomnia Severity Inventory; **IUS** = Intolerance of Uncertainty; **JSCS** = James Supportive Care Screening; **K-10** = Kessler Psychological Distress Scale; **LASA** = Linear Analogue Self-Assessment; **LOT** = Life Orientation Test; **MAC** = Mental Adjustment to Cancer scale; **MACS** = Mental Adjustment to Cancer Scale; **MASQ** = Multiple Ability Self-Report Questionnaire; **MAX-PC** = Memorial Anxiety Scale for Prostate Cancer; **MCSD** = Marlowe Crowne Social Desirability Scale; **MDASI** = M.D. Anderson Symptom Inventory; **MDFSI** = Multidimensional Fatigue Symptom Inventory; **MHLC** = Multidimensional Health Locus of Control; **MFCV** = Muscle Fiber Conduction Velocity; **MFI** = Multidimensional Fatigue Inventory; **MFSI** = Multidimensional Fatigue Symptom Inventory – Short Form; **MHC-SF** = Mental Health Continuum – short form; **MHI** = Mental Health Inventory; **MIA-Anxiety** = Metamemory in Adulthood, anxiety subscale; **MOCS** = Measure of Current Status; **MOS** = Medical Outcomes Survey Sleep Problem Index; **MPAI-4** = Mayo-Portland Adaptability Inventory – 4; **MPQ**= McGill Pain Questionnaire; **MSQ** = the Mini Sleep Questionnaire; **NMR** = Negative Mood Regulation; **NRS** = 10-point Numeric Rating Scale; **NRS-11** = 11-point Numeric Rating Scale; **OBD** = Observed Behavioral Distress; **PAFF** = Positive Affect; **PAIS-SR** = Psychological Adjustment to Illness Scale – Self-Report; **PAM** = Patient Activation Measure; **PASE** = Physical Activity Scale for the Elderly; **PCL-C** = PTSD Checklist Civilian Version; **PCLS** = Posttraumatic Checklist – Stress-specific version; **PCS** = Pain Catastrophizing Scale; **PFS** = Patient Fatigue Scale; **PGI** = Posttraumatic Growth Inventory; **PHQ-8** = Patient Health Questionnaire, 8-item; **PHQ-9** = Patient Health Questionnaire, 9-item; **PKT** = patient Knowledge Test; **PMS** = Profile of Mood States; **PNAS** = Positive and Negative Affect Scale; **POMS** = Profile of Mood States; **POMS-SF** = Profile of Mood States – Short Form; **POMS TMS** = Profile of Mood States total mood score; **POMS anxiety** = Profile of Mood States Anxiety Subscale; **POMS depression** = Profile of Mood States Depression Subscale; **PPI** = The Present Pain Intensity; **PROMIS** = Patient-reported Outcomes Measurement Information System; **PROMIS depression** = Patient Reported Outcomes Measurement Information System; **PTCI** = Posttraumatic Cognitions Inventory; **PTGI** = Posttraumatic Growth Inventory; **PROMIS Pain Interference** = Patient-Reported Outcomes Measure Pain Interference; **PS** = Pain Scale; **PSAA** = Prostate-Specific Antigen Anxiety subscale of the Memorial Anxiety Scale for Prostate Cancer; **PSDS-SR** = Posttraumatic Stress Diagnostic Scale – Self-Report; **PSOM** = Positive State of Mind scale; **PSQI** = Pittsburgh Sleep Quality Index; **PSS** = Perceived Stress Scale; **PSS-SR** = Posttraumatic Stress Scale – Self Report; **PSWQ** = Penn State Worry Questionnaire; **PWI** = Psychological Well-being Inventory; **QL Index** = Quality of Life Index; QLQ-**BR23** = Cancer Quality of Life Breast Cancer questionnaire; **QLQ-C33-global** = The European Organization for Research and Treatment of Cancer Quality of Life Questionnaire; **QMI** = Quality of Marriage Index; **QOL-CS** = Quality of Life – Cancer Survivors; **QOLI-CV** = Quality of Life Index – Cancer Version; **QPF-R** = Psychophysiological Questionnaire – Brief Version; **QSC-23** = Questionnaire on Stress in Cancer Patients; **RPFS** = Revised Piper Fatigue Scale; **RRQ rumination** = Rumination and Reflection Questionnaire; **RSCL** = Rotterdam Symptom Checklist; **RSES** = Rosenberg Self-Esteem Scale; **RSI** = Rotterdam Symptom Checklist; **SAQ** = Symptom Assessment Questionnaires; **SAQ2** = Sexual Activity Questionnaires; **SAS** = Self-Rating Anxiety Scale; **SBQ** = Sleep Behavior Questionnaire; **SC** = Sexuality Scale; **SCL** = Symptom Check List; **SCL-90** = Symptom Check List 90; **SCL-90-R Depression** = Symptom Check List 90, revised, depression scale; **SCS-SF** = Self-Compassion Scale, Short Form; **SDS** = Symptom Distress Scale; **SES** = Symptom Experience Scale; **SES*** = Self-Esteem Scale; **SF-12 mental** = Short Form-12 mental health scale; **SF-12 physical** = Short Form-12 physical health scale; **SF-36** = General Health Survey, Short Form; **SF-MPQ-2** = Short Form McGill Pain Questionnaire 2; **SIP-8** = Sickness Impact Profile – 8; **SIP-alertness** = Sickness Impact Profile, Alertness Behavior subscale; **SSI** = Symptom Severity Index, 10 items; **SSS** = Symptom Severity Scale; **STAI** = State-Trait Anxiety Inventory; **SWB** = Social Well-Being; **SWLS** = Satisfaction with Life Scale; **TMD** = Total Mood Disturbance; **TST** = Total Sleep Time; **TWT** = Total Wake Time; **UCLA-PCI** = University of California – Loss Angeles Prostate Cancer Index Composite; **VABS** = Vineland Adaptive Behavioral Scale; **VAS global fatigue** = Visual Analogue Scale global fatigue scale; **WASO** = Wake-Time-After-Sleep-Onset; **WHO-5** = World Health Organization – 5 Well-Being Index; **WHQ** = Women’s Health Questionnaire; **YSR** = Youth Self-Report; **K-SADS-PL**: Kiddie Schedule for Affective Disorders and Schizophrenia-Present and Life-Time version; **IES** = The Impact of Event Scales; **HADS** = Hospital Anxiety and Depression Scale; **HRQOL** = Health-related Quality of Life; **mini-MAC** = mini-Mental Adjustment to Cancer Scale; **BCSSS** = Bottomley Cancer Social Support Scale; **SCNSSF** = Supportive Care Needs Survey Short Form 34; **SPS** = Social Provisions Scale; **SSTAI** = Spielberger State Trait Anxiety Inventory; **CCQ** = Cancer Coping Questionnaire; **SDPS** = Self Defined Problem Score**; PPGQ** = Participants Problem and Goals Questionnaire; **SUQ** = Services Use Questionnaire; **CREY** = Cancer Rehabilitation Evaluation System; **EEQ** = Emotional Expressiveness Questionnaire; **SSQ** = Social Support Questionnaire; **CECS** = Courtauld Emotional Control Scale; **CESD** = Center for Epidemiologic Studies-Depression; **CRTI** = Cancer-Related Thought Intrusions; **PMSSV** = Profile of Mood States-Short Version; **PGI** = Posttraumatic Growth Inventory; **PQ** = Partnership Questionnaire; **DCI** = The Dyadic Coping Inventory; **MILP** = Monash Interview for Liaison Psychiatry; **PSWQ** = Penn State Worry Questionnaire; **STAI** = The State‐Trait Anxiety Inventory; **FSI** = Fatigue Symptom Inventory; **JSCS** = James Supportive Care Screening; **PWBI** = Psychological Well-being Inventory. | | | | | | | | |

| **Supplemental Table 3. Risk of Bias Assessment** | | | | | | |
| --- | --- | --- | --- | --- | --- | --- |
| Risk of Bias | | | | | | |
|  | Randomization^1^ | Deviation^2^ | Missingness^3^ | Measurement^4^ | Reporting^5^ | Overall^6^ |
| Abrahams et al., 2017 | A | A | A | A | A | A |
| Agyemang et al., 2016 | A | A | A | A | A | A |
| Allen et al., 2002 | A | A | B | A | A | A |
| Ames et al., 2011 | A | A | A | A | A | A |
| Armes et al., 2007 | A | A | A | A | A | A |
| Arving et al., 2007 | A | A | A | A | A | A |
| Aubin et al., 2019 | A | A | A | A | A | A |
| Baucom et al., 2009 | A | A | B | A | A | A |
| Butow et al., 2017 | A | A | A | A | A | A |
| Capezzani et al., 2013 | A | A | B | A | A | A |
| Carpenter et al., 2014 | A | A | B | A | A | A |
| Casault et al., 2015 | A | A | B | A | A | A |
| Chan et al., 2017 | A | A | B | A | A | A |
| Cohen et al., 2007 | A | A | A | A | A | A |
| Compen et al., 2018 | A | A | A | A | A | A |
| Dolbeault et al., 2009 | A | A | A | A | A | A |
| Edelman et al., 1999 | A | A | B | A | A | A |
| Edelman et al., 1999 | A | A | B | A | A | A |
| Edmonds et al., 1999 | A | A | B | A | A | A |
| Ferguson et al., 2016 | A | A | B | A | A | A |
| Garland et al., 2014 | A | A | A | A | A | A |
| Germino et al., 2012 | A | A | A | A | A | A |
| Gielissen et al., 2006 | A | A | A | A | A | A |
| Goode et al., 2011 | A | A | B | A | A | A |
| Gregoire et al., 2017 | A | A | B | A | A | A |
| Gudenkauf et al., 2015 | A | A | B | A | A | A |
| Hummel et al., 2017 | A | A | A | A | A | A |
| Irwin et al., 2017 | A | A | A | A | A | A |
| Johannsen et al., 2016 | A | A | A | A | B | A |
| Johannsen et al., 2018 | A | A | A | A | A | A |
| Johansson et al., 2008 | A | A | A | A | A | A |
| Kangas et al., 2013 | A | A | A | A | A | A |
| Kazak et al., 2004 | A | A | A | A | A | A |
| Lau et al., 2020 | A | A | A | A | A | A |
| Lee et al., 2011 | A | A | B | A | A | A |
| Locke et al., 2008 | A | A | B | A | A | A |
| Mann et al., 2012 | A | A | A | A | A | A |
| Matthews et al., 2014 | A | A | A | A | A | A |
| May et al., 2009 | A | A | A | A | A | A |
| Mendoza et al., 2017 | A | A | B | A | A | A |
| Mishel et al., 2002 | A | A | B | A | A | A |
| Moon et al., 2020 | A | A | A | A | A | A |
| Mosher et al., 2016 | A | A | A | A | A | A |
| Nezu et al., 2003 | A | A | B | A | A | A |
| Nissen et al., 2020 | A | A | A | A | A | A |
| Norouzi et al., 2017 | A | A | B | A | A | A |
| Park et al., 2020 | A | A | A | A | A | A |
| Penedo et al., 2020 | A | A | A | A | A | A |
| Qiu et al., 2013 | A | A | A | A | A | A |
| Ren et al., 2019 | A | A | A | A | A | A |
| Ritterband et al., 2012 | A | A | A | A | A | A |
| Rogers et al., 2017 | A | A | A | A | A | A |
| Sandgren et al., 2000 | A | A | B | A | B | A |
| Savard et al., 2005 | A | A | A | A | A | A |
| Savard et al., 2006 | A | A | B | A | A | A |
| Stefanopoulou et al., 2015 | A | A | A | A | A | A |
| Strong et al., 2008 | A | A | A | A | A | A |
| Syrjala et al., 2018 | A | A | B | A | B | A |
| van de Wal et al., 2017 | A | A | A | A | A | A |
| van den Berg et al., 2015 | A | A | A | A | A | A |
| van der Lee et al., 2012 | A | A | B | A | A | A |
| van der Meulen et al., 2013 | A | A | A | A | A | A |
| van Weert et al., 2010 | A | A | B | A | A | A |
| Wells-Di Gregorio et al., 2019 | A | A | A | A | A | A |
| Abedini er al, 2021 | A | A | A | A | A | A |
| Acevedo-Ibarra er al, 2019 | A | A | A | A | A | A |
| Antoni et al, 2006 | B | A | A | A | A | A |
| Antoni et al, 2001 | A | A | A | A | A | A |
| Beatty et al, 2010 | A | A | A | A | A | A |
| Beatty et al, 2016 | A | A | A | A | A | A |
| Betiar et al, 2021 | A | A | A | A | A | A |
| Bottomley et al, 1996 | B | A | B | A | A | A |
| Chambers et al, 2017 | A | A | A | A | A | A |
| Chambers et al, 2014 | A | A | A | A | A | A |
| Chambers et al, 2018 | A | A | B | A | A | A |
| Cousson-Gealie et al, 2011 | A | A | A | A | A | A |
| David et al, 2013 | A | A | A | A | A | A |
| Davis et al, 1986 | B | A | A | B | A | A |
| Desautels et al, 2018 | A | A | A | A | A | A |
| Diaz et al, 2021 | A | A | A | A | A | A |
| do Camo et al, 2017 | A | B | A | A | A | A |
| Downe-Wamboldt et al, 2007 | A | A | A | A | A | A |
| DuHamel et al, 2010 | A | A | A | A | A | A |
| Evans et al, 1995 | A | B | A | A | A | A |
| Fadeal et al, 2011 | B | A | A | A | B | A |
| Ferguson et al, 2012 | A | A | A | A | A | A |
| Foley et al, 2010 | B | B | A | A | A | A |
| Garland et al, 2015 | A | A | A | A | A | A |
| Gaston-Johansson et al, 2013 | A | B | A | A | A | A |
| Graboyes et al, 2022 | A | A | A | A | A | A |
| Greer et al, 2012 | A | B | A | A | A | A |
| Groarke et al, 2013 | A | A | A | A | A | A |
| Ham et al, 2019 | A | A | A | A | A | A |
| Heinrichs et al,2012 | A | A | A | A | A | A |
| Herschbach et al, 2010 | A | A | A | A | A | A |
| Hyland et al, 2022 | A | A | A | B | A | A |
| Kingston et al, 2015 | A | A | A | A | A | A |
| Kissane et al, 2003 | A | A | A | A | A | A |
| Klosky et al, 2004 | A | A | A | B | A | A |
| Korstjens e al,2011 | A | A | A | A | A | A |
| Lechner et al, 2014 | A | A | A | A | A | A |
| Mann et al, 2012 | B | A | A | A | A | A |
| Manne et al, 2007 | A | A | A | A | A | A |
| Manne et al, 2017 | A | A | A | A | A | A |
| Marchioro et al,1996 | A | A | A | A | A | A |
| Merckaert et al, 2017 | A | A | A | A | A | A |
| Mishel et al, 2005 | A | A | A | A | A | A |
| Mokrivala et al, 2022 | A | A | A | A | A | A |
| Moorey et al, 1998 | B | B | A | A | A | A |
| Murphy et al, 2020 | A | B | A | A | A | A |
| Onyechi et al, 2016 | A | A | A | A | A | A |
| Peoles et al, 2017 | B | A | B | A | A | A |
| Phillips et al, 2011 | A | A | A | A | A | A |
| Richardson et al, 2017 | A | A | A | A | A | A |
| Serfaty et al, 2000 | A | A | A | A | A | A |
| Serfaty et al, 2012 | A | B | A | A | A | A |
| Solkoglu et al, 2023 | A | A | A | A | A | A |
| Speer, 1987 | B | A | A | A | A | A |
| Stagel et al, 2015 | A | B | A | A | A | A |
| Taeidi et al, 2018 | A | A | A | A | A | A |
| Trask et al, 2003 | A | B | A | A | A | A |
| Tyc et al, 1997 | B | A | A | A | A | A |
| Wells-Di et al, 2019 | A | A | A | A | A | A |
| Yanez et al, 2015 | A | A | A | A | A | A |
| Yang et al, 2022 | A | A | A | A | A | A |
| Zhang et al, 2023 | A | A | A | A | A | A |
| * A = Low risk of bias (green color); C = High risk of bias (red color); B = Some concerns (Mid-level risk of bias, yellow color)  1. Risk of bias arising from the randomization process  2. Risk of bias due to deviations from the intended interventions (effect of assignment to intervention)  3. Missing outcome data  4. Risk of bias in measurement of the outcome  5. Risk of bias in selection of the reported results  6. Overall risk of bias | | | | | | |

| **Supplemental Table 2. Risk of Bias Assessment** | | | | | | | | |
| --- | --- | --- | --- | --- | --- | --- | --- | --- |
| ROBINS-I | | | | | | | | |
|  | Confounding^1^ | Participant^2^ | Classification^3^ | Deviation^4^ | Missingness^5^ | Measurement^6^ | Reporting^7^ | Overall^8^ |
| Bragard et al., 2017 | A | A | A | A | A | A | B | A |
| Cole et al., 1999 | B | B | A | A | A | A | B | A |
| Bottomley et al, 1996 | A | A | A | A | A | A | A | A |
| Chen et al, 2014 | A | B | A | A | A | A | A | A |
| McKierman et al, 2010 | A | A | A | A | A | A | A | A |
| Poggi et al, 2009 | A | A | A | A | A | A | A | A |
| Wojtyna et al, 2007 | A | A | A | A | B | A | A | A |
| Zhang et al., 2019 | A | A | A | A | B | A | A | A |
| * A = Low risk of bias (green color); C = High risk of bias (red color); B = Some concerns (Mid-level risk of bias, yellow color)  1. Bias due to confounding  2. Bias in selection of participants into the study  3. Bias in classification of interventions  4. Bias due to deviations from intended interventions  5. Bias due to missing data  6. Bias in measurement of outcomes  7. Bias in selection of the reported result  8. Overall bias | | | | | | | | |

**Supplement 1. Search Strategy and Databases/Sources**

**Search Strategy:**

***Search term to identify cognitive behavioral therapy:***

[general term] “Cognitive therapy” or “cognitive behavior therapy” or “cognitive-behavior therapy” or “cognitive behavioral therapy” or “cognitive-behavioral therapy” or “behavior therapy” or “behavioral therapy” or “cognitive behaviour therapy” or “cognitive-behaviour therapy” or “cognitive behavioural therapy” or “cognitive-behavioural therapy” or “behaviour therapy” or “behavioural therapy” or “behavioral activation” or “behavioural activation” or “problem solving” or “problem-solving”

Search term to identify cancer:

[general term] “Cancer” or “oncolog*” or “hematolog*” or “malignan*” or “tumor” or “neoplasm*” or “carcinoma*”

**Databases:**

***********************************

CINAHL Complete

Global Health

APA PsycArticles

APA PsycInfo

Social Science Abstracts

***********************************

Cochrane Database

***********************************

Medline [MeSH Term]

**Cognitive Behavioral Therapy**

- Behavioral Therapies, Cognitive
- Behavioral Therapy, Cognitive
- Cognitive Behavioral Therapies
- Therapies, Cognitive Behavioral
- Therapy, Cognitive Behavioral
- Therapy, Cognitive Behavior
- Cognitive Behavior Therapy
- Cognitive Therapy
- Behavior Therapy, Cognitive
- Behavior Therapies, Cognitive
- Cognitive Behavior Therapies
- Therapies, Cognitive Behavior
- Cognitive Psychotherapy
- Cognitive Psychotherapies
- Psychotherapies, Cognitive
- Psychotherapy, Cognitive
- Therapy, Cognitive
- Cognitive Therapies
- Therapies, Cognitive
- Cognition Therapy
- Therapy, Cognition
- Cognition Therapies
- Therapies, Cognition

**Cancer**

- Neoplasm
- Neoplasms
- Tumor
- Tumors
- Cancer
- Cancers
- Oncology
- Carcinoma
- Carcinomas
- Malignant
- Malignancy

**Full list of Reference of Existing Review Articles:**

1. [002] Mehta, S., Peynenburg, V. A., & Hadjistavropoulos, H. D. (2019). Internet-delivered cognitive behaviour therapy for chronic health conditions: A systematic review and meta-analysis. Journal of Behavioral Medicine, 42(2), 169-187. [ICBT for D&A among chronic conditions, 1 cancer trial]
2. [005] Getu, M. A., Chen, C., Panpan, W., Mboineki, J. F., Dhakal, K., & Du, R. (2020). The effect of cognitive behavioral therapy on the quality of life of breast cancer patients: a systematic review and meta-analysis of randomized controlled trials. Quality of Life Research, 1-18. [CBT for Quality of Life among Breast Cancer Patients, 11 RCTs included]
3. [006] Wu, C., Zheng, Y., Duan, Y., Lai, X., Cui, S., Xu, N., ... & Lu, L. (2019). Nonpharmacological interventions for cancer‐related fatigue: a systematic review and Bayesian network meta‐analysis. Worldviews on Evidence‐Based Nursing, 16(2), 102-110. [182 RCTs included, but no clear sign of how many are CBT trials.]
4. [007] Ye, M., Du, K., Zhou, J., Zhou, Q., Shou, M., Hu, B., ... & Liu, Z. (2018). A meta‐analysis of the efficacy of cognitive behavior therapy on quality of life and psychological health of breast cancer survivors and patients. Psycho‐oncology, 27(7), 1695-1703. [10 RCTs were included for CBT on QoL and Psychological Health among breast cancer survivors and patients]
5. [009] Sun, H., Huang, H., Ji, S., Chen, X., Xu, Y., Zhu, F., & Wu, J. (2019). The efficacy of cognitive behavioral therapy to treat depression and anxiety and improve quality of life among early-stage breast cancer patients. Integrative cancer therapies, 18, 1534735419829573. [8 RCTs of CBT for D&A and QoL in early stage Breast Cancer Patients]
6. [010] Chen, D., Sun, W., Liu, N., Wang, J., Zhao, J., Zhang, Y., ... & Zhang, W. (2018, November). Fear of Cancer Recurrence: A Systematic Review of Randomized, Controlled Trials. In Oncology nursing forum (Vol. 45, No. 6). [Included 10 studies, with 1 reporting CBT for FCR]
7. [013] Johnson, J. A., Rash, J. A., Campbell, T. S., Savard, J., Gehrman, P. R., Perlis, M., ... & Garland, S. N. (2016). A systematic review and meta-analysis of randomized controlled trials of cognitive behavior therapy for insomnia (CBT-I) in cancer survivors. Sleep medicine reviews, 27, 20-28. [8 Studies CBT for Insomnia in Cancer Survivors]
8. [015] Tang, M., Liu, X., Wu, Q., & Shi, Y. (2020). The effects of cognitive-behavioral stress management for breast cancer patients: a systematic review and meta-analysis of randomized controlled trials. Cancer nursing, 43(3), 222-237. [18 RCTs of Cognitive Behavioral Stress Management for patients’ coping with stress and negative effects of cancer.]
9. [018] Zeichner, S. B., Zeichner, R. L., Gogineni, K., Shatil, S., & Ioachimescu, O. (2017). Cognitive behavioral therapy for insomnia, mindfulness, and yoga in patients with breast cancer with sleep disturbance: A literature review. Breast cancer: Basic and Clinical Research, 11, 1178223417745564. [Not perfect fit, but reference list is worth screening]
10. [019] Teo, I., Krishnan, A., & Lee, G. L. (2019). Psychosocial interventions for advanced cancer patients: A systematic review. Psycho‐oncology, 28(7), 1394-1407. [Classified 20 studies that are CBT-based]
11. [020] Matthews, H., Grunfeld, E. A., & Turner, A. (2017). The efficacy of interventions to improve psychosocial outcomes following surgical treatment for breast cancer: A systematic review and meta‐analysis. Psycho‐oncology, 26(5), 593-607. [Included 8 CBT studies should be screened for]
12. [023] Jibb, L. A., Nathan, P. C., Stevens, B. J., Yohannes, L., & Stinson, J. N. (2015, November). Psychological and physical interventions for the management of cancer-related pain in pediatric and young adult patients: an integrative review. In Oncology Nursing Forum (Vol. 42, No. 6, p. E339). Oncology Nursing Society. [Larger review, but included 9 CBT studies]
13. [024] Cobeanu, O., & David, D. (2018). Alleviation of side effects and distress in breast cancer patients by cognitive-behavioral interventions: A systematic review and meta-analysis. Journal of clinical psychology in medical settings, 25(4), 335-355. [19 to 21 clinical trials of CBT for Breast Cancer Patients]
14. [025] Tao, W. W., Tao, X. M., & Song, C. L. (2017). Effects of non-pharmacological supportive care for hot flushes in breast cancer: a meta-analysis. Supportive Care in Cancer, 25(7), 2335-2347. [Included 24 studies, but only 2 or 1 CBT studies]
15. [026] Dimitrov, L., Moschopoulou, E., & Korszun, A. (2019). Interventions for the treatment of cancer‐related traumatic stress symptoms: A systematic review of the literature. Psycho‐oncology, 28(5), 970-979. [Included 8 studies, many are CBT or CBT informed treatment]
16. [027] Xiao, F., Song, X., Chen, Q., Dai, Y., Xu, R., Qiu, C., & Guo, Q. (2017). Effectiveness of psychological interventions on depression in patients after breast cancer surgery: a meta-analysis of randomized controlled trials. Clinical breast cancer, 17(3), 171-179. [Included a total of 13 articles, and 8 or 9 of the studies were CBT related]
17. [028] Tatrow, K., & Montgomery, G. H. (2006). Cognitive behavioral therapy techniques for distress and pain in breast cancer patients: a meta-analysis. Journal of Behavioral Medicine, 29(1), 17-27. [Highly relevant, a total of 20 studies]
18. [029] Oh, P. J. (2016, September). The effects of nonpharmacologic interventions on cognitive function in patients with cancer: A meta-analysis. Oncology Nursing Forum, 43(5). [1 CBT study]
19. [030] Oh, P. J., Shin, S. R., Ahn, H. S., & Kim, H. J. (2016). Meta-analysis of psychosocial interventions on survival time in patients with cancer. Psychology & health, 31(4), 396-419. [3 CBT studies]
20. [031] Lopresti, A. L. (2017). Cognitive behaviour therapy and inflammation: a systematic review of its relationship and the potential implications for the treatment of depression. Australian & New Zealand Journal of Psychiatry, 51(6), 565-582. [Not directly related to cancer, but included at least 2 breast cancer related trials]
21. [032] Sheikh-Wu, S. F., Downs, C. A., & Anglade, D. (2020). Interventions for managing a symptom cluster of pain, fatigue, and sleep disturbances during cancer survivorship: A systematic review. Oncology Nursing Forum, 47(4), 107-119. [3 potential trials of CBT mentioned in this review]
22. [033] Ma, Y., Hall, D. L., Ngo, L. H., Liu, Q., Bain, P. A., & Yeh, G. Y. (2020). Efficacy of cognitive behavioral therapy for insomnia in breast cancer: a meta-analysis. Sleep Medicine Reviews, 101376. [14 RCTs of CBT for insomnia in breast cancer]
23. [035] Abrahams, H. J., Knoop, H., Schreurs, M., Aaronson, N. K., Jacobsen, P. B., Newton, R. U., ... & Buffart, L. M. (2020). Moderators of the effect of psychosocial interventions on fatigue in women with breast cancer and men with prostate cancer: Individual patient data meta‐analyses. Psycho‐Oncology, 29(11), 1772-1785. [7 CBT for fatigues in women with breast and men with prostate cancer]
24. [036] Brebach, R., Sharpe, L., Costa, D. S., Rhodes, P., & Butow, P. (2016). Psychological intervention targeting distress for cancer patients: a meta‐analytic study investigating uptake and adherence. Psycho‐Oncology, 25(8), 882-890. [2 CBT related trials for cancer in the reference list]
25. [037] Moraes, L. J., Miranda, M. B., Loures, L. F., Mainieri, A. G., & Mármora, C. H. C. (2018). A systematic review of psychoneuroimmunology-based interventions. Psychology, health & medicine, 23(6), 635-652. [2 CBT studies for cancer in the reference list]
26. [038] D’egidio, V., Sestili, C., Mancino, M., Sciarra, I., Cocchiara, R., Backhaus, I., ... & La Torre, G. (2017). Counseling interventions delivered in women with breast cancer to improve health-related quality of life: A systematic review. Quality of Life Research, 26(10), 2573-2592. [a couple of CBT studies, not sure how many exactly]
27. [040] Ehrenzeller, M. F., & Adam Goldstein, M. D. (2018). Smoking prevalence and management among cancer survivors. In Oncology nursing forum (Vol. 45, No. 1, pp. 55-68). Oncology Nursing Society. [a couple of relevant CBT studies for cancer smoke cessation, but not sure how many exactly]
28. [042] Hofmann, S. G., Sawyer, A. T., Witt, A. A., & Oh, D. (2010) The effect of mindfulness-based therapy on anxiety and depression: A meta-analytic review. Journal of Consulting and Clinical Psychology, 78(2), 169-183. [possible mindfulness CBT for cancer patients]
29. [043] Garland, S. N., Johnson, J. A., Savard, J., Gehrman, P., Perlis, M., Carlson, L., & Campbell, T. (2014). Sleeping well with cancer: a systematic review of cognitive behavioral therapy for insomnia in cancer patients. Neuropsychiatric Disease and Treatment, 10, 1113-1124. [CBT for insomnia for cancer patients, 13 included studies]
30. [044] Cillessen, L., Johannsen, M., Speckens, A. E., & Zachariae, R. (2019). Mindfulness‐based interventions for psychological and physical health outcomes in cancer patients and survivors: A systematic review and meta‐analysis of randomized controlled trials. Psycho‐Oncology, 28(12), 2257-2269. [mindfulness cognitive therapy for cancer patients]
31. [045] Petricone-Westwood, D., Jones, G., Mutsaers, B., Leclair, C. S., Tomei, C., Trudel, G., ... & Lebel, S. (2019). A systematic review of interventions for health anxiety presentations across diverse chronic illnesses. International Journal of Behavioral Medicine, 26(1), 3-16. [at least 3 RCTs of CBT for anxiety in patients with chronic condition]
32. [046] Haller, H., Winkler, M. M., Klose, P., Dobos, G., Kuemmel, S., & Cramer, H. (2017). Mindfulness-based interventions for women with breast cancer: an updated systematic review and meta-analysis. Acta Oncologica, 56(12), 1665-1676. [A couple of studies on mindfulness-based cognitive therapy for breast cancer patients]
33. [048] Margaretta Page, M. S. (2018). Sleep-wake disturbance: a systematic review of evidence-based interventions for management in patients with cancer. Clinical Journal of Oncology Nursing, 22(1), 37-52. [CBT for patients with cancer]
34. [049] Mu, P. F., Chen, Y. C., & Cheng, S. C. (2009). The effectiveness of non-pharmacological pain management in relieving chronic pain for children and adolescents. JBI Evidence Synthesis, 7(34), 1489-1543. [possible CBT study for young cancer survivor] [no eligible studies identified]
35. [050] MENP, K. J. M. (2016). Effective transitional therapy for adolescent and young adult patients with cancer: an integrative literature review. Clinical Journal of Oncology Nursing, 20(4), 391. [possible one or more CBT study for cancer]
36. [051] Fors, E. A., Bertheussen, G. F., Thune, I., Juvet, L. K., Elvsaas, I. K. Ø., Oldervoll, L., ... & Leivseth, G. (2011). Psychosocial interventions as part of breast cancer rehabilitation programs? Results from a systematic review. Psycho‐Oncology, 20(9), 909-918. [including CBT study for breast cancer rehabilitation]
37. [053] Chien, C. H., Liu, K. L., Chien, H. T., & Liu, H. E. (2014). The effects of psychosocial strategies on anxiety and depression of patients diagnosed with prostate cancer: A systematic review. International Journal of Nursing Studies, 51(1), 28-38. [include, psychosocial include CBT]
38. [054] Luckett, T., Britton, B., Clover, K., & Rankin, N. M. (2011). Evidence for interventions to improve psychological outcomes in people with head and neck cancer: a systematic review of the literature. Supportive Care in Cancer, 19(7), 871-881. [at least one study of cognitive therapy, likely more]
39. [055] Möller, U. O., Beck, I., Rydén, L., & Malmström, M. (2019). A comprehensive approach to rehabilitation interventions following breast cancer treatment-a systematic review of systematic reviews. BMC Cancer, 19(1), 1-20. [CBT study for breast cancer] [references do not include article title!]
40. [056] Coughtrey, A., Millington, A., Bennett, S., Christie, D., Hough, R., Su, M. T., ... & Shafran, R. (2018). The effectiveness of psychosocial interventions for psychological outcomes in pediatric oncology: a systematic review. Journal of Pain and Symptom Management, 55(3), 1004-1017. [possible CBT study]
41. [057] Fulton, J. J., Newins, A. R., Porter, L. S., & Ramos, K. (2018). Psychotherapy targeting depression and anxiety for use in palliative care: a meta-analysis. Journal of Palliative Medicine, 21(7), 1024-1037. [possible CBT study for pediatric cancer patients]
42. [058] Cheng, L., & Kotronoulas, G. (2020). How effective are self-management interventions in promoting health-related qualify of life in people after primary treatment for breast cancer? A critical evidence synthesis. European Journal of Oncology Nursing, 101776. [at least 5 CBT based studies]
43. [061] Hines, S., Ramis, M. A., Pike, S., & Chang, A. M. (2011). Effectiveness of psychosocial interventions for cognitive dysfunction in cancer patients who have received chemotherapy: A systematic review. JBI Evidence Synthesis, 9(49), 2023-2062. [include CBT studies for cancer patients and survivors]
44. [062] Chang, C. W., Mu, P. F., Jou, S. T., Wong, T. T., & Chen, Y. C. (2012). The effectiveness of non-pharmacological interventions on fatigue in children and adolescents with cancer: a systematic review. JBI Evidence Synthesis, 10(10), 574-614. [there are CBT studies for fatigue among young adults with cancer]
45. [066] Hart, S. L., Hoyt, M. A., Diefenbach, M., Anderson, D. R., Kilbourn, K. M., Craft, L. L., ... & Stanton, A. L. (2012). Meta-analysis of efficacy of interventions for elevated depressive symptoms in adults diagnosed with cancer. Journal of the National Cancer Institute, 104(13), 990-1004. [include a couple of CBT studies]
46. [068] Campbell, C. L., & Campbell, L. C. (2012). A systematic review of cognitive behavioral interventions in advanced cancer. Patient Education and Counseling, 89(1), 15-24. [11 studies met the inclusion criteria]
47. [070] Stefanopoulou, E., & Grunfeld, E. A. (2017). Mind–body interventions for vasomotor symptoms in healthy menopausal women and breast cancer survivors. A systematic review. Journal of Psychosomatic Obstetrics & Gynecology, 38(3), 210-225. [about 11 studies of CBT for breast cancer patients or survivors]
48. [072] Momani, T. E. G., & Berry, D. L. (2017). Integrative therapeutic approaches for the management and control of nausea in children undergoing cancer treatment: a systematic review of literature. Journal of Pediatric Oncology Nursing, 34(3), 173-184. [a couple of included study both in text and in the reference list]
49. [073] Ernst, E., Schmidt, K., & Baum, M. (2006). Complementary/alternative therapies for the treatment of breast cancer. A systematic review of randomized clinical trials and a critique of current terminology. The Breast Journal, 12(6), 526-530. [a couple of CBT studies]
50. [075] Paul, C. L., Carey, M. L., Sanson-Fisher, R. W., Houlcroft, L. E., & Turon, H. E. (2013). The impact of web-based approaches on psychosocial health in chronic physical and mental health conditions. Health Education Research, 28(3), 450-471. [a couple of studies]
51. [076] Mitchell, S. A., Hoffman, A. J., Clark, J. C., DeGennaro, R. M., Poirier, P., Robinson, C. B., & Weisbrod, B. L. (2014). Putting evidence into practice: an update of evidence-based interventions for cancer-related fatigue during and following treatment. Clinical Journal of Oncology Nursing, 18, 38-58. [several CBT studies]
52. [077] Post, K. E., & Flanagan, J. (2016). Web based survivorship interventions for women with breast cancer: An integrative review. European Journal of Oncology Nursing, 25, 90-99. [a couple of eligible reviews]
53. [079] O’Connor, M., & Halkett, G. K. (2019). A systematic review of interventions to reduce psychological distress in pediatric patients receiving radiation therapy. Patient Education and Counseling, 102(2), 275-283. [several CBT studies]
54. [080] Nenova, M., Morris, L., Paul, L., Li, Y., Applebaum, A., & DuHamel, K. (2013). Psychosocial interventions with cognitive-behavioral components for the treatment of cancer-related traumatic stress symptoms: A review of randomized controlled trials. Journal of Cognitive Psychotherapy, 27(3), 258-284. [clearly relevant study and will have studies for inclusion]
55. [081] Beesley, V. L., Alemayehu, C., & Webb, P. M. (2019). A systematic literature review of trials of survivorship interventions for women with gynaecological cancer and their caregivers. European Journal of Cancer Care, 28(3), e13057. [should be included, relevant]
56. [082] Dale, H. L., Adair, P. M., & Humphris, G. M. (2010). Systematic review of post‐treatment psychosocial and behaviour change interventions for men with cancer. Psycho‐Oncology: Journal of the Psychological, Social and Behavioral Dimensions of Cancer, 19(3), 227-237. [about 11 included reviews]
57. [083] Larkin, D., Lopez, V., & Aromataris, E. (2014). Managing cancer‐related fatigue in men with prostate cancer: A systematic review of non‐pharmacological interventions. International Journal of Nursing Practice, 20(5), 549-560. [should be included, a couple of studies]
58. [084] Osborn, R. L., Demoncada, A. C., & Feuerstein, M. (2006). Psychosocial interventions for depression, anxiety, and quality of life in cancer survivors: meta-analyses. The International Journal of Psychiatry in Medicine, 36(1), 13-34. [Include]
59. [087] Haerizadeh, M., Sumner, J. A., Birk, J. L., Gonzalez, C., Heyman-Kantor, R., Falzon, L., ... & Kronish, I. M. (2020). Interventions for posttraumatic stress disorder symptoms induced by medical events: A systematic review. Journal of Psychosomatic Research, 129, 109908. [include 1 study of EMDR vs CBT for cancer patients]
60. [088] Zee, P. C., & Ancoli-Israel, S. (2009). Does effective management of sleep disorders reduce cancer-related fatigue?. Drugs, 69(2), 29-41. [likely 4 or more studies]
61. [089] McCarter, K., Martínez, Ú., Britton, B., Baker, A., Bonevski, B., Carter, G., ... & Wolfenden, L. (2016). Smoking cessation care among patients with head and neck cancer: a systematic review. BMJ Open, 6(9), e012296. [1 CBT study]
62. [090] Lee, Y. H., Chiou, P. Y., Chang, P. H., & Hayter, M. (2011). A systematic review of the effectiveness of problem‐solving approaches towards symptom management in cancer care. Journal of clinical nursing, 20(1‐2), 73-85. [Problem-solving therapy for cancer survivor]
63. [091] Richardson, A. E., Broadbent, E., & Morton, R. P. (2019). A systematic review of psychological interventions for patients with head and neck cancer. Supportive Care in Cancer, 27(6), 2007-2021. [at least 5 RCT studies]
64. [092] Kangas, M., Bovbjerg, D. H., & Montgomery, G. H. (2008). Cancer-related fatigue: a systematic and meta-analytic review of non-pharmacological therapies for cancer patients. Psychological Bulletin, 134(5), 700. [19 trials reporting fatigue outcome]
65. [093] Berry, E., Davies, M., & Dempster, M. (2017). Exploring the effectiveness of couples interventions for adults living with a chronic physical illness: A systematic review. Patient Education and Counseling, 100(7), 1287-1303. [Include, likely have eligible study, but also likely overlapping with other studies]
66. [094] de M Alcântara‐Silva, T. R., Freitas‐Junior, R., Freitas, N. M., & Machado, G. D. (2013). Fatigue related to radiotherapy for breast and/or gynaecological cancer: a systematic review. Journal of Clinical Nursing, 22(19-20), 2679-2686. [include CBT study for breast cancer patients]
67. [096] Corbett, T. K., Groarke, A., Devane, D., Carr, E., Walsh, J. C., & McGuire, B. E. (2019). The effectiveness of psychological interventions for fatigue in cancer survivors: systematic review of randomised controlled trials. Systematic Reviews, 8(1), 1-30. [include CBT study for cancer patients and survivors]
68. [097] Hoon, L. S., Sally, C. W. C., & Hong-Gu, H. (2013). Effect of psychosocial interventions on outcomes of patients with colorectal cancer: a review of the literature. European Journal of Oncology Nursing, 17(6), 883-891. [include CBT study]
69. [099] Haber, J. (2004). existing evidence does not support a survival benefit for women with metastatic breast cancer who participate in group psychological interventions compared with usual care. Evidence-based nursing, 7(4), 110. [This is included, but the article is not valuable.]
70. [100] Meyer, T. J., & Mark, M. M. (1999). Effects of psychosocial interventions with adult cancer patients: A meta-analysis of randomized experiments. Health Psychology, 14(2), 101-106. [include a CBT category]
71. [102] Kang, H. S., Kim, H. K., Park, S. M., & Kim, J. H. (2018). Online-based interventions for sexual health among individuals with cancer: a systematic review. BMC Health Services Research, 18(1), 1-10. [include a couple CBT studies for cancer]
72. [103] Duijts, S. F., Faber, M. M., Oldenburg, H. S., van Beurden, M., & Aaronson, N. K. (2011). Effectiveness of behavioral techniques and physical exercise on psychosocial functioning and health‐related quality of life in breast cancer patients and survivors—a meta‐analysis. Psycho‐Oncology, 20(2), 115-126. [relevant, include CBT studies for cancer]
73. [106] Dy, S. M., & Apostol, C. C. (2010). Evidence-based approaches to other symptoms in advanced cancer. The Cancer Journal, 16(5), 507-513. [include CBT studies]
74. [107] Chien, C. H., Liu, K. L., Chien, H. T., & Liu, H. E. (2014). The effects of psychosocial strategies on anxiety and depression of patients diagnosed with prostate cancer: a systematic review. International Journal of Nursing Studies, 51(1), 28-38. [include CBT studies]
75. [109] Toivonen, K. I., Zernicke, K., & Carlson, L. E. (2017). Web-based mindfulness interventions for people with physical health conditions: systematic review. Journal of Medical Internet Research, 19(8), e303. [a couple of mindfulness CBT study for cancer patient]
76. [112] Hersch, J., Juraskova, I., Price, M., & Mullan, B. (2009). Psychosocial interventions and quality of life in gynaecological cancer patients: a systematic review. Psycho‐Oncology: Journal of the Psychological, Social and Behavioral Dimensions of Cancer, 18(8), 795-810. [at least 3 or more cognitive behavioral therapy]
77. [113] Tremblay, A., Sheeran, L., & Aranda, S. K. (2008). Psychoeducational interventions to alleviate hot flashes: a systematic review. Menopause, 15(1), 193-202. [possible study to be included]
78. [115] Hart, S. L., Hoyt, M. A., Diefenbach, M., Anderson, D. R., Kilbourn, K. M., Craft, L. L., ... & Stanton, A. L. (2012). Meta-analysis of efficacy of interventions for elevated depressive symptoms in adults diagnosed with cancer. Journal of the National Cancer Institute, 104(13), 990-1004. [a couple of CBT studies]
79. [118] Akechi, T., Okuyama, T., Onishi, J., Morita, T., & Furukawa, T. A. (2008). Psychotherapy for depression among incurable cancer patients. Cochrane Database of Systematic Reviews, (2). [a Cochrane, should be included]
80. [119] Fjorback, L. O., Arendt, M., Ørnbøl, E., Fink, P., & Walach, H. (2011). Mindfulness‐Based Stress Reduction and Mindfulness‐Based Cognitive Therapy–a systematic review of randomized controlled trials. Acta Psychiatrica Scandinavica, 124(2), 102-119. [mindfulness-based cognitive therapy for cancer patients]
81. [121] Tauber, N. M., O’Toole, M. S., Dinkel, A., Galica, J., Humphris, G., Lebel, S., ... & Zachariae, R. (2019). Effect of psychological intervention on fear of cancer recurrence: A systematic review and meta-analysis. Journal of Clinical Oncology, 37(31), 2899-2915. [include, contains eligible study]
82. [122 Cochrane search 1] Mustafa, M., Carson‐Stevens, A., Gillespie, D., & Edwards, A. G. (2013). [Psychological interventions for women with metastatic breast cancer](https://www.cochranelibrary.com/cdsr/doi/10.1002/14651858.CD004253.pub4/full). Cochrane Database of Systematic Reviews, (6).
83. [123 Cochrane search 2] Thabrew, H., Stasiak, K., Hetrick, S. E., Donkin, L., Huss, J. H., Highlander, A., ... & Merry, S. N. (2018). [Psychological therapies for anxiety and depression in children and adolescents with long‐term physical conditions](https://www.cochranelibrary.com/cdsr/doi/10.1002/14651858.CD012488.pub2/full). Cochrane Database of Systematic Reviews, (12).
84. [124 Cochrane search 3] Jassim, G. A., Whitford, D. L., Hickey, A., & Carter, B. (2015). [Psychological interventions for women with non‐metastatic breast cancer](https://www.cochranelibrary.com/cdsr/doi/10.1002/14651858.CD008729.pub2/full). Cochrane Database of Systematic Reviews, (5).
85. [125 Cochrane search 4] Law, E., Fisher, E., Eccleston, C., & Palermo, T. M. (2019). [Psychological interventions for parents of children and adolescents with chronic illness.](https://www.cochranelibrary.com/cdsr/doi/10.1002/14651858.CD009660.pub4/full) Cochrane Database of Systematic Reviews, (3).
86. [126 Cochrane search 5] Thabrew, H., Stasiak, K., Hetrick, S. E., Wong, S., Huss, J. H., & Merry, S. N. (2018). E‐Health interventions for anxiety and depression in children and adolescents with long‐term physical conditions. Cochrane Database of Systematic Reviews, (8).
87. [130 Cochrane search 8] Uphoff, E., Pires, M., Barbui, C., Barua, D., Churchill, R., Cristofalo, D., ... & Siddiqi, N. (2020). Behavioural activation therapy for depression in adults with non‐communicable diseases. Cochrane Database of Systematic Reviews, (8).
88. [132 Cochrane search 11] Clarkson, J. E., Worthington, H. V., Furness, S., Mccabe, M., Khalid, T., & Meyer, S. (2010). Interventions for treatment oral mucositis for patients with cancer receiving treatment. Cochrane Database of Systematic Reviews, (8).
89. [133 Cochrane search 12] Poort, H., Peters, M., Bleijenberg, G., Gielissen, M. F., Goedendorp, M. M., Jacobsen, P., ... & Knoop, H. (2017). Psychosocial interventions for fatigue during cancer treatment with palliative intent. Cochrane Database of Systematic Reviews, (7).
90. [137 Cochrane search 16] Parahoo, K., McDonough, S., McCaughan, E., Noyes, J., Semple, C., Halstead, E. J., ... & Dahm, P. (2013). Psychosocial interventions for men with prostate cancer. Cochrane Database of Systematic Reviews, (12).
91. [140 Cochrane search 19] Cheng, K. K. F., Lim, Y. T. E., Koh, Z. M., & San Tam, W. W. (2017). Home‐based multidimensional survivorship programmes for breast cancer survivors. Cochrane Database of Systematic Reviews, (8).
