# Supplementary material for: The efficacy of cognitive behavioral therapy for mental health and quality of life among individuals diagnosed with cancer: A systematic review and meta‐analysis
Source: Cancer Med. 2024 Aug 21;13(16):e70063. doi: 10.1002/cam4.70063 (PMC11336377; doi:10.1002/cam4.70063)
Supplement: Supplementary file 3 — Data S3. [file CAM4-13-e70063-s001.docx]

**Supplement 1. Search Strategy and Databases/Sources**

**Search Strategy:**

***Search term to identify cognitive behavioral therapy:***

[general term] “Cognitive therapy” or “cognitive behavior therapy” or “cognitive-behavior therapy” or “cognitive behavioral therapy” or “cognitive-behavioral therapy” or “behavior therapy” or “behavioral therapy” or “cognitive behaviour therapy” or “cognitive-behaviour therapy” or “cognitive behavioural therapy” or “cognitive-behavioural therapy” or “behaviour therapy” or “behavioural therapy” or “behavioral activation” or “behavioural activation” or “problem solving” or “problem-solving”

Search term to identify cancer:

[general term] “Cancer” or “oncolog*” or “hematolog*” or “malignan*” or “tumor” or “neoplasm*” or “carcinoma*”

**Databases:**

***********************************

CINAHL Complete

Global Health

APA PsycArticles

APA PsycInfo

Social Science Abstracts

***********************************

Cochrane Database

***********************************

Medline [MeSH Term]

**Cognitive Behavioral Therapy**

- Behavioral Therapies, Cognitive
- Behavioral Therapy, Cognitive
- Cognitive Behavioral Therapies
- Therapies, Cognitive Behavioral
- Therapy, Cognitive Behavioral
- Therapy, Cognitive Behavior
- Cognitive Behavior Therapy
- Cognitive Therapy
- Behavior Therapy, Cognitive
- Behavior Therapies, Cognitive
- Cognitive Behavior Therapies
- Therapies, Cognitive Behavior
- Cognitive Psychotherapy
- Cognitive Psychotherapies
- Psychotherapies, Cognitive
- Psychotherapy, Cognitive
- Therapy, Cognitive
- Cognitive Therapies
- Therapies, Cognitive
- Cognition Therapy
- Therapy, Cognition
- Cognition Therapies
- Therapies, Cognition

**Cancer**

- Neoplasm
- Neoplasms
- Tumor
- Tumors
- Cancer
- Cancers
- Oncology
- Carcinoma
- Carcinomas
- Malignant
- Malignancy

**Full list of Reference of Existing Review Articles:**

1. [002] Mehta, S., Peynenburg, V. A., & Hadjistavropoulos, H. D. (2019). Internet-delivered cognitive behaviour therapy for chronic health conditions: A systematic review and meta-analysis. Journal of Behavioral Medicine, 42(2), 169-187. [ICBT for D&A among chronic conditions, 1 cancer trial]
2. [005] Getu, M. A., Chen, C., Panpan, W., Mboineki, J. F., Dhakal, K., & Du, R. (2020). The effect of cognitive behavioral therapy on the quality of life of breast cancer patients: a systematic review and meta-analysis of randomized controlled trials. Quality of Life Research, 1-18. [CBT for Quality of Life among Breast Cancer Patients, 11 RCTs included]
3. [006] Wu, C., Zheng, Y., Duan, Y., Lai, X., Cui, S., Xu, N., ... & Lu, L. (2019). Nonpharmacological interventions for cancer‐related fatigue: a systematic review and Bayesian network meta‐analysis. Worldviews on Evidence‐Based Nursing, 16(2), 102-110. [182 RCTs included, but no clear sign of how many are CBT trials.]
4. [007] Ye, M., Du, K., Zhou, J., Zhou, Q., Shou, M., Hu, B., ... & Liu, Z. (2018). A meta‐analysis of the efficacy of cognitive behavior therapy on quality of life and psychological health of breast cancer survivors and patients. Psycho‐oncology, 27(7), 1695-1703. [10 RCTs were included for CBT on QoL and Psychological Health among breast cancer survivors and patients]
5. [009] Sun, H., Huang, H., Ji, S., Chen, X., Xu, Y., Zhu, F., & Wu, J. (2019). The efficacy of cognitive behavioral therapy to treat depression and anxiety and improve quality of life among early-stage breast cancer patients. Integrative cancer therapies, 18, 1534735419829573. [8 RCTs of CBT for D&A and QoL in early stage Breast Cancer Patients]
6. [010] Chen, D., Sun, W., Liu, N., Wang, J., Zhao, J., Zhang, Y., ... & Zhang, W. (2018, November). Fear of Cancer Recurrence: A Systematic Review of Randomized, Controlled Trials. In Oncology nursing forum (Vol. 45, No. 6). [Included 10 studies, with 1 reporting CBT for FCR]
7. [013] Johnson, J. A., Rash, J. A., Campbell, T. S., Savard, J., Gehrman, P. R., Perlis, M., ... & Garland, S. N. (2016). A systematic review and meta-analysis of randomized controlled trials of cognitive behavior therapy for insomnia (CBT-I) in cancer survivors. Sleep medicine reviews, 27, 20-28. [8 Studies CBT for Insomnia in Cancer Survivors]
8. [015] Tang, M., Liu, X., Wu, Q., & Shi, Y. (2020). The effects of cognitive-behavioral stress management for breast cancer patients: a systematic review and meta-analysis of randomized controlled trials. Cancer nursing, 43(3), 222-237. [18 RCTs of Cognitive Behavioral Stress Management for patients’ coping with stress and negative effects of cancer.]
9. [018] Zeichner, S. B., Zeichner, R. L., Gogineni, K., Shatil, S., & Ioachimescu, O. (2017). Cognitive behavioral therapy for insomnia, mindfulness, and yoga in patients with breast cancer with sleep disturbance: A literature review. Breast cancer: Basic and Clinical Research, 11, 1178223417745564. [Not perfect fit, but reference list is worth screening]
10. [019] Teo, I., Krishnan, A., & Lee, G. L. (2019). Psychosocial interventions for advanced cancer patients: A systematic review. Psycho‐oncology, 28(7), 1394-1407. [Classified 20 studies that are CBT-based]
11. [020] Matthews, H., Grunfeld, E. A., & Turner, A. (2017). The efficacy of interventions to improve psychosocial outcomes following surgical treatment for breast cancer: A systematic review and meta‐analysis. Psycho‐oncology, 26(5), 593-607. [Included 8 CBT studies should be screened for]
12. [023] Jibb, L. A., Nathan, P. C., Stevens, B. J., Yohannes, L., & Stinson, J. N. (2015, November). Psychological and physical interventions for the management of cancer-related pain in pediatric and young adult patients: an integrative review. In Oncology Nursing Forum (Vol. 42, No. 6, p. E339). Oncology Nursing Society. [Larger review, but included 9 CBT studies]
13. [024] Cobeanu, O., & David, D. (2018). Alleviation of side effects and distress in breast cancer patients by cognitive-behavioral interventions: A systematic review and meta-analysis. Journal of clinical psychology in medical settings, 25(4), 335-355. [19 to 21 clinical trials of CBT for Breast Cancer Patients]
14. [025] Tao, W. W., Tao, X. M., & Song, C. L. (2017). Effects of non-pharmacological supportive care for hot flushes in breast cancer: a meta-analysis. Supportive Care in Cancer, 25(7), 2335-2347. [Included 24 studies, but only 2 or 1 CBT studies]
15. [026] Dimitrov, L., Moschopoulou, E., & Korszun, A. (2019). Interventions for the treatment of cancer‐related traumatic stress symptoms: A systematic review of the literature. Psycho‐oncology, 28(5), 970-979. [Included 8 studies, many are CBT or CBT informed treatment]
16. [027] Xiao, F., Song, X., Chen, Q., Dai, Y., Xu, R., Qiu, C., & Guo, Q. (2017). Effectiveness of psychological interventions on depression in patients after breast cancer surgery: a meta-analysis of randomized controlled trials. Clinical breast cancer, 17(3), 171-179. [Included a total of 13 articles, and 8 or 9 of the studies were CBT related]
17. [028] Tatrow, K., & Montgomery, G. H. (2006). Cognitive behavioral therapy techniques for distress and pain in breast cancer patients: a meta-analysis. *Journal of Behavioral Medicine, 29*(1), 17-27. [Highly relevant, a total of 20 studies]
18. [029] Oh, P. J. (2016, September). The effects of nonpharmacologic interventions on cognitive function in patients with cancer: A meta-analysis. *Oncology Nursing Forum, 43*(5). [1 CBT study]
19. [030] Oh, P. J., Shin, S. R., Ahn, H. S., & Kim, H. J. (2016). Meta-analysis of psychosocial interventions on survival time in patients with cancer. Psychology & health, 31(4), 396-419. [3 CBT studies]
20. [031] Lopresti, A. L. (2017). Cognitive behaviour therapy and inflammation: a systematic review of its relationship and the potential implications for the treatment of depression. *Australian & New Zealand Journal of Psychiatry, 51*(6), 565-582. [Not directly related to cancer, but included at least 2 breast cancer related trials]
21. [032] Sheikh-Wu, S. F., Downs, C. A., & Anglade, D. (2020). Interventions for managing a symptom cluster of pain, fatigue, and sleep disturbances during cancer survivorship: A systematic review. *Oncology Nursing Forum, 47*(4), 107-119. [3 potential trials of CBT mentioned in this review]
22. [033] Ma, Y., Hall, D. L., Ngo, L. H., Liu, Q., Bain, P. A., & Yeh, G. Y. (2020). Efficacy of cognitive behavioral therapy for insomnia in breast cancer: a meta-analysis. *Sleep Medicine Reviews, 101376*. [14 RCTs of CBT for insomnia in breast cancer]
23. [035] Abrahams, H. J., Knoop, H., Schreurs, M., Aaronson, N. K., Jacobsen, P. B., Newton, R. U., ... & Buffart, L. M. (2020). Moderators of the effect of psychosocial interventions on fatigue in women with breast cancer and men with prostate cancer: Individual patient data meta‐analyses. Psycho‐Oncology, 29(11), 1772-1785. [7 CBT for fatigues in women with breast and men with prostate cancer]
24. [036] Brebach, R., Sharpe, L., Costa, D. S., Rhodes, P., & Butow, P. (2016). Psychological intervention targeting distress for cancer patients: a meta‐analytic study investigating uptake and adherence. Psycho‐Oncology, 25(8), 882-890. [2 CBT related trials for cancer in the reference list]
25. [037] Moraes, L. J., Miranda, M. B., Loures, L. F., Mainieri, A. G., & Mármora, C. H. C. (2018). A systematic review of psychoneuroimmunology-based interventions. Psychology, health & medicine, 23(6), 635-652. [2 CBT studies for cancer in the reference list]
26. [038] D’egidio, V., Sestili, C., Mancino, M., Sciarra, I., Cocchiara, R., Backhaus, I., ... & La Torre, G. (2017). Counseling interventions delivered in women with breast cancer to improve health-related quality of life: A systematic review. *Quality of Life Research, 26*(10), 2573-2592. [a couple of CBT studies, not sure how many exactly]
27. [040] Ehrenzeller, M. F., & Adam Goldstein, M. D. (2018). Smoking prevalence and management among cancer survivors. In Oncology nursing forum (Vol. 45, No. 1, pp. 55-68). Oncology Nursing Society. [a couple of relevant CBT studies for cancer smoke cessation, but not sure how many exactly]
28. [042] Hofmann, S. G., Sawyer, A. T., Witt, A. A., & Oh, D. (2010) The effect of mindfulness-based therapy on anxiety and depression: A meta-analytic review. *Journal of Consulting and Clinical Psychology, 78*(2), 169-183. [possible mindfulness CBT for cancer patients]
29. [043] Garland, S. N., Johnson, J. A., Savard, J., Gehrman, P., Perlis, M., Carlson, L., & Campbell, T. (2014). Sleeping well with cancer: a systematic review of cognitive behavioral therapy for insomnia in cancer patients. *Neuropsychiatric Disease and Treatment, 10*, 1113-1124. [CBT for insomnia for cancer patients, 13 included studies]
30. [044] Cillessen, L., Johannsen, M., Speckens, A. E., & Zachariae, R. (2019). Mindfulness‐based interventions for psychological and physical health outcomes in cancer patients and survivors: A systematic review and meta‐analysis of randomized controlled trials. *Psycho‐Oncology, 28*(12), 2257-2269. [mindfulness cognitive therapy for cancer patients]
31. [045] Petricone-Westwood, D., Jones, G., Mutsaers, B., Leclair, C. S., Tomei, C., Trudel, G., ... & Lebel, S. (2019). A systematic review of interventions for health anxiety presentations across diverse chronic illnesses. *International Journal of Behavioral Medicine, 26*(1), 3-16. [at least 3 RCTs of CBT for anxiety in patients with chronic condition]
32. [046] Haller, H., Winkler, M. M., Klose, P., Dobos, G., Kuemmel, S., & Cramer, H. (2017). Mindfulness-based interventions for women with breast cancer: an updated systematic review and meta-analysis. Acta Oncologica, 56(12), 1665-1676. [A couple of studies on mindfulness-based cognitive therapy for breast cancer patients]
33. [048] Margaretta Page, M. S. (2018). Sleep-wake disturbance: a systematic review of evidence-based interventions for management in patients with cancer. *Clinical Journal of Oncology Nursing, 22*(1), 37-52. [CBT for patients with cancer]
34. [049] Mu, P. F., Chen, Y. C., & Cheng, S. C. (2009). The effectiveness of non-pharmacological pain management in relieving chronic pain for children and adolescents. *JBI Evidence Synthesis, 7*(34), 1489-1543. [possible CBT study for young cancer survivor] [no eligible studies identified]
35. [050] MENP, K. J. M. (2016). Effective transitional therapy for adolescent and young adult patients with cancer: an integrative literature review. *Clinical Journal of Oncology Nursing, 20*(4), 391. [possible one or more CBT study for cancer]
36. [051] Fors, E. A., Bertheussen, G. F., Thune, I., Juvet, L. K., Elvsaas, I. K. Ø., Oldervoll, L., ... & Leivseth, G. (2011). Psychosocial interventions as part of breast cancer rehabilitation programs? Results from a systematic review. *Psycho‐Oncology, 20*(9), 909-918. [including CBT study for breast cancer rehabilitation]
37. [053] Chien, C. H., Liu, K. L., Chien, H. T., & Liu, H. E. (2014). The effects of psychosocial strategies on anxiety and depression of patients diagnosed with prostate cancer: A systematic review. *International Journal of Nursing Studies, 51*(1), 28-38. [include, psychosocial include CBT]
38. [054] Luckett, T., Britton, B., Clover, K., & Rankin, N. M. (2011). Evidence for interventions to improve psychological outcomes in people with head and neck cancer: a systematic review of the literature. *Supportive Care in Cancer, 19*(7), 871-881. [at least one study of cognitive therapy, likely more]
39. [055] Möller, U. O., Beck, I., Rydén, L., & Malmström, M. (2019). A comprehensive approach to rehabilitation interventions following breast cancer treatment-a systematic review of systematic reviews. *BMC Cancer, 19*(1), 1-20. [CBT study for breast cancer] [references do not include article title!]
40. [056] Coughtrey, A., Millington, A., Bennett, S., Christie, D., Hough, R., Su, M. T., ... & Shafran, R. (2018). The effectiveness of psychosocial interventions for psychological outcomes in pediatric oncology: a systematic review. *Journal of Pain and Symptom Management, 55*(3), 1004-1017. [possible CBT study]
41. [057] Fulton, J. J., Newins, A. R., Porter, L. S., & Ramos, K. (2018). Psychotherapy targeting depression and anxiety for use in palliative care: a meta-analysis. *Journal of Palliative Medicine, 21*(7), 1024-1037. [possible CBT study for pediatric cancer patients]
42. [058] Cheng, L., & Kotronoulas, G. (2020). How effective are self-management interventions in promoting health-related qualify of life in people after primary treatment for breast cancer? A critical evidence synthesis. *European Journal of Oncology Nursing,* 101776. [at least 5 CBT based studies]
43. [061] Hines, S., Ramis, M. A., Pike, S., & Chang, A. M. (2011). Effectiveness of psychosocial interventions for cognitive dysfunction in cancer patients who have received chemotherapy: A systematic review. *JBI Evidence Synthesis, 9*(49), 2023-2062. [include CBT studies for cancer patients and survivors]
44. [062] Chang, C. W., Mu, P. F., Jou, S. T., Wong, T. T., & Chen, Y. C. (2012). The effectiveness of non-pharmacological interventions on fatigue in children and adolescents with cancer: a systematic review. *JBI Evidence Synthesis, 10*(10), 574-614. [there are CBT studies for fatigue among young adults with cancer]
45. [066] Hart, S. L., Hoyt, M. A., Diefenbach, M., Anderson, D. R., Kilbourn, K. M., Craft, L. L., ... & Stanton, A. L. (2012). Meta-analysis of efficacy of interventions for elevated depressive symptoms in adults diagnosed with cancer. *Journal of the National Cancer Institute, 104*(13), 990-1004. [include a couple of CBT studies]
46. [068] Campbell, C. L., & Campbell, L. C. (2012). A systematic review of cognitive behavioral interventions in advanced cancer. *Patient Education and Counseling, 89*(1), 15-24. [11 studies met the inclusion criteria]
47. [070] Stefanopoulou, E., & Grunfeld, E. A. (2017). Mind–body interventions for vasomotor symptoms in healthy menopausal women and breast cancer survivors. A systematic review. Journal of Psychosomatic *Obstetrics & Gynecology, 38*(3), 210-225. [about 11 studies of CBT for breast cancer patients or survivors]
48. [072] Momani, T. E. G., & Berry, D. L. (2017). Integrative therapeutic approaches for the management and control of nausea in children undergoing cancer treatment: a systematic review of literature. *Journal of Pediatric Oncology Nursing, 34*(3), 173-184. [a couple of included study both in text and in the reference list]
49. [073] Ernst, E., Schmidt, K., & Baum, M. (2006). Complementary/alternative therapies for the treatment of breast cancer. A systematic review of randomized clinical trials and a critique of current terminology. *The Breast Journal, 12*(6), 526-530. [a couple of CBT studies]
50. [075] Paul, C. L., Carey, M. L., Sanson-Fisher, R. W., Houlcroft, L. E., & Turon, H. E. (2013). The impact of web-based approaches on psychosocial health in chronic physical and mental health conditions. *Health Education Research, 28*(3), 450-471. [a couple of studies]
51. [076] Mitchell, S. A., Hoffman, A. J., Clark, J. C., DeGennaro, R. M., Poirier, P., Robinson, C. B., & Weisbrod, B. L. (2014). Putting evidence into practice: an update of evidence-based interventions for cancer-related fatigue during and following treatment. *Clinical Journal of Oncology Nursing, 18*, 38-58. [several CBT studies]
52. [077] Post, K. E., & Flanagan, J. (2016). Web based survivorship interventions for women with breast cancer: An integrative review. *European Journal of Oncology Nursing, 25*, 90-99. [a couple of eligible reviews]
53. [079] O’Connor, M., & Halkett, G. K. (2019). A systematic review of interventions to reduce psychological distress in pediatric patients receiving radiation therapy. *Patient Education and Counseling, 102*(2), 275-283. [several CBT studies]
54. [080] Nenova, M., Morris, L., Paul, L., Li, Y., Applebaum, A., & DuHamel, K. (2013). Psychosocial interventions with cognitive-behavioral components for the treatment of cancer-related traumatic stress symptoms: A review of randomized controlled trials. *Journal of Cognitive Psychotherapy, 27*(3), 258-284. [clearly relevant study and will have studies for inclusion]
55. [081] Beesley, V. L., Alemayehu, C., & Webb, P. M. (2019). A systematic literature review of trials of survivorship interventions for women with gynaecological cancer and their caregivers. *European Journal of Cancer Care, 28*(3), e13057. [should be included, relevant]
56. [082] Dale, H. L., Adair, P. M., & Humphris, G. M. (2010). Systematic review of post‐treatment psychosocial and behaviour change interventions for men with cancer. *Psycho‐Oncology: Journal of the Psychological, Social and Behavioral Dimensions of Cancer, 19*(3), 227-237. [about 11 included reviews]
57. [083] Larkin, D., Lopez, V., & Aromataris, E. (2014). Managing cancer‐related fatigue in men with prostate cancer: A systematic review of non‐pharmacological interventions. *International Journal of Nursing Practice, 20*(5), 549-560. [should be included, a couple of studies]
58. [084] Osborn, R. L., Demoncada, A. C., & Feuerstein, M. (2006). Psychosocial interventions for depression, anxiety, and quality of life in cancer survivors: meta-analyses. *The International Journal of Psychiatry in Medicine, 36*(1), 13-34. [Include]
59. [087] Haerizadeh, M., Sumner, J. A., Birk, J. L., Gonzalez, C., Heyman-Kantor, R., Falzon, L., ... & Kronish, I. M. (2020). Interventions for posttraumatic stress disorder symptoms induced by medical events: A systematic review. *Journal of Psychosomatic Research, 129*, 109908. [include 1 study of EMDR vs CBT for cancer patients]
60. [088] Zee, P. C., & Ancoli-Israel, S. (2009). Does effective management of sleep disorders reduce cancer-related fatigue?. Drugs, 69(2), 29-41. [likely 4 or more studies]
61. [089] McCarter, K., Martínez, Ú., Britton, B., Baker, A., Bonevski, B., Carter, G., ... & Wolfenden, L. (2016). Smoking cessation care among patients with head and neck cancer: a systematic review. *BMJ Open, 6*(9), e012296. [1 CBT study]
62. [090] Lee, Y. H., Chiou, P. Y., Chang, P. H., & Hayter, M. (2011). A systematic review of the effectiveness of problem‐solving approaches towards symptom management in cancer care. Journal of clinical nursing, 20(1‐2), 73-85. [Problem-solving therapy for cancer survivor]
63. [091] Richardson, A. E., Broadbent, E., & Morton, R. P. (2019). A systematic review of psychological interventions for patients with head and neck cancer. *Supportive Care in Cancer, 27*(6), 2007-2021. [at least 5 RCT studies]
64. [092] Kangas, M., Bovbjerg, D. H., & Montgomery, G. H. (2008). Cancer-related fatigue: a systematic and meta-analytic review of non-pharmacological therapies for cancer patients. *Psychological Bulletin, 134*(5), 700. [19 trials reporting fatigue outcome]
65. [093] Berry, E., Davies, M., & Dempster, M. (2017). Exploring the effectiveness of couples interventions for adults living with a chronic physical illness: A systematic review. *Patient Education and Counseling, 100*(7), 1287-1303. [Include, likely have eligible study, but also likely overlapping with other studies]
66. [094] de M Alcântara‐Silva, T. R., Freitas‐Junior, R., Freitas, N. M., & Machado, G. D. (2013). Fatigue related to radiotherapy for breast and/or gynaecological cancer: a systematic review. *Journal of Clinical Nursing, 22*(19-20), 2679-2686. [include CBT study for breast cancer patients]
67. [096] Corbett, T. K., Groarke, A., Devane, D., Carr, E., Walsh, J. C., & McGuire, B. E. (2019). The effectiveness of psychological interventions for fatigue in cancer survivors: systematic review of randomised controlled trials. *Systematic Reviews, 8*(1), 1-30. [include CBT study for cancer patients and survivors]
68. [097] Hoon, L. S., Sally, C. W. C., & Hong-Gu, H. (2013). Effect of psychosocial interventions on outcomes of patients with colorectal cancer: a review of the literature. *European Journal of Oncology Nursing, 17*(6), 883-891. [include CBT study]
69. [099] Haber, J. (2004). existing evidence does not support a survival benefit for women with metastatic breast cancer who participate in group psychological interventions compared with usual care. Evidence-based nursing, 7(4), 110. [This is included, but the article is not valuable.]
70. [100] Meyer, T. J., & Mark, M. M. (1999). Effects of psychosocial interventions with adult cancer patients: A meta-analysis of randomized experiments. *Health Psychology, 14*(2), 101-106. [include a CBT category]
71. [102] Kang, H. S., Kim, H. K., Park, S. M., & Kim, J. H. (2018). Online-based interventions for sexual health among individuals with cancer: a systematic review. *BMC Health Services Research, 18*(1), 1-10. [include a couple CBT studies for cancer]
72. [103] Duijts, S. F., Faber, M. M., Oldenburg, H. S., van Beurden, M., & Aaronson, N. K. (2011). Effectiveness of behavioral techniques and physical exercise on psychosocial functioning and health‐related quality of life in breast cancer patients and survivors—a meta‐analysis. *Psycho‐Oncology, 20*(2), 115-126. [relevant, include CBT studies for cancer]
73. [106] Dy, S. M., & Apostol, C. C. (2010). Evidence-based approaches to other symptoms in advanced cancer. *The Cancer Journal, 16*(5), 507-513. [include CBT studies]
74. [107] Chien, C. H., Liu, K. L., Chien, H. T., & Liu, H. E. (2014). The effects of psychosocial strategies on anxiety and depression of patients diagnosed with prostate cancer: a systematic review. *International Journal of Nursing Studies, 51*(1), 28-38. [include CBT studies]
75. [109] Toivonen, K. I., Zernicke, K., & Carlson, L. E. (2017). Web-based mindfulness interventions for people with physical health conditions: systematic review. *Journal of Medical Internet Research, 19*(8), e303. [a couple of mindfulness CBT study for cancer patient]
76. [112] Hersch, J., Juraskova, I., Price, M., & Mullan, B. (2009). Psychosocial interventions and quality of life in gynaecological cancer patients: a systematic review. *Psycho‐Oncology: Journal of the Psychological, Social and Behavioral Dimensions of Cancer, 18*(8), 795-810. [at least 3 or more cognitive behavioral therapy]
77. [113] Tremblay, A., Sheeran, L., & Aranda, S. K. (2008). Psychoeducational interventions to alleviate hot flashes: a systematic review. Menopause, 15(1), 193-202. [possible study to be included]
78. [115] Hart, S. L., Hoyt, M. A., Diefenbach, M., Anderson, D. R., Kilbourn, K. M., Craft, L. L., ... & Stanton, A. L. (2012). Meta-analysis of efficacy of interventions for elevated depressive symptoms in adults diagnosed with cancer. *Journal of the National Cancer Institute, 104*(13), 990-1004. [a couple of CBT studies]
79. [118] Akechi, T., Okuyama, T., Onishi, J., Morita, T., & Furukawa, T. A. (2008). Psychotherapy for depression among incurable cancer patients. Cochrane Database of Systematic Reviews, (2). [a Cochrane, should be included]
80. [119] Fjorback, L. O., Arendt, M., Ørnbøl, E., Fink, P., & Walach, H. (2011). Mindfulness‐Based Stress Reduction and Mindfulness‐Based Cognitive Therapy–a systematic review of randomized controlled trials. *Acta Psychiatrica Scandinavica, 124*(2), 102-119. [mindfulness-based cognitive therapy for cancer patients]
81. [121] Tauber, N. M., O’Toole, M. S., Dinkel, A., Galica, J., Humphris, G., Lebel, S., ... & Zachariae, R. (2019). Effect of psychological intervention on fear of cancer recurrence: A systematic review and meta-analysis. Journal of Clinical Oncology, 37(31), 2899-2915. [include, contains eligible study]
82. [122 Cochrane search 1] Mustafa, M., Carson‐Stevens, A., Gillespie, D., & Edwards, A. G. (2013). [Psychological interventions for women with metastatic breast cancer](https://www.cochranelibrary.com/cdsr/doi/10.1002/14651858.CD004253.pub4/full). Cochrane Database of Systematic Reviews, (6).
83. [123 Cochrane search 2] Thabrew, H., Stasiak, K., Hetrick, S. E., Donkin, L., Huss, J. H., Highlander, A., ... & Merry, S. N. (2018). [Psychological therapies for anxiety and depression in children and adolescents with long‐term physical conditions](https://www.cochranelibrary.com/cdsr/doi/10.1002/14651858.CD012488.pub2/full). Cochrane Database of Systematic Reviews, (12).
84. [124 Cochrane search 3] Jassim, G. A., Whitford, D. L., Hickey, A., & Carter, B. (2015). [Psychological interventions for women with non‐metastatic breast cancer](https://www.cochranelibrary.com/cdsr/doi/10.1002/14651858.CD008729.pub2/full). Cochrane Database of Systematic Reviews, (5).
85. [125 Cochrane search 4] Law, E., Fisher, E., Eccleston, C., & Palermo, T. M. (2019). [Psychological interventions for parents of children and adolescents with chronic illness.](https://www.cochranelibrary.com/cdsr/doi/10.1002/14651858.CD009660.pub4/full) Cochrane Database of Systematic Reviews, (3).
86. [126 Cochrane search 5] Thabrew, H., Stasiak, K., Hetrick, S. E., Wong, S., Huss, J. H., & Merry, S. N. (2018). E‐Health interventions for anxiety and depression in children and adolescents with long‐term physical conditions. Cochrane Database of Systematic Reviews, (8).
87. [130 Cochrane search 8] Uphoff, E., Pires, M., Barbui, C., Barua, D., Churchill, R., Cristofalo, D., ... & Siddiqi, N. (2020). Behavioural activation therapy for depression in adults with non‐communicable diseases. Cochrane Database of Systematic Reviews, (8).
88. [132 Cochrane search 11] Clarkson, J. E., Worthington, H. V., Furness, S., Mccabe, M., Khalid, T., & Meyer, S. (2010). Interventions for treatment oral mucositis for patients with cancer receiving treatment. Cochrane Database of Systematic Reviews, (8).
89. [133 Cochrane search 12] Poort, H., Peters, M., Bleijenberg, G., Gielissen, M. F., Goedendorp, M. M., Jacobsen, P., ... & Knoop, H. (2017). Psychosocial interventions for fatigue during cancer treatment with palliative intent. Cochrane Database of Systematic Reviews, (7).
90. [137 Cochrane search 16] Parahoo, K., McDonough, S., McCaughan, E., Noyes, J., Semple, C., Halstead, E. J., ... & Dahm, P. (2013). Psychosocial interventions for men with prostate cancer. Cochrane Database of Systematic Reviews, (12).
91. [140 Cochrane search 19] Cheng, K. K. F., Lim, Y. T. E., Koh, Z. M., & San Tam, W. W. (2017). Home‐based multidimensional survivorship programmes for breast cancer survivors. Cochrane Database of Systematic Reviews, (8).

**Four Professional Websites:**

Association for Behavioral and Cognitive Therapies: <https://www.abct.org/>

Association for Clinical Oncology: <https://www.asco.org/about-asco/about-asco-association>

International Psycho-Oncology Society: <https://www.ipos-society.org/>

Association of Oncology Social Work: <https://aosw.org/>
